# Activity‐Based Protein Profiling Identifies Protein Disulfide‐Isomerases as Target Proteins of the Volatile Salinilactones

**DOI:** 10.1002/advs.202309515

**Published:** 2024-03-02

**Authors:** Karoline Jerye, Helko Lüken, Anika Steffen, Christian Schlawis, Lothar Jänsch, Stefan Schulz, Mark Brönstrup

**Affiliations:** ^1^ Department of Chemical Biology Helmholtz Centre for Infection Research Inhoffenstraße 7 38124 Braunschweig Germany; ^2^ Department of Cell Biology Helmholtz Centre for Infection Research Inhoffenstraße 7 38124 Braunschweig Germany; ^3^ Institute of Organic Chemistry Technische Universität Braunschweig Hagenring 30 38106 Braunschweig Germany; ^4^ Research Group Cellular Proteome Research Helmholtz Centre for Infection Research Inhoffenstraße 7 38124 Braunschweig Germany; ^5^ Biomolecular Drug Research Center (BMWZ) Leibniz Universität Hannover Schneiderberg 1B 30167 Hannover Germany; ^6^ German Center for Infection Research Site Hannover‐Braunschweig Inhoffenstraße 7 38124 Braunschweig Germany

**Keywords:** activity‐based protein profiling, natural products, protein disulfide‐isomerases, salinilactones, volatiles

## Abstract

The salinilactones, volatile marine natural products secreted from *Salinispora arenicola*, feature a unique [3.1.0]‐lactone ring system and cytotoxic activities through a hitherto unknown mechanism. To find their molecular target, an activity‐based protein profiling with a salinilactone‐derived probe is applied that disclosed the protein disulfide‐isomerases (PDIs) as the dominant mammalian targets of salinilactones, and thioredoxin (TRX1) as secondary target. The inhibition of protein disulfide‐isomerase A1 (PDIA1) and TRX1 is confirmed by biochemical assays with recombinant proteins, showing that (1*S*,5*R*)‐salinilactone B is more potent than its (1*R*,5*S*)‐configured enantiomer. The salinilactones bound covalently to C53 and C397, the catalytically active cysteines of the isoform PDIA1 according to tandem mass spectrometry. Reactions with a model substrate demonstrated that the cyclopropyl group is opened by an attack of the thiol at C6. Fluorophore labeling experiments showed the cell permeability of a salinilactone‐BODIPY (dipyrrometheneboron difluoride) conjugate and its co‐localization with PDIs in the endoplasmic reticulum. The study is one of the first to pinpoint a molecular target for a volatile microbial natural product, and it demonstrates that salinilactones can achieve high selectivity despite their small size and intrinsic reactivity.

## Graphical abstract

Molecular targets of volatile natural products are largely unknown. The identification of protein disulfide‐isomerases (PDIs) are presented as a target protein family of the salinilactones, volatile metabolites produced by marine *Salinispora* bacteria. The unnatural (1*S*,5*R*)‐salinilactone B enantiomer exhibits high target selectivity by binding covalently to protein disulfide‐isomerase A1 (PDIA1) at C53 and C397 residues and localizes at the endoplasmic reticulum of A549 cells.

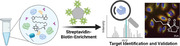

## 1 Introduction

**Figure 1:**
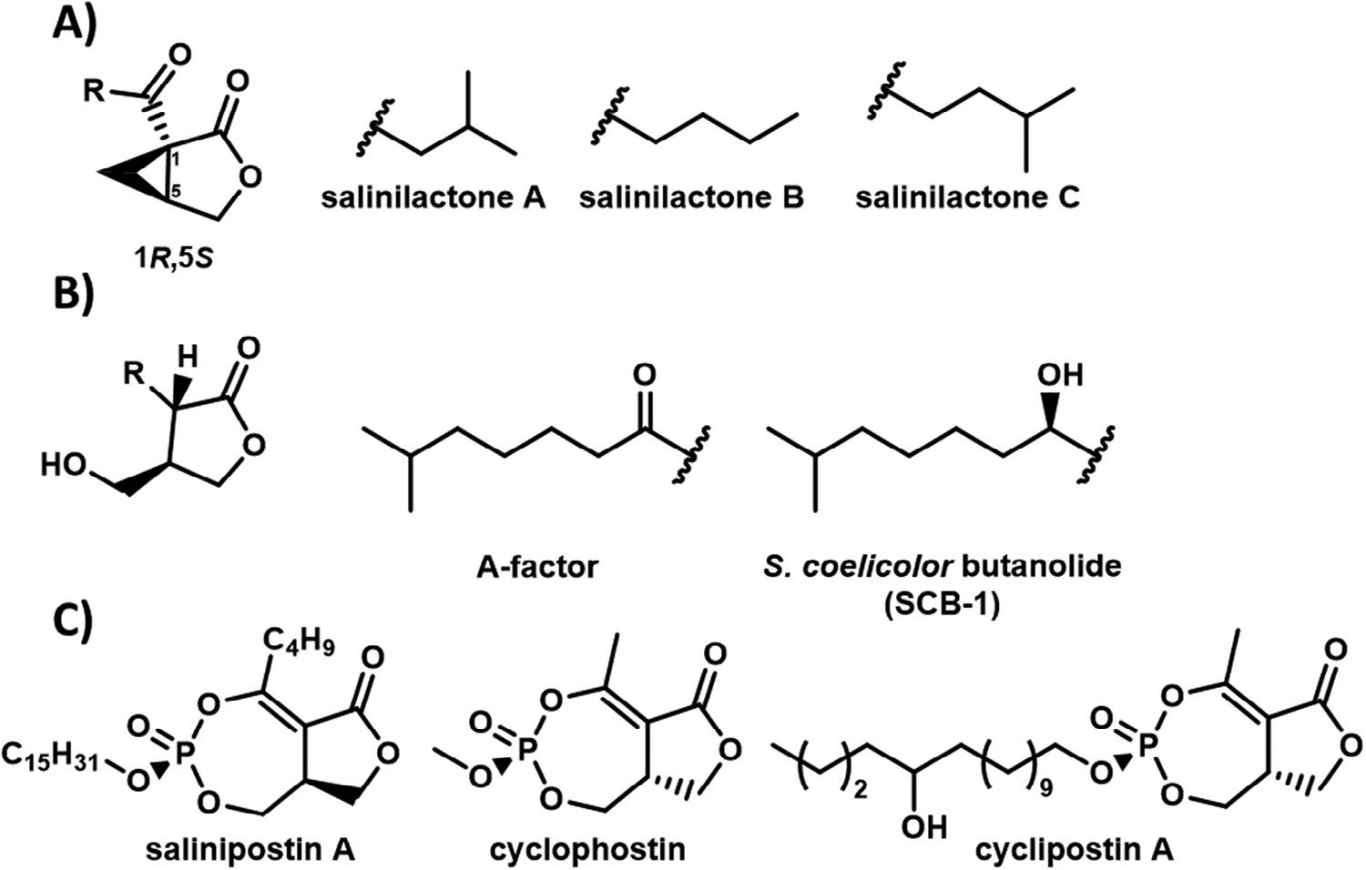
Structures of A) (1*R*,5*S*)‐salinilactones A‐C; B) γ‐butyrolactones; C) salinipostin A, cyclophostin and cyclipostin A.

Marine organisms are a prolific source of natural products with unique chemical scaffolds^[^ ^1^ ^]^ and high bioactivities of relevance for human disease, as demonstrated by 20 approved drugs with marine roots.^[^ ^2^ ^]^ Among cultivable marine bacteria, the genus *Salinispora* gained attention as a producer of diverse and bioactive chemicals such as salinosporamide A,^[^ ^3^, ^4^, ^5^ ^]^ a proteasome inhibitor featuring a reactive ß‐lactone that is tested in a phase III clinical trial against glioblastoma (NCT03345095). Some *Salinispora* bacteria also biosynthesize a second family of strained lactones named salinilactones. They were discovered from volatiles released by *Salinispora arenicola* and have a unique [3.1.0]‐lactone ring system (**Figure** **1**A).^[^ ^6^, ^7^ ^]^ With a ratio of 97:3, the (1*R*,5*S*)‐enantiomer was found to be far more abundant than the (1*S*,5*R*)‐enantiomer. In phenotypic assays, antibacterial effects against *Salinispora* as well as non‐*Salinispora* actinomycetes were found.^[^ ^7^ ^]^ In addition, salinilactones displayed cytotoxicity in brine shrimp assays, indicating a potential defensive function against grazers.^[^ ^7^ ^]^ A recent study reported cytotoxic activities of *Salinispora arenicola* SH‐78 extracts containing salinilactones and staurosporines against HCT‐116 and MDA‐MB‐231 cancer cell lines.^[^ ^8^ ^]^ While the interest in volatile natural products of bacteria has sparked significantly in recent years, knowledge about their molecular targets is non‐existent.^[^ ^9^, ^10^ ^]^ Challenged by this gap, we embarked on finding the molecular target(s) of salinilactones, starting from three speculative considerations: Given their structural similarity to the A‐factor, part of the γ‐butyrolactone family (autoregulators) (Figure 1B), a similar signaling function was proposed for salinilactones.^[^ ^7^ ^]^ A second, structurally related class are the salinipostins, also isolated from a marine *Salinispora* sp. bacterium,^[^ ^11^ ^]^ that might be either derived from, or precursors of salinilactones by the addition (or elimination, respectively) of an alkyl phosphate. The non‐volatile salinipostins exhibited highly potent antimalarial activity, but a molecular target was not reported; however, the closely related cyclipostins and cyclophostin, isolated from terrestrial *Streptomyces* sp., were potent inhibitors of hormone‐sensitive lipase and other serine hydrolases; notably, their configuration at the methine moiety is reversed (Figure 1C).^[^ ^12^, ^13^, ^14^ ^]^ Finally, the electrophilic character of the keto‐substituted [3.1.0]‐lactone moiety implied that salinilactones might directly react with protein nucleophiles.^[^ ^15^, ^16^, ^17^ ^]^ In this respect, an open question was whether the absence of selectivity‐conferring large substituents in salinilactones may lead to unspecific reactivity. In this study, our efforts to find and validate the target(s) of the salinilactones are reported.

## 2 Results and Discussion

**Figure 2:**
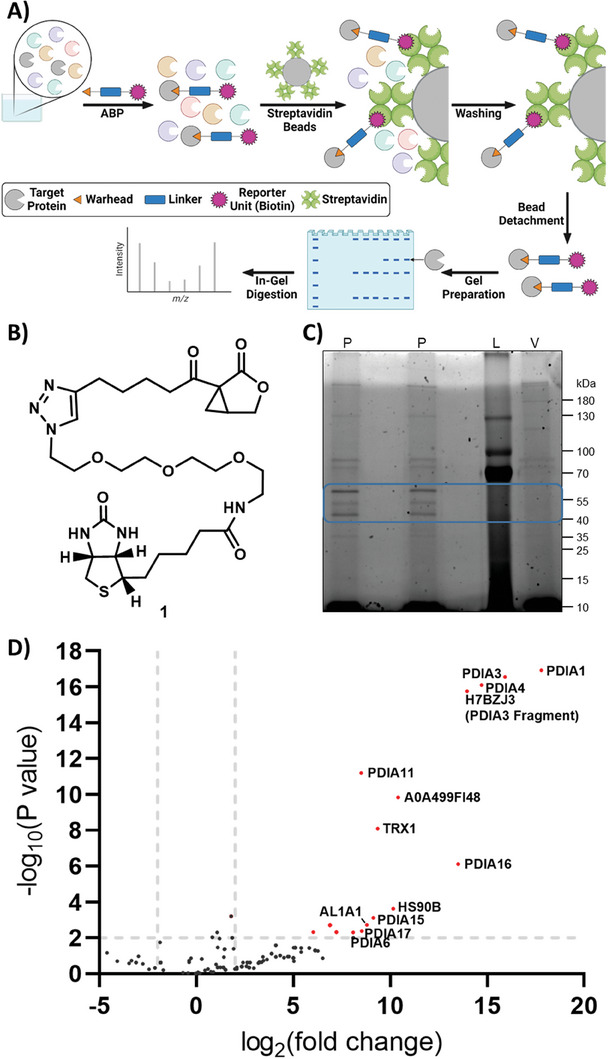
Target identification by activity‐based protein profiling (ABPP). A) Workflow. ABP = activity‐based probe. Graphic created with BioRender. B) Structure of the activity‐based salinilactone probe **1**. C) Sodium dodecyl sulfate (SDS) gel stained with 2,2,2‐trichloroethanol after protein enrichment. The blue box surrounds the largest differences between ABP‐treated (n = 2) versus vehicle‐treated conditions (n = 1). P = activity‐based salinilactone probe **1**, L = gel ladder, V = vehicle. D) Volcano plot of proteins identified by tandem mass spectrometry after enrichment by ABPP applying the SP3 protocol. The threshold for significantly enriched proteins was set to *P* = 0.01 and log_2_(fold change) = ±2 and is visualized with dashed grey lines. Significantly enriched proteins are shown in red. Positive control: n = 6; negative control: n = 4.

We chose activity‐based protein profiling (ABPP) as an unbiased method for target finding^[^ ^18^, ^19^ ^]^ using the [3.1.0]‐lactone moiety of salinilactone as a reactive warhead. Given preliminary structure‐activity relationships for salinilactones,^[^ ^7^ ^]^ we assumed that the alkyl side chain was of limited relevance for activity. Therefore, a salinilactone analog with an alkynyl‐terminal moiety was prepared as a mixture of (1*S*,5*R*)­ and (1*R*,5*S*)­enantiomers^[^ ^20^ ^]^ and extended by a polar linker through a copper‐catalyzed azide‐alkyne cycloaddition (CuAAC) reaction to give the activity‐based probe **1** (**Figure** **2**B). The linker ended with a biotin moiety to enable the enrichment of target proteins.^[^ ^21^, ^22^ ^]^ In the ABPP workflow pursued by us, a lysate of A549 lung epithelial cells was incubated with **1** for 5 h (Figure 2A). After the binding of the probe to its target proteins, the complexes were separated from the supernatant by streptavidin‐coated Sepharose beads. After washing and centrifugation steps, denaturing conditions released the probe‐bound target proteins from the beads. A first visualization of enriched proteins was done via a SDS‐PAGE (sodium dodecyl sulfate‐polyacrylamide gel electrophoresis) with 2,2,2‐trichloroethanol. Compared to the vehicle control, we observed an enrichment of at least three bands, with two prominent bands at ≈60 and 50 kDa (Figure 2C). To identify the underlying proteins, the enriched bands were in‐gel digested, followed by a tandem mass spectrometry (MS/MS) analysis. The upper band was identified as the protein disulfide‐isomerases (PDIs) PDIA1 and PDIA3, while the lower band was identified as PDIA6 and PDIA15 (Figure S1, Supporting Information). In a second experiment, the SP3 (single‐pot, solid‐phase‐enhanced sample preparation for proteomics experiments) protocol, that involved paramagnetic bead‐based protein enrichment,^[^ ^23^ ^]^ was applied before the mass spectrometric analysis. This led to 16 strongly enriched proteins visualized in a volcano plot (Figure 2D). This experiment also revealed the PDIs as the predominant target proteins of the salinilactones, as a broad range of isoforms, comprising PDIA1, PDIA3, PDIA4, PDIA6, PDIA11, PDIA15, PDIA16 and PDIA17, were identified through unique peptides (Figure S2, Supporting Information).

PDIs show a high sequence conservation, contain one or multiple so‐called thioredoxin‐like domains, and share a catalytic center with a CXXC motive (Figure S3, Supporting Information). PDIs have raised a lot of interest as promising, druggable targets, because isoforms such as PDIA1, PDIA3, PDIA4, and PDIA6 are overexpressed in many cancer types.^[^ ^24^ ^]^

In order to study the influence of the stereochemistry of the salinilactones on binding to the target proteins, both enantiomers were required in pure form. We modified the first stereoselective salinilactone synthesis, which led to moderate enantiomeric excesses of 37% for (1*R*,5*S*)‐salinilactone B **9** and 52% for its (1*S*,5*R*)‐analog **8**, respectively.^[^ ^6^, ^20^ ^]^

**Scheme 1:**
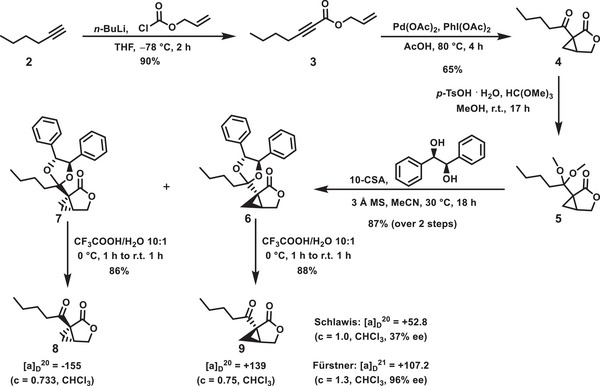
Synthesis of salinilactone B enantiomers. THF = tetrahydrofuran, 10‐CSA = camphorsulfonic acid, 3 Å MS = 3 Å molecular sieves, ee = enantiomeric excess. Specific rotations were compared to literature data.^[^ ^20^, ^25^ ^]^

To enable a chiral resolution of diastereomers, racemic salinilactone B **4** was reacted with (*R*,*R*)‐(+)‐hydrobenzoin as a chiral auxiliary (**Scheme** **1**). This was only possible after synthesizing the more reactive acetal intermediate **5**, since dioxolane formation could not be observed in the direct reaction of the ketone derivative **4** with the diol. The separation of the diastereomers **6** and **7** was achieved by neutral reversed‐phase HPLC (high‐performance liquid chromatography). The following removal of the chiral auxiliary with a 10:1 trifluoroacetic acid/water mixture yielded both enantiomerically pure products, that showed specific optical rotations of [α]_D_^20^ = +139 for (1*R*,5*S*)‐salinilactone B **9** and −155 for (1*S*,5*R*)‐salinilactone B **8**, respectively.

**Figure 3:**
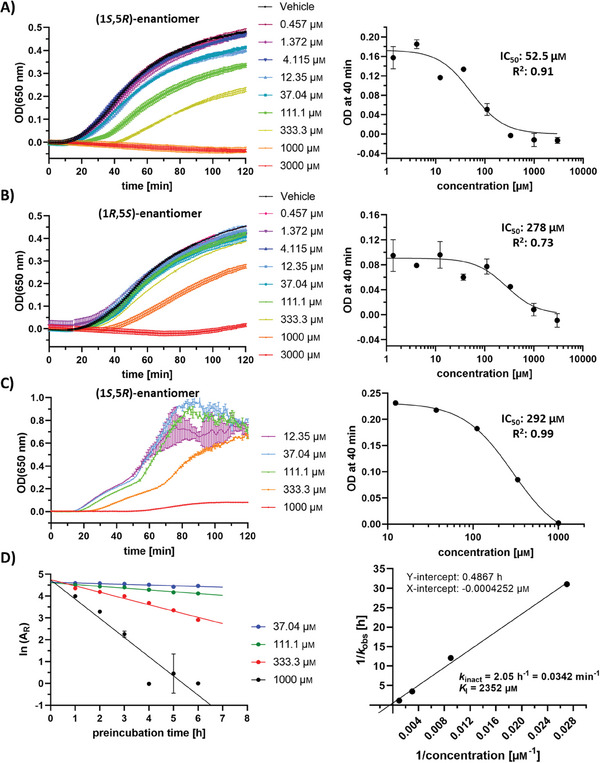
Validation of salinilactone B targets by biochemical assays. A,B) Inhibition of PDIA1‐mediated insulin reduction after 6 h preincubation with salinilactone B. Time‐dependent turbidity plots are shown on the left, and calculated IC_50_ curves after 40 min on the right (mean of n = 3 ± SEM). A) (1*S*,5*R*)‐enantiomer **8**. For c = 37.04 µm one of the triplicates was excluded because almost no increasing turbidity was measured in contrast to the other ones. B) (1*R*,5*S*)‐enantiomer **9**. C) Inhibition of TRX1‐mediated insulin reduction by **8** after 5 h preincubation. Time‐dependent turbidity plots are shown on the left, and calculated IC_50_ curves after 40 min on the right (mean of n = 2 ± SEM). OD(650 nm) = optical density measured at *λ* = 650 nm. D) Determination of *k*_inact_ and *K*_I_ for **8** depending on the preincubation time with PDIA1. The remaining enzyme activity (A_R_) at 40 min plotted against the preincubation time is shown on the left (mean of n = 2 ± SEM), and the double reciprocal plot of *k*_obs_ and the inhibitor concentration on the right.

The conserved catalytic sites of PDIs containing two cysteines led us assume that the salinilactones inhibit the enzymatic function of these proteins by covalently binding to the nucleophilic sulfur atom. Since PDIA1 was found to be the main target, a functional insulin reduction assay with recombinant PDIA1 produced in *E. coli* was established. The assay captures the enzymatic activity of PDIs by their ability to reduce the A‐ and B‐chain of insulin in the presence of dithiothreitol, resulting in an increasing turbidity at a wavelength of *λ* = 650 nm.^[^ ^26^ ^]^ We found a clear inhibiting effect of (1*S*,5*R*)‐salinilactone B **8** on PDIA1 following a preincubation time of 6 h with an half maximal inhibitory concentration (IC_50_) value of 53 µm. (1*R*,5*S*)‐Enantiomer **9** had lower potency with an IC_50_ value of 278 µm. Notably, more potent (1*S*,5*R*)‐enantiomer **8** is far less abundant in nature (3% vs 97%).^[^ ^6^ ^]^ This reflects that the ecological function of the salinilactones is very likely not to inhibit human or animal PDIs. The determination of the rate of covalent modification was carried out with **8**, resulting in a *k*_inact_/*K*_I_ of 1.45 × 10^−5^ µm ^−1^ min^−1^ (**Figure** **3**D; Chapter S4, Supporting Information).

Since the ABPP experiment identified TRX1 as another potential target protein, the inhibitory effects of the salinilactone B enantiomers were also investigated in a similar insulin reduction assay with recombinant human TRX1 obtained from *E. coli*. Again, a dose‐dependent inhibition was observed, with an IC_50_ value of 292 µm for (1*S*,5*R*)‐enantiomer **8** (Figure 3C; Figure S4, Supporting Information). However, the inhibition efficacy was weaker compared to that on PDIA1. Thus, human thioredoxin was validated in a functional assay as a second target of salinilactones, reflecting sequence conservation of 50% in a 20 amino acid long region (V388‐W407) around the second catalytic active site of PDIA1. A third protein identified by chemoproteomics, the aldehyde dehydrogenase (AL1A1), was weakly inhibited by salinilactone in a functional assay in vitro (see Chapter S5, Supporting Information).

**Figure 4:**
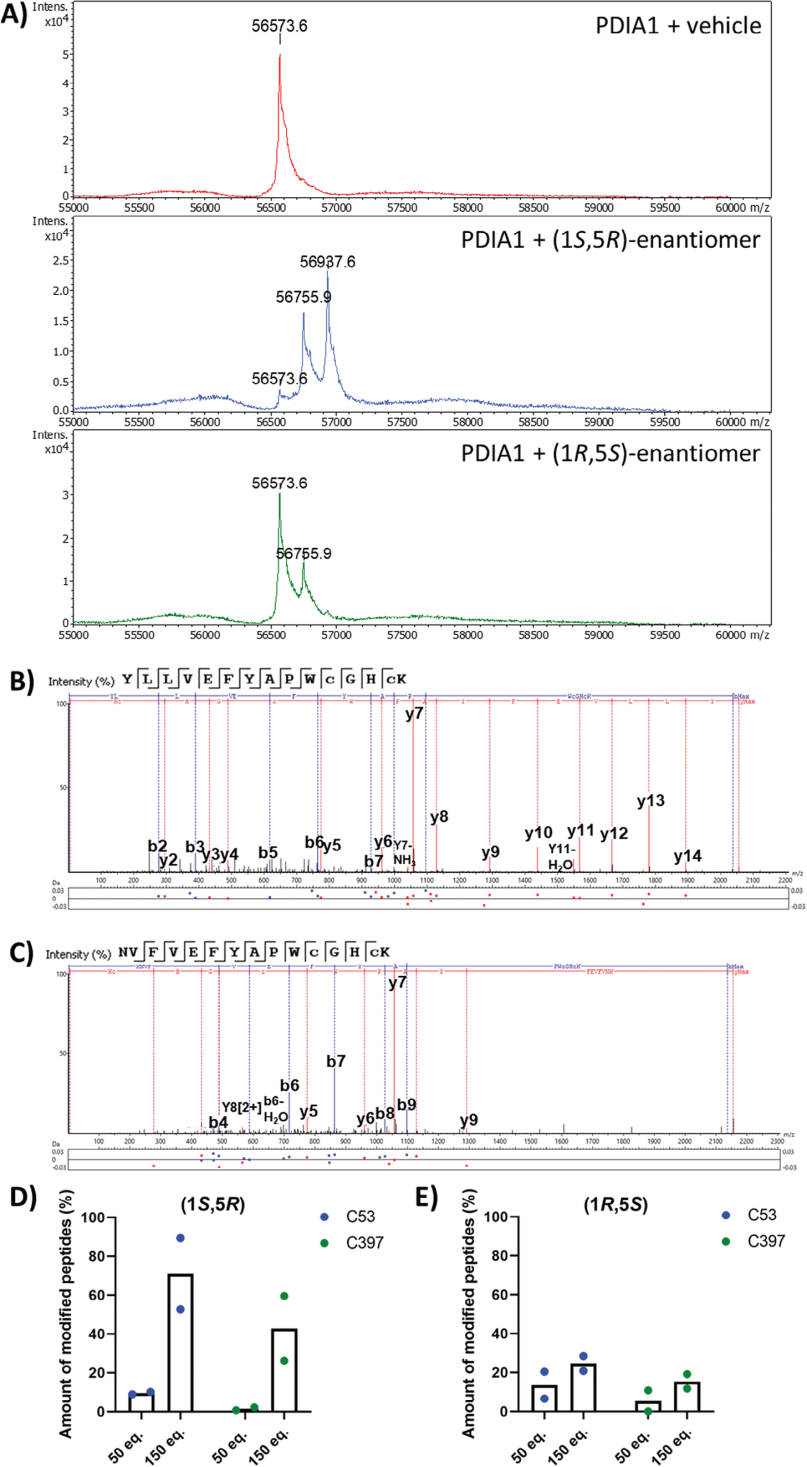
Covalent modification of PDIA1 with salinilactone B. A) Intact protein mass spectra. The unmodified protein (200 µg mL^−1^) had a total mass of 56573 Da (top red trace). The addition of (1*S*,5*R*)‐salinilactone B **8** (1 mm) resulted in two protein peaks corresponding to modification by one (+182 Da) or two (+364 Da) molecules (middle blue trace). The addition of (1*R*,5*S*)‐salinilactone B **9** (1 mm) resulted in a modification by one molecule (+182 Da, lower green trace). Each condition was carried out with n = 1. MS spectra were deconvoluted with the MaxEnt software (charge z = 1). B,C) Detection of modified peptides after incubation of PDIA1 with 150 eq. of **8** and tryptic digestion (n = 2). Assigned fragments are indicated in the spectrum, and mass deviations are plotted below. B) MS/MS spectrum of peptide YLLVEFYAPWcGHCK, showing a modification at C53. C) MS/MS spectrum of peptide NVFVEFYAPWcGHCK, showing a modification at C397. D,E) Degree of peptide modifications following incubation with 50 and 150 equivalents (eq.) of **8** (D) and **9** (E) (n = 2).

We focused further investigations on the primary target protein PDIA1. To check the hypothesis that salinilactone B inhibits PDIA1 by covalently reacting with active site cysteine(s), a mass spectrometric analysis of the intact protein incubated with salinilactone B was carried out following denaturing chromatography and electrospray ionization. The mass spectra of PDIA1 (200 µg mL^−1^) incubated with (*1S,5R*)‐enantiomer **8** (1000 µm) showed the depletion of unmodified protein and two new peaks, that correspond to protein modifications with one (+182 Da) or two (+364 Da) salinilactone B molecules (**Figure** **4**A). In contrast, incubation with (1*R*,5*S*)‐enantiomer **9** resulted in a high amount of unmodified PDIA1, plus a single salinilactone modification (+182 Da). Thus, the difference in the inhibitory potency of the two enantiomers could also be recognized there. The occurrence of maximally two covalently bonded salinilactone B molecules suggested that binding occurred at the two catalytic cysteine residues.

**Scheme 2:**
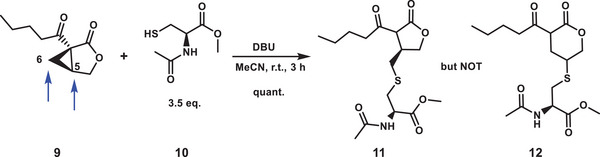
Ring‐opening reaction of (1*R*,5*S*)‐salinilactone B **9** with *N*‐acetyl‐cysteine methyl ester **10** to elucidate the enzyme binding. DBU = 1,8‐diazabicyclo[5.4.0]undec‐7‐ene. Blue arrows indicate two possible sites of attack.

To locate the binding sites more precisely down to the amino acid level, a tandem mass spectrometry (MS/MS) analysis of PDIA1‐derived peptides obtained by tryptic digestion was performed. Both salinilactone B enantiomers bound covalently to C53 and C397, which are part of the catalytic CXXC motive (primary accession no: P07237) (Figure 4B–E; Chapter S7, Supporting Information). In contrast, a modification of the adjacent C56 or C400 residues was not observed. This finding is consistent with reports that the more *N*‐terminal cysteines of the active sites (C53 and C397 in PDIA1) are much more nucleophilic due to their extraordinarily low p*K*_a_ value of 4.8, whereas cysteines closer to the *C*‐terminal end (C56 and C400 in PDIA1) have a p*K*_a_ value >10.^[^ ^27^ ^]^ (1*S*,5*R*)‐Salinilactone B **8** modified C53 to a larger extent than C397 (Figure 4D). It was more reactive than (1*R*,5*S*)‐enantiomer **9** (Figure 4E), in line with the data from functional enzymatic assays and full protein mass spectrometry. We assume that C53 and C397 are the only binding sites, because the degree of their modification was concentration‐dependent, while other putatively assigned binding sites showed no concentration dependence and mostly no convincing MS/MS spectra. The nucleophilic attack of reactive cysteines might occur at two different positions of the [3.1.0]‐lactone, either at C5 to give a δ‐lactone or at C6 to give a γ‐lactone (**Scheme** **2**). The regioselectivity was analyzed using (1*R*,5*S*)‐salinilactone B **9** and *N*‐acetyl‐cysteine methyl ester **10** as a model nucleophile. The latter was deprotonated by 1,8‐diazabicyclo[5.4.0]undec‐7‐ene (DBU). NMR experiments clearly showed the formation of the five‐membered ring **11**, while the formation of the six‐membered ring **12** was not observed (Chapter S11, Supporting Information). The findings are in line with literature data on nucleophilic ring openings by methanol^[^ ^28^ ^]^ or different thiols^[^ ^29^ ^]^ on related, but differently substituted bicycles.

**Scheme 3:**
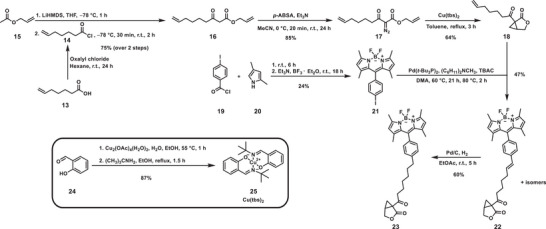
Synthesis of the BODIPY‐salinilactone conjugate **23**. The synthesis of the Cu(tbs)_2_ catalyst is shown in the black box. LiHMDS = lithium‐bis(trimethylsilyl)amid, THF = tetrahydrofuran, *p*‐ABSA = 4‐acetamidobenzenesulfonyl azide, TBAC = tetrabutylammonium chloride, DMA = dimethylacetamide, BODIPY = dipyrrometheneboron difluoride.

In order to visualize the specific binding of the salinilactones to their targets by fluorescence microscopy, a conjugate of racemic salinilactone with the fluorophore BODIPY (borondipyrromethene difluoride) was synthesized (**Scheme** **3**). The 1,3‐dicarbonyl **16** was synthesized by coupling allyl acetate (**15**) with hept‐6‐enoyl chloride (**14**) and further reacted with 4‐acetamidobenzene‐sulfonyl azide to give the diazo compound **17**. The following cyclization turned out to be more difficult to accomplish than expected. The usage of rhodium catalysts like Rh_2_(OAc)_4_, Rh_2_(esp)_2_, Rh_2_(TPA)_4_ and Rh_2_(*S*‐TCPTAD)_4_ resulted only in small amounts of the desired product, because of an unwanted reaction with the hexenyl chain of the carboxylic acid. Cobalt catalysts such as cobalt (II) porphyrin or salcomine performed better, but harsh conditions were needed, and yields were still low.

The key was to use Cu(tbs)_2_, which acts as a catalyst for the cyclization reaction with the allylic double bond to the desired alkenyl‐modified salinilactone **18** with a yield of 65%. A Pd‐catalyzed Heck reaction^[^ ^30^ ^]^ served to couple **18** with the BODIPY fluorophore **21** bearing an aryl iodide^[^ ^31^ ^]^ and led to cross‐coupled products in a yield of 47%. The double bond was reduced in the final step with Pd/C to give the BODIPY‐salinilactone conjugate **23** in 60% yield.

**Figure 5:**
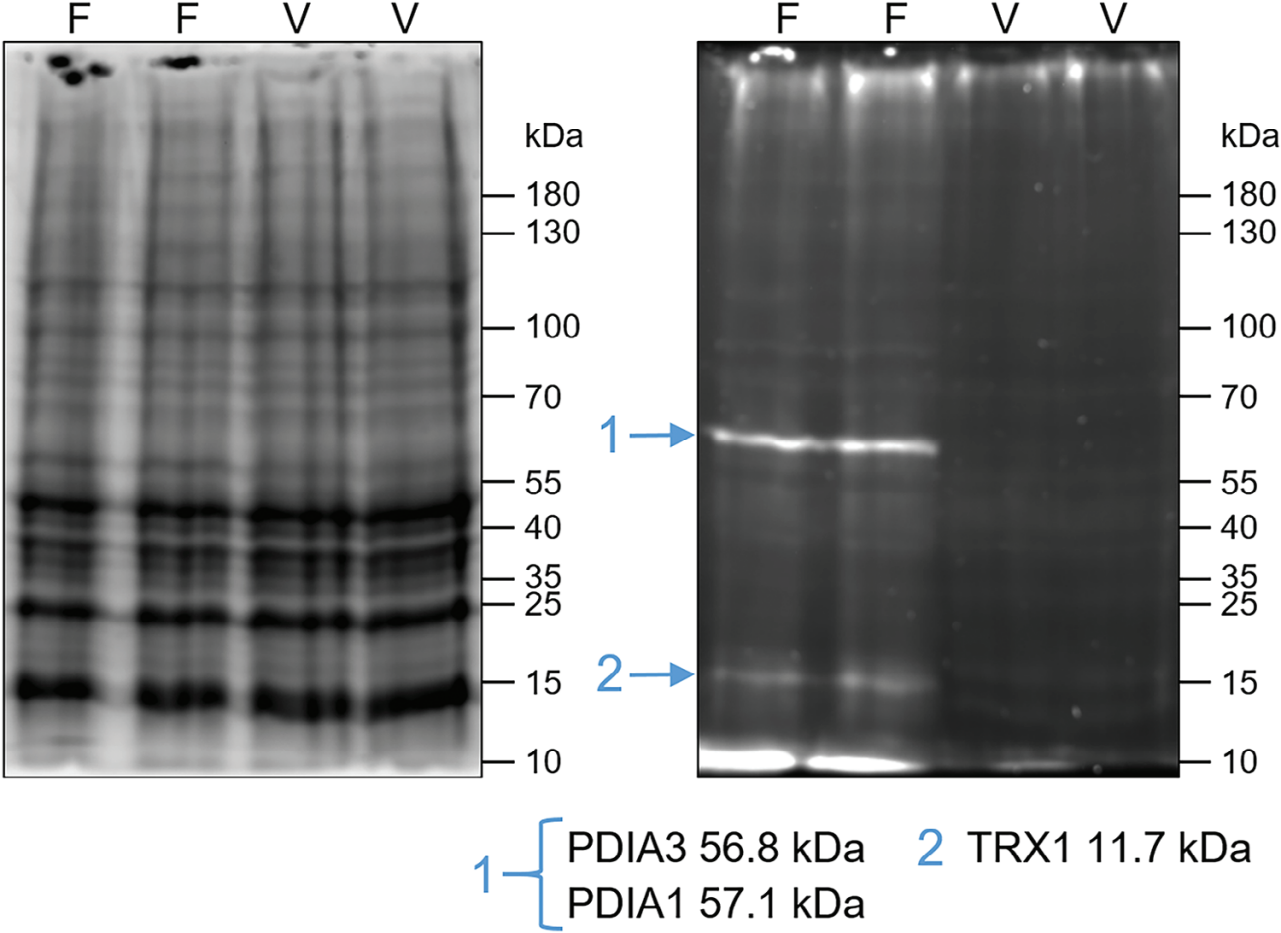
Protein labeling and visualization with the BODIPY‐salinilactone conjugate **23**. A549 cells were incubated with **23** (n = 2) or vehicle control (n = 2) for 18 h and lysed. The protein bands on the left gel were stained by 2,2,2‐trichloroethanol with comparable total protein abundance in treated versus untreated samples. Fluorescence detection of the right gel (*λ*_ex_ = 512 nm and *λ*_em_ = 535 nm) detected bands of proteins modified with **23**. Tentative assignment of protein bands is shown below, with protein masses obtained from UniProt. F = Fluorophore salinilactone conjugate **23**, V = Vehicle.

To visualize the binding to the target proteins, **23** and a vehicle control were incubated with A549 cells. Two SDS gels with different visualization conditions demonstrated: i) effective cell lysis with no difference between the samples with and without **23**, and ii) the specific binding of the fluorescent probe **23** to only few proteins (**Figure** **5**). Comparison of the main band with the gel ladder indicates that it is approximately equal to the molecular masses of PDIA1 (57.1 kDa) and PDIA3 (56.8 kDa), and a second band might correspond to TRX1 (11.7 kDa). The presence of only few bands confirmed the results of the chemoproteomic approach, again underlining the remarkably high target specificity of salinilactones.

**Figure 6:**
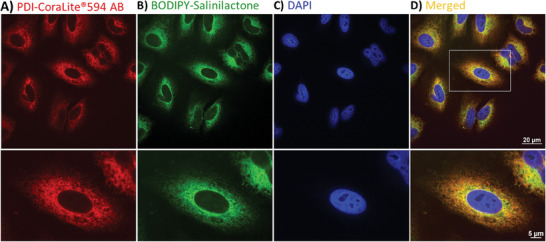
Subcellular localization of the fluorophore‐tagged salinilactone **23** in A549 cells. Cells were incubated for 18 h with BODIPY‐salinilactone **23** prior to fixation and staining using CoraLite594 fluorophore‐tagged anti‐PDI monoclonal antibody. Images show a single spinning disk confocal plane of A) CoraLite594, B) fluorescence signal of **23** (*λ*_em_ = 525/50 nm) and C) DAPI staining to visualize nuclei. DAPI = 4′,6′‐diamidino‐2‐phenylindole D) The overlay of signals from (A–C) shows the co‐localization of the PDI antibody with **23**.

Protein disulfide‐isomerases are mainly located in the endoplasmic reticulum (ER) of the cells.^[^ ^32^ ^]^ In order to investigate the localization and target engagement of salinilactones in a cellular environment, A549 cells were incubated with the fluorophore‐tagged salinilactone **23** for 18 h and visualized by fluorescence microscopy after counterstaining with a PDI‐targeting antibody (**Figure** **6**). As expected, the intracellular PDI was detected in the ER, as visible from (i) a continuous fluorescent circle around the nucleus resulting from the membrane of the ER adjacent to the outer membrane of the nucleus and (ii) the typical reticulate pattern of the ER characterized by “holes” without any fluorescence, resulting from the tubules of the ER (Figure 6A).

Interestingly, the BODIPY‐salinilactone **23** showed a clear overlap with intracellular PDI (Figure 6A/B/D). The absence of a nuclear signal for **23** speaks against an interaction with DNA. Together, these experiments demonstrate the cell permeability of probe **23** and its localization to the ER. They also imply that salinilactones target PDIs within cells.

## 3 Conclusion 

In summary, the protein disulfide‐isomerases (PDIs) were successfully identified as the targets of the marine natural products salinilactones. Activity‐based protein profiling (ABPP) was applied as the discovery technique, and residues C53 and C397, located in the two catalytically active sites of PDIA1, were found to be covalently modified by salinilactone B.

The protein disulfide‐isomerases, and mainly the PDIA1 isoform, have been intensely studied as anticancer targets,^[^ ^24^ ^]^ but they were also found relevant in other indications and processes such as thrombosis, neurodevelopment, viral entry, or ferroptosis.^[^ ^33^, ^34^, ^35^, ^36^ ^]^ A series of PDI inhibitors targeting the catalytically active as well as allosteric^[^ ^37^, ^38^ ^]^ residues have been reported, among them the in vivo active prototype inhibitor PACMA 31.^[^ ^39^ ^]^ Compared to optimized PDI inhibitors, natural salinilactones are less potent. However, the synthetic routes described here and by Fürstner and coworkers^[^ ^25^ ^]^ enable the modulation of the compound's potency; for example, an improvement could be attempted by modifying the lipid side chain. The ability of salinilactones to bind multiple isoforms constitutes an advantage, because it was shown that the individual knockdown of some isoforms may be compensated by other members of the PDI family,^[^ ^40^, ^41^, ^42^ ^]^ rendering a pan‐style inhibitor attractive.^[^ ^40^ ^]^ Furthermore, the small size and apolar nature of salinilactones is special among PDI inhibitors and might be beneficial for reaching the central nervous system.^[^ ^43^ ^]^ The salinilactones add to the yet limited arsenal of natural products with electrophilic cyclopropanes, with few, but very prominent congeners such as duocarmycin, illudin, or colibactin.^[^ ^17^, ^44^, ^45^ ^]^ In all cases, the cyclopropane is activated through adjacent, direct or vinylogous electrophilic centers, and the [3.1.0]‐lactone of salinilactones represents a new motif to enhance electrophilicity. To the best of our knowledge, this study is among the first to describe a molecular target for a volatile microbial natural product from bacteria.^[^ ^10^, ^46^ ^]^ The interest in volatile natural products has recently increased,^[^ ^9^, ^10^ ^]^ driven by improved analytical capabilities and the recognition of interesting bioactivities, but the discovery of their molecular targets is hampered by the often moderate‐low bioactivities and target affinities, that reflect the small size of volatiles. In this study, the mammalian target PDIA1 was inhibited with an in vitro potency that is in the same range as its phenotypic activity. Despite the small size and the reactive warhead, salinilactones exhibit a remarkable target selectivity for PDIs and the related thioredoxin (TRX1). The far more abundant (1*R*,5*S*)‐enantiomer **9** had a significantly lower binding affinity than the minor (1*S*,5*R*)‐enantiomer **8** against mammalian PDIA1. Inhibition of serine hydrolases, as observed for the larger, biosynthetically related cyclipostins/cyclophostin, was not observed, even though these also possess a configuration equivalent to the (1*S*,5*R*) configuration. An open question concerns the function of salinilactones in their natural environment. Bacteria lack PDI, but a directed search for homologs with a CXXC motif, or ABPP experiments with marine extracts might disclose further, environmental targets of salinilactones. Our results suggest that the search for molecular targets of volatile microbial products deserves to be expanded.

## 4 Statistical Analysis

Statistical analysis of the results of the activity‐based protein profiling, applying the SP3 protocol, was performed as follows. Identified proteins and their respective abundances have been exported from the Peaks Studio X pro software as a .csv file format and transformed to a .txt file format via Excel 2016. With this file, a generic matrix upload was done in Perseus 1.6.15.0. A categorical annotation was done assigning the ten samples (n = 10) to four DMSO negative controls (n = 4) and six positive controls containing the activity‐based probe **1** (n = 6). The data were then log_2_ transformed and missing values were replaced with the constant 0. A two‐tailed t‐test with a false discovery rate of FDR = 0.05 was carried out. The thereby calculated −log_10_(*P* values) were plotted in GraphPad Prism 8.4.3 against the calculated differences in abundance between the positive control (activity‐based probe) and the negative control (DMSO) as log_2_(positive control/negative control) that corresponds to log_2_(fold change).

## Conflict of Interest

The authors declare no conflict of interest.

## Supporting information

Supporting Information

## Data Availability

The data that support the findings of this study are available in the supplementary material of this article.
