# Supplementary material for: Activity‐Based Protein Profiling Identifies Protein Disulfide‐Isomerases as Target Proteins of the Volatile Salinilactones
Source: Adv Sci (Weinh). 2024 Mar 2;11(18):2309515. doi: 10.1002/advs.202309515 (PMC11095149; doi:10.1002/advs.202309515)
Supplement: Supplementary file 1 — Supporting Information [file ADVS-11-2309515-s001.pdf]

ADVANCED SCIENCE
Open Access

Supporting Information

for *Adv. Sci.*, DOI 10.1002/advs.202309515

Activity-Based Protein Profiling Identifies Protein Disulfide-Isomerases as Target Proteins of the Volatile Salinilactones

*Karoline Jerye, Helko Lüken, Anika Steffen, Christian Schlawis, Lothar Jänsch, Stefan Schulz and Mark Brönstrup**

Activity-Based Protein Profiling Identifies Protein Disulfide-Isomerases as Target Proteins of the Volatile Salinilactones

Karoline Jerye,[a] *Helko Lüken*,[a] *Anika Steffen*, [b] *Christian Schlawis*,[c] *Lothar Jänsch*,[d] *Stefan Schulz*,[c] and *Mark Brönstrup**[a, e, f]

[a] K. Jerye, H. Lüken, Prof. Dr. M. Brönstrup
Department of Chemical Biology
Helmholtz Centre for Infection Research
Inhoffenstraße 7, 38124 Braunschweig, Germany
mark.broenstrup@helmholtz-hzi.de

[b] PD Dr. A. Steffen
Department of Cell Biology
Helmholtz Centre for Infection Research
Inhoffenstraße 7, 38124 Braunschweig, Germany

[c] Dr. C. Schlawis, Prof. Dr. S. Schulz
Institute of Organic Chemistry
Technische Universität Braunschweig
Hagenring 30, 38106 Braunschweig, Germany

[d] Prof. Dr. L. Jänsch
Department of Cellular Proteome Research
Helmholtz Centre for Infection Research
Inhoffenstraße 7, 38124 Braunschweig, Germany

[e] Prof. Dr. M. Brönstrup
Institute of Organic Chemistry and Centre of Biomolecular Drug Research (BMWZ)
Leibniz Universität Hannover
Schneiderberg 1B, 30167 Hannover, Germany

[f] Prof. Dr. M. Brönstrup
German Center for Infection Research
Site Hannover-Braunschweig
Inhoffenstraße 7, 38124 Braunschweig, Germany

Table of Contents

1. General Information 3
2. Activity-based protein profiling (ABPP)....5
3. Sequence Alignment of identified PDI isoforms and Thioredoxin....14
4. Insulin reduction assay....16
5. Inhibition of Aldehyde Dehydrogenase....21
6. Intact protein ESI measurement....22
7. Binding site identification....24
8. Fluorophore labeling experiments....34
9. Synthetic procedures and compound characterization....38
10. NMR spectra 52
11. Structure elucidation of the ring opening reaction 66
12. References....70

1. General Information

Cell counting

The cell counting was done with a Cedex® XS Analyzer (Roche). Therefore, cell suspension solution was mixed with the same amount of Cedex Trypan blue solution (Roche Diagnostics, 05650640001). The mixtures was pipetted onto cell counting slides (ibidi, 80816) and cell counting was carried out with triplicates.

Dimethyl sulfoxide (DMSO)

For all biological experiments as well as for the stock solutions, DMSO for cell culture was used (PanReac AppliChem, A3672).

Ultra Pure Water

Ultra pure water was obtained from a PURELAB® flex 1 system from the company ELGA.

NMR Spectroscopy

NMR spectra were measured with an Avance III HD (700 MHz) and an Avance III (500 MHz) spectrometer from the company Bruker. Samples were dissolved in deuterated solvents from the company Sigma-Aldrich. NMR spectra were calibrated to the solvent peak.

High-Performance Liquid Chromatography (HPLC)

HPLC was carried out with a Dionex UltiMate 3000 system from Thermo Scientific. Separation was achieved using the following column: Luna® 5 µm C18(2) 100 Å, LC Column 250 x 21.2 mm, AXIA™ Packed (phenomenex, 00G-4252-P0-AX). As an eluent water (HPLC Gradient Grade, Baker HPLC Analyzed) and acetonitrile (Ultra Gradient HPLC Grade, Baker HPLC Analyzed) was used without further additives.

Column Chromatography

For reaction control and determination of a suitable eluent mixture, thin layer chromatography (TLC) plates with silica 60 coated on aluminum with the fluorescent indicator F254 were used (Merck, 1.05554.0001). Staining of the TLC plates was carried out with a potassium permanganate solution. Columns were packed with silica gel (pore size 60 Å, particle size 230-400 mesh, particle size 40-63 µm, Merck, 717185). Solvents were used from the supplier without further purification having the following purities: Baker Analyzed, ACS Grade, Baker Ultra Resi-Analyzed.

Dry Solvents

For water sensitive reactions, dry solvents were used from Thermo Scientific with an AcroSeal® cap.

Starting materials

Starting material were purchased from commercial suppliers (Sigma-Aldrich, TCI, BLDpharm, abcr, Carbolution, Thermo Scientific, Alfa Aesar, Acros Organics) and used without further purification.

Specific Rotation

Specific rotation was measured with a MCP 150 polarimeter in a quartz cuvette from the company Anton Paar. The value given is the average from three measurements. The specific rotation was calculated after: (mean of the measured value)/(g/mL).

PDIA1 Expression and Preparation

PDIA1 expression was done from a synthetic construct (pet28a-Strep-TEV-PDIA1, GenScript) in *E. coli* Bl21 (DE3) star. 2 × 1 L ZYM-5052 autoinducing medium were inoculated with 15 mL of an over night culture (LB-medium). Expression was carried out in baffled flasks in a shaking incubator (180 rpm). The cultures were initially incubated for 2 h at 37 °C, followed by 20 h at 20 °C. Yield: 2 × ~6 g pellet (wet weight), frozen in liquid nitrogen and stored at −80 °C.

Both pellets were processed the same way, but one without reductant in the buffer, the other one with 20 mM DTT and 4 mM TCEP. Buffer: 20 mM HEPES/NaOH, 300 mM NaCl, (reducing only: 20 mM DTT, 4 mM TCEP), pH 7.5. Pellets were resuspended in 40 mL buffer with one pill of cOmplete™, mini, EDTA-free protease inhibitor (Roche). Lysis was done by sonication (58% amplitude, 1 sec. pulse, 8 sec. pause, 35 min total). Centrifugation I: 30 min at 16.000 rpm in SA-600 rotor. Centrifugation II: supernatant from I for 1 h at 100.000 × g in Ti 30.50 rotor. The supernatant was loaded onto a 10 mL, self-packed Strep-Tactin high-capacity column (IBA) and eluted with a single step of 5 mM D-desthiobiotin dissolved in sample buffer. The protein was digested with TEV-protease (2:10) over night. Size-exclusion chromatography (SEC) was done with a HiLoad™ 16/600 Superdex™ 200 pg. Fractions were analyzed on an anykD™ SDS-PAGE (BioRad) and concentrated fractions were frozen in liquid nitrogen in 50 µL aliquots and stored at −80 °C.

Proteomics data availability

The mass spectrometry proteomics data have been deposited to the ProteomeXchange Consortium (http://proteomecentral.proteomexchange.org) via the PRIDE partner repository.[1]

The dataset identifier is PXD047342.

Username: reviewer_pxd047342@ebi.ac.uk

Password: 2nRLiLlL

2. Activity-based protein profiling (ABPP)

In gel digestion

1. Seeding 1.5 · 10^6 A549 cells in T25 cell culture flasks in a total volume of 5 mL media (gibco DMEM with GlutaMAX + 10% gibco FBS)
2. Incubation for 23 h at 37 °C and 5% CO_2
3. Removing media and washing cells with PBS (2 × 5 mL)
4. Detaching cells with Accutase® (BioLegend) (0.5 mL) for 3 min at 37 °C
5. Addition of media (4.5 mL)
6. Transfer of the cells to separate Falcon® tubes
7. Centrifugation of the tubes (5 min, 1000 rpm)
8. Removing media and washing cell pellet with PBS (1 × 5 mL)
9. Centrifugation (5 min, 1000 rpm)
10. Removal of PBS
11. Suspension of the cell pellet in 0.1% Triton™ X-100 (Sigma-Aldrich T8787) (100 µL) in ultra pure water and protease inhibitor (Pierce™ A32963) (20 µL) in ultra pure water
12. Transfer of each suspension to 1.5 mL protein LoBind® tubes (Eppendorf)
13. Cell lysis for 20 min on ice with vortexing every 10 min
14. Addition of the biotin salinilactone conjugate **1** (5 µL from 2 mM stock solution) to the cell lysate (final concentration 80 µM) or DMSO (5 µL) (PanReac AppliChem, A3672) as a negative control
15. Incubation for 5 h at 25 °C and 300 rpm in an Eppendorf ThermoMixer C.
16. Preparation of Streptavidin Sepharose™ beads (cytiva, 17511301)
    a. Resuspension of the beads thoroughly by panning the vial
    b. Transfer of the bead suspension (50 µL) to different 1.5 mL protein LoBind® tubes (Eppendorf) (for every sample one tube)
    c. Centrifugation (2 min, 2000 rpm)
    d. Removing supernatant
    e. Resuspension of the beads in 0.4% SDS solution in PBS (1 mL)
    f. Centrifugation (2 min, 2000 rpm)
    g. Removal of the supernatant
    h. Repeating the washing step three times
17. Addition of the cell lysates to the tubes with the beads
18. Addition of a 0.4% SDS solution in PBS (400 µL)
19. Vortexing the tubes
20. Incubated for 1 h in a rotary mixer at RT
21. Centrifugation (2 min, 4000 rpm)
22. Removing supernatants
23. Washing beads by resuspension, centrifugation (2 min, 4000 rpm) and removal of the supernatant with the following solutions:
    a. 3 × 0.4% SDS solution in PBS (1 mL)
    b. 2 × 3 M urea in ultra pure water (1 mL)
    c. 2 × PBS (1 mL)
24. Resuspension of the beads in a 3X Laemmli sample buffer (25 µL)
25. Heating for 6 min at 96 °C in a Eppendorf ThermoMixer C
26. Centrifugation of the tubes (2 min, 13000 rpm)

27. Transfer of the supernatents to Pierce™ Spin Columns (Thermo Scientific, 69725), placed in new protein LoBind® tubes (Eppendorf)
28. Centrifugation for few seconds
29. Preparation of a 1 mm thick SDS gel with 10% polyacrylamide in the resolving gel and 5% polyacrylamide in the stacking gel
    → Addition of 1 vol.-% of 2,2,2-trichloroethanol (Sigma-Aldrich, T54801) to the resolving gel for protein visualization
30. Recording the gel (Figure 2B, manuscript) with a Chemidoc system from Bio-Rad
31. Washing the gel with ultra pure water before the in gel digestion
32. Addition of a fixation solution consisting of ultra pure water (60 mL), ethanol (30 mL) and acetic acid (10 mL)
33. Incubation for 8 h at RT
34. Removing the fixation solution and washing the gel three times with ultra pure water
35. Addition of Coomassie by Kang[2] (50 mL) for protein staining over night at RT
36. Washing the gel three times with ultra pure water
37. Excising protein bands of interest with a scalpel on a glass plate, cut into 3-4 pieces and transfer to 1.5 mL protein LoBind® tubes (Eppendorf)
38. Addition of the destaining solution to the tubes consisting of 30% MeCN and 50 mM NH_4HCO_3 (400 µL)
39. Incubation for 5 h at RT until the gel bands are colorless (replacing destaining solution two times)
40. Removing the destaining solution
41. Washing gel pieces 5 min with water (400 µL)
42. Discarding supernatants
43. Dehydration with MeCN (300 µL) until gel pieces are white
44. Discarding supernatants
45. Reduction with 20 mM DTT in 0.1 M NH_4HCO_3 (40 µL) for 60 min at 56 °C
46. Discarding supernatants
47. Dehydration (see 43.)
48. Rehydration with 55 mM iodoacetamide in 0.1 M NH_4HCO_3 (40 µL) for 30 min in the dark at RT
49. Discarding supernatants
50. Dehydration (see 43.)
51. Rehydration with 0.1 M NH_4HCO_3 (250 µL) for 15 min at RT
52. Discarding supernatants
53. Dehydration (see 43.)
54. Drying the gel pieces in an eppendorf concentrator 5301 for 1 h at RT
55. Dissolving 20 µg sequence grade modified Trypsin by Promega (V5111) in resuspension buffer (from supplier) (100 µL), incubation for 30 min at RT, storage of 5 µL aliquots at −20 °C
56. Addition of Trypsin (0.2 µg/mL, 5 µL) to ultra pure water (250 µL) and 0.1 M NH_4HCO_3 (250 µL), vortex to obtain digestion solution
57. Addition of digestion solution to the tubes (60 µL) until gel pieces are covered
58. Incubation over night at 37 °C
59. Addition of MeCN (60 µL)
60. Incubation for 30 min at 37 °C and 600 rpm in an Eppendorf ThermoMixer C
61. Transfer of the supernatants into new 1.5 mL protein LoBind® tubes (Eppendorf), put aside

62. Addition of 5% formic acid (60 µL) to the gel pieces
63. Incubation for 30 min at 37 °C and 600 rpm in an Eppendorf ThermoMixer C
64. Addition of MeCN (60 µL)
65. Incubation for 30 min at 37 °C and 600 rpm in an Eppendorf ThermoMixer C
66. Transfer of the supernatants to the previous ones from step 61.
67. Removing volatiles at a CentriVap Concentrator (Labconco) overnight at RT

For MS/MS analyses, the peptides were loaded onto Evotips as described by the supplier Evosep:

1. Wash dry Evotips with 20 µL solvent B and centrifuge at 800 g for 60 s
2. Soak in 100 µL 2-propanol until all the tips are pale white
3. Equilibrate soaked Evotips with 20 µL solvent A and centrifuge at 800 g for 60 s
4. Load samples on wet tips (20 µL in solvent A) and centrifuge for 60 s at 800 g
5. Wash tips with 20 µL solvent A and centrifuge for 60 s at 800 g
6. Transfer 100 µL solvent A and centrifuge tips for 10 s at 800 g to keep tips wet

The peptides were eluted afterwards over an Evosep One HPLC connected to a timsTOF Pro from Bruker. Identification of the enriched peptides was done using the Peaks Studio 10.6 software with the following settings.

Search parameters:

Parent Mass Error Tolerance: 20.0 ppm

Fragment Mass Error Tolerance: 0.03 Da

Precursor Mass Search Type: monoisotopic

Enzyme: Trypsin

Max Missed Cleavages: 1

Digest Mode: Specific

Fixed Modifications: Carbamidomethylation: 57.02

Variable Modifications: Oxidation: 15.99

Max Variable PTM Per Peptide: 3

Database: UP_Homo-sapien_2020-12-22

Taxon: All

Searched Entry: 75773

FDR Estimation: Enabled

Merge Options: merged

Precursor Options: corrected

Charge Options: [2 – 5]

Filter RT: 0.0 – 21.0

Filter Charge: 2 – 5

Process: true

Associate chimera: yes

Instrument parameters: Ion source: ESI(nano-spray)

Fragmentation Mode: CID, CAD(y and b ions)

MS Scan Mode: TimsTOF

MS/MS Scan Mode: TimsTOF

Protein abundance was examined by label free quantification (LFQ) by adding up the areas under the curve of each feature (specific *m/z*, RT, charge, −10lg*P*); this was done for all peptides belonging to a given protein.

The results of the MS/MS analysis are shown in Figure S1.

			Duplicates Gel-Band 1		Duplicates Gel-Band 2		Duplicates Gel-Band 3			
	Accession	-10lgP	Coverage Sample 1	Coverage Sample 10	Coverage Sample 2	Coverage Sample 3	Coverage Sample 4	Coverage Sample 5	Avg. Mass	#Unique
	Proteins									
1	P07237|PDIA1_HUMAN	618.24	65%	48%	16%	0%	0%	7%	57116	44
2	P30101|PDIA3_HUMAN	538.85	51%	25%	28%	7%	0%	0%	56782	26
3	tr|H7BZJ3|H7BZJ3_HUMAN	318.54	48%	19%	28%	11%	0%	0%	13519	1
4	Q15084|PDIA6_HUMAN	532.47	0%	0%	0%	0%	44%	50%	48121	25
5	P68371|TBB4B_HUMAN	423.87	0%	0%	11%	0%	33%	37%	49831	2
6	P00352|AL1A1_HUMAN	357.89	0%	0%	34%	7%	12%	26%	54862	23
7	Q8NBS9|TXND5_HUMAN	352.55	0%	0%	0%	0%	19%	22%	47629	14

Figure S1. Results from the MS/MS analysis after in-gel digestion of three clearly enriched protein bands. Gel-band 1 is very likely identified as PDIA1 and PDIA3, with H7BZJ3 being a PDIA3 fragment. Gel-Band 2 contains some amounts of PDIA1 and PDIA3, but also shows the presence of AL1A1. Gel-Band 3 contains PDIA6 and PDIA15 (TXND5) and also AL1A1.Furthermore, it contains the protein TBB4B.

The MS/MS analysis of the three gel bands clearly shows the enrichment of different PDI isoforms. The decreasing masses of the proteins correlate to the gel band 1, 2 and 3. The occurrence of PDIA1 and PDIA3 in gel-band 2 could be the result of the limited precision of the gel excision (indicated by the dotted frame in Figure S1). The same applies for the protein AL1A1 in gel band 3.

Single-pot, solid-phase-enhanced sample preparation for proteomics experiments (SP3) protocol

1. Transfer of 2.5 $\cdot 10^6$ A549 cells suspended in media (gibco DMEM with GlutaMAX + 10% gibco FBS) (865 µL) to 1.5 mL protein LoBind® tubes (Eppendorf)
2. Centrifugation (5 min, 1000 rpm)
3. Removing media
4. Resuspension of the cell pellets in PBS (1 mL)
5. Centrifugation (5 min, 1000 rpm)
6. Removing supernatants
7. Suspension of the cell pellets in 0.1% Triton™ X-100 (Sigma-Aldrich T8787) (80 µL) in ultra pure water and protease inhibitor (Pierce™ A32963) (20 µL) in ultra pure water
8. Cell lysis for 20 min on ice with vortexing every 10 min
9. Addition of the biotin salinilactone conjugate **1** (5 µL from 2 mM stock solution) to the cell lysates (final concentration 95 µM) or DMSO (5 µL) (PanReac AppliChem, A3672) as a negative control
10. Incubation of the tubes for 5.5 h at 25 °C and 300 rpm in an Eppendorf ThermoMixer C
11. Preparation of the Streptavidin Sepharose™ beads (cytiva, 17511301)
    a. Resuspension of the beads thoroughly by panning the vial
    b. Transfer of the bead suspension (50 µL) to different 1.5 mL protein LoBind® tubes (Eppendorf) (for every sample one tube)
    c. Centrifugation of the tubes (2 min, 2000 rpm)
    d. Removing supernatants
    e. Resuspension of the beads in 0.4% SDS solution in PBS (1 mL)
    f. Centrifugation (2 min, 2000 rpm)
    g. Removal of the supernatants
    h. Repeating this washing step three times
12. Transfer of each cell lysate to different tubes containing the streptavidin beads
13. Addition of a 0.4% SDS solution in PBS (400 µL)
14. Vortexing the samples and incubation for 1 h at RT in a rotary mixer
15. Centrifugation (2 min, 2000 rpm)
16. Removing the supernatants
17. Resuspension of the beads, centrifugation (2 min, 2000 rpm) and removing supernatants in each of the following washing steps:
    a. 3 × 0.4% SDS solution in PBS (1 ml)
    b. 2 × 3 M urea in ultra pure water (1 mL)
    c. 2 × PBS (1 mL)
18. Addition of a 2% SDS solution in ultra pure water (50 µL) to the tubes
19. Heating the samples for 6 min at 96 °C in an Eppendorf ThermoMixer C
20. Centrifugation (2 min, 13000 rpm)
21. Transfer of the supernatants to Pierce™ Spin Columns (Thermo Scientific, 69725), placed in new protein LoBind® tubes (Eppendorf)
22. Centrifugation for few seconds
23. Preparing the SpeedBeads magnetic carboxylate modified particles (GE45152105050250)
    a. Resuspension thoroughly on an orbital shaker for half a day
    b. Transfer 4 mL of the suspension to a 5 mL protein LoBind® tube (Eppendorf) and let beads sediment on a magnet

   c. Remove the glycerin storage solution and wash three times with ultra pure water
   d. Transfer 30 µL by height to a 1.5 mL protein LoBind® tubes (Eppendorf) and add 1 mL ultra pure water
   e. Store in the fridge at 4 °C

24. Reduction with 50 mM TCEP in ultra pure water (10 vol.-%, 5 µL) for 1 h at 55 °C and 700 rpm in an Eppendorf ThermoMixer C
25. Cooling to RT for 15 min
26. Alkylation with 200 mM MMTS in *iso*-PrOH (5 vol.-%, 2.5 µL) for 10 min at RT and 700 rpm in an Eppendorf ThermoMixer C
27. Resuspension of the magnetic beads from step 23. by vortexing
28. Addition of magnetic bead solution (20 µL) to every sample, followed by MeCN (20 µL)
29. Incubation over night at 22 °C and 1000 rpm in an Eppendorf ThermoMixer C
30. Centrifugation (5 min, 5000 rpm)
31. Place a magnet at the side of the tube and transfer the supernatant into new 1.5 mL protein LoBind® tubes (Eppendorf)
32. Addition of magnetic bead suspension (10 µL) to the supernatants
33. Incubation for 7 h at 22 °C and 1000 rpm in an Eppendorf ThermoMixer C
34. Resuspension of the beads from step 31. in EtOH (300 µL)
35. Incubated for 7 h at 22 °C and 1000 rpm in an Eppendorf ThermoMixer C
36. Centrifugation (5 min, 5000 rpm) of all tubes
37. Tubes with 20 µL beads: discarding supernatants
38. Tubes with 10 µL beads: discarding supernatants, resuspension in EtOH (300 µL) and addition to the tubes with the 20 µL beads
39. Incubation for 15 min at 22 °C and 1000 rpm in an Eppendorf ThermoMixer C
40. Centrifugation (5 min, 5000 rpm)
41. Discarding supernatants, resuspension in EtOH (300 µL)
42. Incubation for 15 min at 22 °C and 1000 rpm in an Eppendorf ThermoMixer C
43. Centrifugation (5 min, 5000 rpm)
44. Discarding supernatants, resuspension in MeCN (300 µL), incubation for 15 min at 22 °C and 1000 rpm in an Eppendorf ThermoMixer C
45. Centrifugation (5 min, 5000 rpm)
46. Discarding supernatants and drying samples for 15 min with open lids in the fume hood
47. Resuspension in 1 M TEAB buffer (50 µL) pH 8.5
48. Addition of Trypsin by Promega (V5111) (0.2 µg/mL, 2 µL) (preparation see in gel digestion step 55.)
49. Incubation over night at 37 °C and 1000 rpm in an Eppendorf ThermoMixer C
50. Resuspension in MeCN (1 mL) and addition of magnetic bead solution (10 µL)
51. Incubation over night at 22 °C and 1000 rpm in an Eppendorf ThermoMixer C
52. Centrifugation (5 min, 5000 rpm)
53. Discarding supernatants and resuspension in MeCN (200 µL)
54. Centrifugation (5 min, 5000 rpm)
55. Discarding supernatants, resuspension in MeCN (200 µL) and incubation for 15 min at 22 °C and 1000 rpm in an Eppendorf ThermoMixer C
56. Centrifugation (5 min, 5000 rpm)
57. Discarding supernatants and drying tubes with an open lid in the fume hood for 25 min
58. Peptide elution

a. Addition of 2% DMSO in ultra pure water (20 µL) and resuspension of the beads
b. Incubation for 15 min at 22 °C and 1000 rpm in an Eppendorf ThermoMixer C
c. Centrifugation (5 min, 5000 rpm)
d. Transfer of the supernatants to new 1.5 mL protein LoBind® tubes (Eppendorf)
e. Resuspension of the beads in ultra pure water (20 µL)
f. Incubation for 15 min at 22 °C and 1000 rpm in an Eppendorf ThermoMixer C
g. Centrifugation (5 min, 5000 rpm)
h. Transfer of the supernatants to the tubes from step 58.d.
i. Removing volatiles at a CentriVap Concentrator (Labconco) overnight at 23 °C

For MS/MS analyses, the peptides were loaded onto Evotips as described by the supplier Evosep:

1. Wash dry Evotips with 20 µL solvent B and centrifuge at 800 g for 60 s
2. Soak in 100 µL 2-propanol until all the tips are pale white
3. Equilibrate soaked Evotips with 20 µL solvent A and centrifuge at 800 g for 60 s
4. Load samples on wet tips (20 µL in solvent A) and centrifuge for 60 s at 800 g
5. Wash tips with 20 µL solvent A and centrifuge for 60 s at 800 g
6. Transfer 100 µL solvent A and centrifuge tips for 10 s at 800 g to keep tips wet

The peptides were eluted afterwards over an Evosep One HPLC connected to a timsTOF Pro from Bruker. Identification of the enriched peptides was done using the Peaks Studio 10.6 software with the following settings.

Search parameters:

Parent Mass Error Tolerance: 20.0 ppm

Fragment Mass Error Tolerance: 0.03 Da

Precursor Mass Search Type: monoisotopic

Enzyme: Trypsin

Max Missed Cleavages: 1

Digest Mode: Specific

Fixed Modifications: Beta-methylthiolation: 45.99

Variable Modifications: Oxidation: 15.99

Max Variable PTM Per Peptide: 3

Database: UP_Homo-sapien_2020-12-22

Taxon: All

Searched Entry: 75773

FDR Estimation: Enabled

Merge Options: merged

Precursor Options: corrected

Charge Options: [2 – 5]

Filter RT: 3.0 – 21.0

Filter Charge: 2 – 5

Process: true

Associate chimera: yes

Instrument parameters: Ion source: ESI(nano-spray)

Fragmentation Mode: CID, CAD (y and b ions)

MS Scan Mode: TimsTOF

MS/MS Scan Mode: TimsTOF

Protein abundance was examined by label free quantification (LFQ) by adding up the areas under the curve of each feature (specific *m/z*, RT, charge, −10lg*P*); this was done for all peptides belonging to a given protein. The results of the MS/MS analysis are shown in figure S2. These data were used for the statistical analysis, and the results are depicted in a volcano plot in the manuscript in figure 2D. Further information about the statistical analysis is given at the end of the manuscript.

				6 samples with biotin salinilactone conjugate 1						4 samples with DMSO vehicle control					
	Accession	-10lgP	Coverage	Covera...	Coverage ...	Coverage ...	Coverage ...	Coverage ...	Coverage ...	Coverage ...	Coverage ...	Coverage ...	Coverage ...	Avg. Mass	#Unique
	Proteins														
1	P07237|PDIA1_HUMAN	607.89	70%	67%	65%	63%	65%	63%	70%	0%	0%	0%	0%	57116	38
2	tr|A0A499FI48|A0A499FI48_HUMAN	591.88	59%	53%	46%	49%	48%	45%	53%	5%	2%	2%	2%	73061	1
3	P13667|PDIA4_HUMAN	627.31	59%	53%	46%	49%	48%	45%	53%	5%	2%	2%	2%	72933	2
4	P04264|K2C1_HUMAN	673.46	55%	44%	50%	55%	53%	44%	53%	46%	40%	47%	52%	66039	37
5	P62805|H4_HUMAN	291.99	55%	44%	44%	55%	40%	44%	55%	13%	20%	44%	44%	11367	7
6	P30101|PDIA3_HUMAN	495.55	51%	43%	44%	44%	41%	41%	43%	0%	5%	0%	0%	56782	14
7	O95994|AGR2_HUMAN	237.32	43%	42%	16%	20%	22%	17%	33%	0%	0%	0%	0%	19979	5
8	tr|H7BZJ3|H7BZJ3_HUMAN	348.86	60%	41%	33%	33%	60%	33%	41%	0%	0%	0%	0%	13519	1
9	P05165|PCCA_HUMAN	481.53	45%	41%	32%	28%	35%	27%	25%	28%	28%	28%	29%	80059	24
10	P11498|PYC_HUMAN	582.50	48%	40%	38%	39%	41%	38%	43%	34%	40%	37%	35%	129634	44
11	tr|E7EVS6|E7EVS6_HUMAN	352.13	49%	39%	32%	40%	39%	27%	20%	19%	27%	15%	27%	36940	4
12	P35527|K1C9_HUMAN	605.39	60%	38%	48%	56%	55%	47%	57%	47%	41%	52%	48%	62064	31
13	tr|A0A0G2JLD8|A0A0G2JLD8_HUMAN	258.06	38%	38%	23%	38%	23%	23%	38%	17%	17%	17%	6%	15598	4
14	tr|J3QSA3|J3QSA3_HUMAN	120.86	37%	37%	37%	37%	37%	37%	37%	0%	0%	37%	0%	4854	1
15	P13645|K1C10_HUMAN	613.37	55%	36%	30%	49%	47%	43%	54%	28%	21%	40%	46%	58827	29
16	Q15084|PDIA6_HUMAN	460.12	52%	36%	40%	43%	36%	38%	45%	3%	5%	0%	3%	48121	18
17	tr|E9PHF7|E9PHF7_HUMAN	449.38	42%	33%	28%	31%	36%	22%	24%	22%	31%	28%	28%	68332	17
18	P07437|TBB5_HUMAN	291.47	30%	30%	23%	27%	23%	19%	7%	9%	9%	3%	17%	49671	3
19	P35908|K22E_HUMAN	537.46	72%	28%	33%	61%	54%	31%	51%	39%	26%	36%	42%	65433	25
20	O95881|TXD12_HUMAN	251.54	38%	25%	29%	34%	29%	29%	34%	0%	0%	0%	0%	19206	7
21	Q8NBS9|TXND5_HUMAN	285.29	29%	23%	23%	23%	18%	21%	18%	4%	0%	0%	4%	47629	11
22	P10599|THIO_HUMAN	165.47	23%	23%	12%	23%	12%	12%	12%	0%	0%	0%	0%	11737	2
23	Q13085|ACACA_HUMAN	558.17	30%	21%	15%	19%	18%	13%	12%	21%	21%	16%	19%	265551	53
24	P68371|TBB4B_HUMAN	291.90	21%	21%	17%	21%	17%	13%	7%	9%	9%	3%	17%	49831	1
25	P00352|AL1A1_HUMAN	275.93	24%	18%	23%	18%	18%	18%	18%	0%	3%	0%	3%	54862	9
26	tr|F5H5D3|F5H5D3_HUMAN	256.39	17%	17%	12%	14%	14%	12%	12%	11%	11%	9%	11%	57730	6
27	P68032|ACTC_HUMAN	330.15	27%	16%	20%	21%	20%	20%	17%	6%	13%	13%	20%	42019	1
28	P68104|EF1A1_HUMAN	217.66	21%	16%	5%	7%	9%	2%	10%	0%	5%	5%	7%	50141	5
29	tr|H0YM50|H0YM50_HUMAN	175.91	22%	15%	7%	14%	14%	0%	7%	0%	8%	0%	0%	28379	3
30	P29401|TKT_HUMAN	276.10	13%	13%	13%	10%	13%	13%	7%	8%	2%	8%	13%	67878	5
31	tr|E9PJD9|E9PJD9_HUMAN	90.30	12%	12%	0%	0%	0%	12%	12%	0%	0%	12%	0%	10127	1
32	tr|A0A2R8Y7C0|A0A2R8Y7C0_HUMAN	209.07	30%	12%	11%	23%	30%	23%	12%	0%	23%	0%	0%	13962	3
33	P49411|EFTU_HUMAN	289.36	29%	12%	15%	6%	12%	7%	9%	9%	21%	14%	14%	49542	9
34	tr|A0A3B3ITT5|A0A3B3ITT5_HUMAN	124.17	16%	9%	7%	9%	0%	16%	9%	0%	0%	0%	16%	18512	2
35	P08779|K1C16_HUMAN	315.19	40%	8%	4%	37%	18%	6%	15%	2%	0%	8%	9%	51268	8
36	tr|E7EQ64|E7EQ64_HUMAN	73.21	8%	8%	8%	8%	8%	8%	8%	8%	8%	8%	8%	28123	1
37	O00299|CLIC1_HUMAN	87.74	7%	7%	0%	0%	7%	0%	0%	0%	0%	0%	0%	26923	1
38	P62937|PPIA_HUMAN	125.73	36%	7%	16%	9%	17%	9%	9%	0%	12%	0%	0%	18012	4
39	Q562R1|ACTBL_HUMAN	212.38	19%	7%	12%	19%	12%	7%	7%	0%	10%	8%	12%	42003	2
40	P08238|HS90B_HUMAN	175.17	14%	7%	5%	9%	8%	7%	3%	0%	0%	0%	0%	83264	7
41	tr|E7EX53|E7EX53_HUMAN	77.46	7%	7%	7%	7%	7%	7%	7%	0%	7%	7%	7%	15722	1
42	P02533|K1C14_HUMAN	366.67	36%	7%	10%	29%	23%	5%	32%	7%	0%	3%	21%	51562	8
43	P62241|RS8_HUMAN	176.64	13%	6%	0%	6%	0%	7%	6%	0%	0%	0%	7%	24205	2
44	tr|E9PNE6|E9PNE6_HUMAN	100.84	23%	5%	12%	6%	5%	6%	5%	4%	5%	0%	0%	54976	2
45	Q9Y6N5|SQOR_HUMAN	103.02	8%	5%	8%	0%	5%	0%	5%	5%	5%	5%	5%	49961	2
46	Q13263|TIF1B_HUMAN	71.79	8%	4%	0%	4%	4%	0%	4%	4%	0%	0%	0%	88550	2
47	O43790|KRT86_HUMAN	89.27	12%	4%	0%	4%	8%	3%	0%	0%	3%	4%	0%	53501	3
48	Q9H3N1|TMX1_HUMAN	165.03	11%	4%	11%	11%	4%	11%	4%	0%	0%	0%	0%	31791	2
49	Q5XKE5|K2C79_HUMAN	261.98	27%	4%	4%	15%	6%	2%	8%	0%	0%	13%	6%	57836	4
50	P19013|K2C4_HUMAN	159.19	8%	4%	7%	4%	4%	8%	4%	4%	4%	4%	4%	56144	2
51	P04259|K2C6B_HUMAN	406.55	29%	3%	3%	29%	21%	3%	17%	5%	3%	3%	12%	60067	1
52	P04406|G3P_HUMAN	79.66	11%	3%	0%	7%	3%	0%	7%	0%	3%	0%	3%	36053	3
53	tr|A0A1C7CYW7|A0A1C7CYW7_HU...	79.81	3%	3%	1%	1%	1%	1%	1%	1%	3%	1%	1%	115707	2
54	P11413|G6PD_HUMAN	125.73	11%	3%	3%	3%	3%	4%	0%	4%	0%	0%	0%	59257	3
55	P13639|EF2_HUMAN	68.40	7%	2%	0%	0%	0%	0%	0%	0%	1%	0%	4%	95338	4
56	P05787|K2C8_HUMAN	178.93	14%	2%	2%	6%	6%	4%	2%	2%	5%	2%	4%	53704	1
57	P48668|K2C6C_HUMAN	420.46	32%	2%	2%	32%	24%	2%	17%	4%	2%	2%	10%	60025	0
58	P02538|K2C6A_HUMAN	414.29	32%	2%	2%	32%	25%	2%	18%	4%	2%	2%	10%	60045	1
59	Q86YZ3|HORN_HUMAN	343.24	9%	1%	1%	5%	6%	1%	2%	3%	0%	2%	4%	282389	14
60	P21333|FLNA_HUMAN	145.94	4%	1%	1%	0%	2%	0%	0%	0%	1%	0%	1%	280737	5
61	Q9UQ35|SRRM2_HUMAN	189.67	8%	1%	2%	3%	2%	3%	1%	1%	2%	1%	3%	299616	15
62	Q9Y4F3|MARF1_HUMAN	67.30	3%	1%	1%	1%	1%	1%	3%	1%	1%	1%	1%	192858	3
63	P13647|K2C5_HUMAN	433.61	36%	0%	3%	36%	26%	3%	22%	7%	0%	6%	15%	62378	12
64	Q86Y46|K2C73_HUMAN	222.49	10%	0%	4%	6%	4%	0%	4%	4%	0%	0%	0%	58923	3
65	Q02413|DSG1_HUMAN	212.26	11%	0%	0%	2%	5%	0%	8%	2%	5%	0%	2%	113748	6
66	P15924|DESP_HUMAN	189.39	5%	0%	0%	2%	2%	1%	0%	0%	0%	1%	1%	331774	9
67	tr|A0A0A0MSI0|A0A0A0MSI0_HUMAN	188.50	25%	0%	0%	19%	18%	6%	12%	0%	0%	0%	0%	18976	4
68	tr|G8JLG2|G8JLG2_HUMAN	161.77	8%	0%	0%	8%	3%	0%	0%	0%	0%	0%	0%	51607	2
69	P14923|PLAK_HUMAN	147.83	7%	0%	0%	2%	2%	0%	0%	4%	0%	0%	0%	81745	2
70	P38919|IF4A3_HUMAN	119.45	18%	0%	0%	0%	7%	4%	0%	3%	0%	4%	4%	46871	4
71	P81605|DCD_HUMAN	107.98	13%	0%	0%	13%	13%	0%	13%	0%	0%	0%	0%	11284	1
72	tr|H0YHA7|H0YHA7_HUMAN	88.52	8%	0%	0%	0%	0%	0%	8%	0%	0%	0%	0%	18982	1
73	Q7L014|DDX46_HUMAN	84.08	6%	0%	0%	2%	0%	2%	0%	0%	2%	2%	0%	117362	3
74	tr|A0A2R8Y7R2|A0A2R8Y7R2_HUMAN	77.05	8%	0%	0%	0%	0%	8%	0%	0%	0%	0%	0%	12176	1

Figure S2. Results from the MS/MS analysis after the SP3 protocol. Six replicates with the biotin salinilactone derivative **1** and four replicates with DMSO as vehicle control were prepared. The orange boxes highlight ten specifically enriched PDI isoforms as well as THIO_human, which is structurally related to the PDIs.

3. Sequence Alignment of identified PDI isoforms and Thioredoxin

In the following, the amino acid sequence of the main target protein PDIA1 is shown. The catalytic active centers are marked in red.

```
>sp|P07237|PDIA1_HUMAN Protein disulfide-isomerase OS=Homo sapiens OX=9606 GN=P4HB
PE=1 SV=3

                                                          53  56
1   MLRRALLCLAVAALVRADAPEEEDHVLVLRKSNFAEALAAHKYLLVEFYAPWCGHCKALA
61  PEYAKAAGKLKAEGSEIRLAKVDATEESDLAQQYGVRGYPTIKFFRNGDTASPKEYTAGR
121 EADDIVNWLKKRTGPAATTLPDGAAAESLVESSEVAVIGFFKDVESDSAKQFLQAAEAID
181 DIPFGITSNSDVFSKYQLDKDGVVLFKKFDEGRNNFEGEVTKENLLDFIKHNQLPLVIEF
241 TEQTAPKIFGGEIKTHILLFLPKSVSDYDGKLSNFKTAAESFKGKILFIFIDSDHTDNQR
301 ILEFFGLKKEECPAVRLITLEEEMTKYKPESEELTAERITEFCHRFLEGKIKPHLMSQEL
                                                 397 400
361 PEDWDKQPVKVLVGKNFEDVAFDEKKNVFVEFYAPWCGHCKQLAPIWDKLGETYKDHENI
421 VIAKMDSTANEVEAVKVHSFPTLKFFPASADRTVIDYNGERTLDGFKKFLESGGQDGAGD
481 DDDLEDLEEAEEPDMEEDDDQKAVKDEL
```

Due to the identification of several PDI isoforms, we assumed that the binding of the salinilactone most likely occurs at a position, which is similar in all these proteins. The sequence alignment of the respective proteins (Figure S3) showed a high sequence conservation around the catalytic active sites.

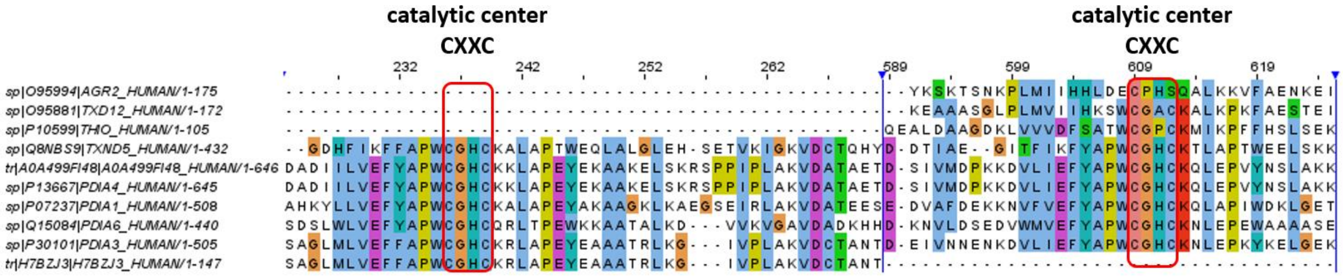

Figure S3. Sequence alignment of the enriched PDI isoforms and the structurally related protein thioredoxin in the regions covering the catalytic sites. The red boxes mark the catalytic active sites of the proteins. Alignment was carried out with Clustal Omega and visualized with Jalview 2.11.2.7. The color code is explained in Table S1.

Table S1. Legend of the Clustal color scheme.

Clustal X Default Coloring			
**Catagory**	**Colour**	**Residue at position**	**(Threshold, Residue group)**
Hydrophobic	BLUE	A,I,L,M,F,W,V	(>60%, WLVIMAFCHP)
		C	(>60%, WLVIMAFCHP)
Positive charge	RED	K,R	(>60%,KR),(>80%, K,R,Q)
Negative charge	MAGENTA	E	(>60%,KR),(>50%,QE),(>85%,E,Q,D)
		D	(>60%,KR), (>85%, K,R,Q), (>50%,ED)
Polar	GREEN	N	(>50%, N), (>85%, N,Y)
		Q	(>60%,KR),(>50%,QE),(>85%,Q,E,K,R)
		S,T	(>60%, WLVIMAFCHP), (>50%, TS}, (>85%,S,T)
Cysteines	PINK	C	(>85%, C)
Glycines	ORANGE	G	(>0%, G)
Prolines	YELLOW	P	(>0%, P)
Aromatic	CYAN	H,Y	(>60%, WLVIMAFCHP), (>85%, W,Y,A,C,P,Q,F,H,I,L,M,V)
Unconserved	WHITE	any / gap	If none of the above criteria are met

4. Insulin reduction assay

In the following, a detailed description of the insulin reduction assay as carried out by us is given.

1. Preparation of buffer A (100 mM NaH_2PO_4, pH 7.0)
2. Preparation of buffer B (100 mM NaH_2PO_4, 4 mM EDTA, pH 7.0)
3. Preparation of a 5.8 mM DTT solution in buffer A (V = 3 mL)
4. Dilution of inhibitor with buffer A to c = 4.5 mM (V = 200 µL) (4.5% DMSO)
5. Dilution of DMSO with buffer A to 4.5% DMSO
6. Dilution of rec. PDIA1 (0.8 mg/mL, in ultra pure water) (Novus Biologicals, NBP2-35195, *E. coli*) with DTT-buffer A from step 3. to a concentration of 180 µg/mL (V_{total} = 680 µL)
7. Dilution of DTT-buffer A from step 3. with the same amount of ultra pure water as in step 6. with PDIA1 solution
8. Addition of ultra pure water (90 µL) in all outer wells of a 96 half area plate (Grainer 675101) to reduce edge effects; outer wells are left out in the following steps
9. Addition of inhibitor-buffer A solution (30 µL) from step 4. to column 2 (triplicates)
10. Addition of DMSO-buffer A solution (20 µL) to column 3-11
11. Serial dilution by pipetting 10 µL from column 2, to column 3, pipetting up and down, and transferring again 10 µL to column 4 and so on until column 10, discard remaining 10 µL after column 10
12. Addition of DTT-buffer A solution (10 µL) from step 7. to the three background wells in column 11
13. Addition of PDI-DTT-buffer A solution (10 µL) from step 6. to all wells with inhibitor and vehicle control (usage of SurPhob tips from Biozym, VT0104)
14. Place a foil (HJ-BIOANALYTIK GmbH, 900510) on the top of the plate and close the lid to reduce evaporation during incubation
15. Incubation for 1 h or 6 h at 37 °C
    → concentrations: PDIA1 = 60 µg/mL, DTT = 1.5 mM, DMSO = 3%
16. Dilution of insulin stock solution (10 mg/mL, Sigma-Aldrich I0516) with buffer B to 1.125 mg/mL (V_{total} = 4.8 mL)
17. Cool down the 96 well plate after the incubation time to RT for 5 min, remove the foil
18. Start the catalytic reaction by addition of the insulin-buffer B solution (60 µL) from step 16. with a multichannel pipette to all wells (usage of SurPhob tips from Biozym, VT0144)
    → final concentrations: PDIA1 = 20 µg/mL, DTT = 500 µM, insulin = 130 µM, DMSO = 1%
19. Bursting any occurring bubble with a cannula
20. Measuring absorbance at λ = 650 nm with a Tecan-Reader Infinite M200 Pro without the lid every minute for 120 min
21. Before the first measurement, let shake for 10 s with a 4 mm amplitude, for every following measurement led the reader shake again for 3 s

A possible setup for the 96 half area plate looks like this:

96 wells	1	2	3	4	5	6	7	8	9	10	11	12
	H_2O	H_2O	H_2O	H_2O	H_2O	H_2O	H_2O	H_2O	H_2O	H_2O	H_2O	H_2O
Inhibitor A	H_2O	3000 µM	1000 µM	333.3 µM	111.1 µM	37.04 µM	12.35 µM	4.115 µM	1.372 µM	0.457 µM	Vehicle	H_2O
Inhibitor A	H_2O	3000 µM	1000 µM	333.3 µM	111.1 µM	37.04 µM	12.35 µM	4.115 µM	1.372 µM	0.457 µM	Vehicle	H_2O
Inhibitor A	H_2O	3000 µM	1000 µM	333.3 µM	111.1 µM	37.04 µM	12.35 µM	4.115 µM	1.372 µM	0.457 µM	Vehicle	H_2O
Inhibitor B	H_2O	3000 µM	1000 µM	333.3 µM	111.1 µM	37.04 µM	12.35 µM	4.115 µM	1.372 µM	0.457 µM	BG	H_2O
Inhibitor B	H_2O	3000 µM	1000 µM	333.3 µM	111.1 µM	37.04 µM	12.35 µM	4.115 µM	1.372 µM	0.457 µM	BG	H_2O
Inhibitor B	H_2O	3000 µM	1000 µM	333.3 µM	111.1 µM	37.04 µM	12.35 µM	4.115 µM	1.372 µM	0.457 µM	BG	H_2O
	H_2O	H_2O	H_2O	H_2O	H_2O	H_2O	H_2O	H_2O	H_2O	H_2O	H_2O	H_2O

For the analyses of the results, the mean value of the background wells was subtracted from all values of the inhibitor and vehicle wells. The remaining values were plotted in GraphPad Prism 8.4.3 with triplicates for each concentration against the measuring time. For the calculation of the IC_{50} values, measuring points from a region with a linear increasing optical density (usually between 40 min and 50 min) were plotted against the concentration. A nonlinear regression [Inhibitor] vs. response – variable slope (four parameters) yielded the IC_{50} values.

Inhibition of human TRX1 (*E. coli*)

Inhibition of human TRX1 was also tested in an insulin reduction described previously. Human thioredoxin was purchased from R&D Systems (1970-TX). TRX1 was used at a concentration of 20 µg/mL and incubated with the salinilactone B enantiomers.

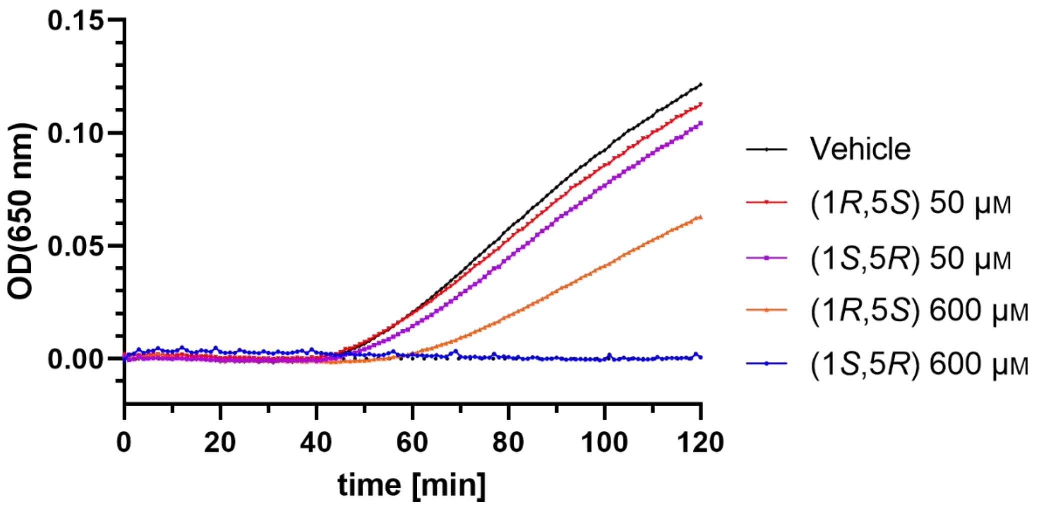

Figure S4. Time dependent turbidity plot of TRX1 inhibition by (1*R*,5*S*)- and (1*S*,5*R*)-salinilactone B after 6 h preincubation (n = 1).

Determination of k_{inact} and K_I

The determination of the rate of covalent modification was carried out by applying different pre-incubation times (1-6 h) of the (1*S*,5*R*)-enantiomer **8** with recombinant PDIA1, before starting the enzymatic reaction of insulin reduction. The assay was carried out according to the following protocol.

1. Preparation of buffer A (100 mM NaH_2PO_4, pH 7.0)
2. Preparation of buffer B (100 mM NaH_2PO_4, 4 mM EDTA, pH 7.0)
3. Preparation of TCEP buffer (20 mM Tris-HCl, 150 mM NaCl, 4 mM TCEP, pH 8.0)
4. Preparation of stock solutions of enantiomer **8** (1333.3 µM, 444.4 µM, 148.1 µM, 49.4 µM with V = 600 µL, DMSO = 4%) by serial dilution of a 100 mM stock solution in DMSO with buffer A (for equal DMSO concentration, following dilution steps were done with buffer A containing 4% DMSO)
5. Dissolving DTT in buffer A (c = 13.6 mM, V = 2 mL)
6. Dilution of rec. PDIA1 stock solution (1 mg/mL in 20 mM Tris-HCl, 150 mM NaCl, 4 mM TCEP, pH 8.0, produced in *E. coli* by Dr. P. Lukat) with buffer A-DTT from step 5 to 120 µg/mL (V = 700 µL, DTT: c = 12 mM)
7. Dilution of buffer A-DTT from step 5 with TCEP buffer from step 3 in the same ratio like step 6 (V = 700 µL, DTT: c = 12 mM)
8. Transfer of the stock solutions (30 µL) from step 4 to a 96 well plate (Greiner 655101) (for each stock solution 12 wells → six time points with duplicates)
9. Transfer of buffer A with 4% DMSO (30 µL) to six vehicle and six background wells
10. Transfer of buffer A-TCEP solution (10 µL) from step 7 to the background well of the first incubation time
11. Transfer of the diluted PDIA1 solution (10 µL) from step 6 to all inhibitor wells and one vehicle well of the first incubation time (usage of SurPhob tips from Biozym, VT0104)
12. Place a foil (HJ-BIOANALYTIK GmbH, 900510) on the top of the plate and close the lid to reduce evaporation during incubation
13. Incubation for 1 h at 37 °C
    → concentrations: PDIA1 = 30 µg/mL, DTT = 3 mM, DMSO = 3%
14. Removing the foil up to the wells for the next incubation time and repeat step 10 and step 11
15. Incubation for 1 h at 37 °C with closed foil and lid
16. Repetition of step 10-14 until all incubations times (1 h, 2 h, 3 h, 4 h, 5 h and 6 h) are carried out
17. Preparation of the insulin solution during the last incubation time
    → Dilution of insulin stock solution (10 mg/mL, Sigma-Aldrich I0516) with buffer B from step 2 to 1.125 mg/mL (V_{total} = 7 mL)
18. Cooling down the well plate to room temperature for 7 min after the last incubation time
19. Remove the foil and the lid
20. Start the catalytic reaction by addition of 80 µL of the diluted insulin solution from step 18 to all wells (usage of SurPhob tips from Biozym, VT0144)
    → final concentrations: PDIA1 = 10 µg/mL, DTT = 1 mM, insulin = 130 µM, DMSO = 1%
21. Measuring absorbance at λ = 650 nm with a Tecan-Reader Infinite M200 Pro without the lid every 70 s for 120 min

22. Before the first measurement, let shake for 10 s with a 1.5 mm amplitude. Afterwards, let the reader shake for 3 s with a 4 mm amplitude before every following measurement.

For the analyses of the results, the value of each background well was subtracted from the inhibitor and vehicle wells with the same incubation time. The resulting OD values of inhibitor and vehicle wells with the same preincubation time were plotted in GraphPad Prism 8.4.3 against the reaction time of the insulin reduction. The curves show mean values of the duplicates for each inhibitor concentration (Figure S5). The preincubation time-dependent inhibition was analyzed at the measuring time point of 39.7 min after insulin addition. Therefore, the value of the optical density of each concentration of all preincubation times were normalized by the enzyme activity of the vehicle sample at the same time point. The thereby calculated remaining enzyme activity (A_R) was plotted against the inhibitor concentration for every preincubation time (Figure S6).

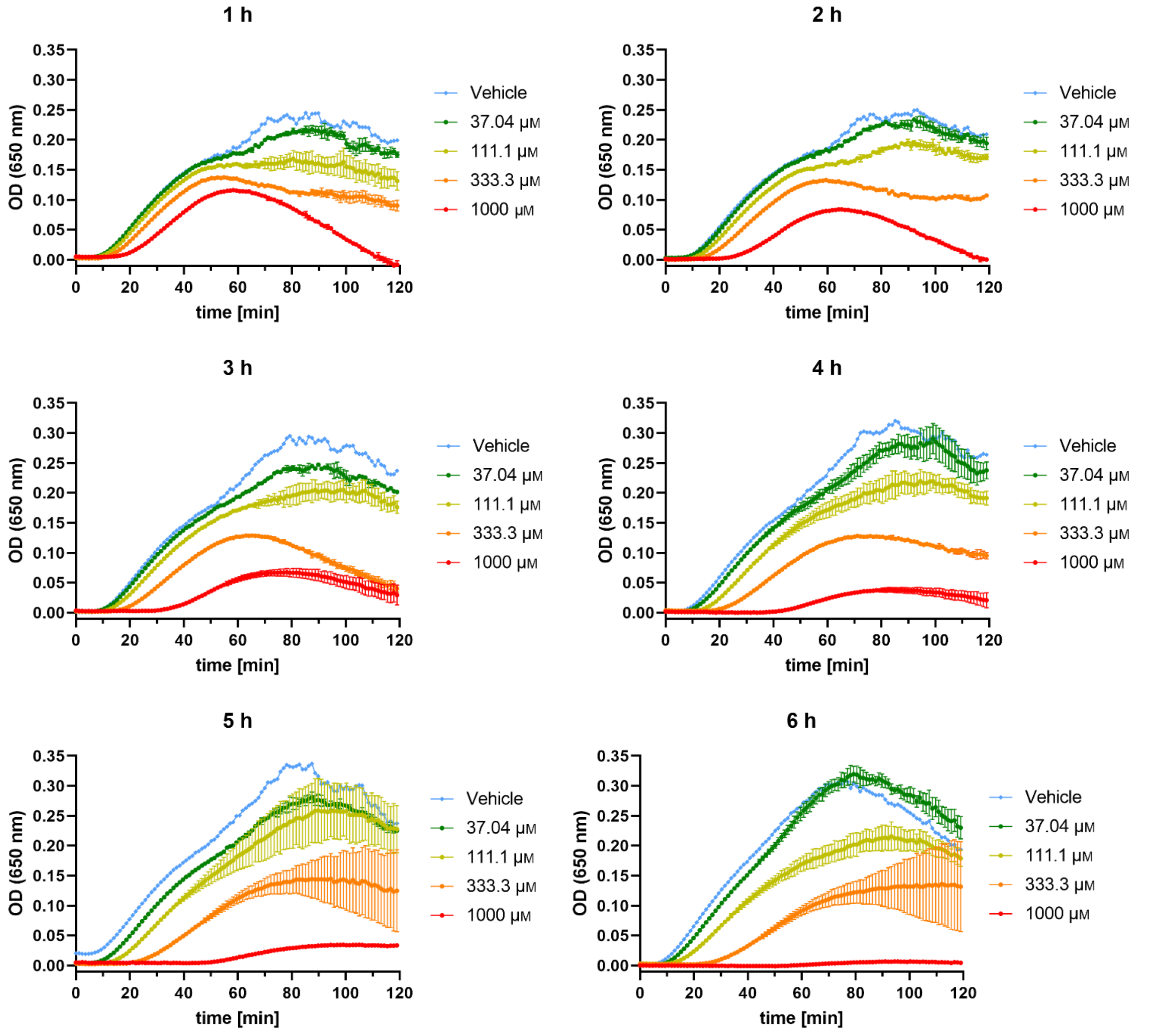

Figure S5. Time dependent turbidity plots of PDIA1 inhibition by (1*S*,5*R*)-salinilactone B **8** after 1-6 h preincubation time (mean of n = 2 ± SEM).

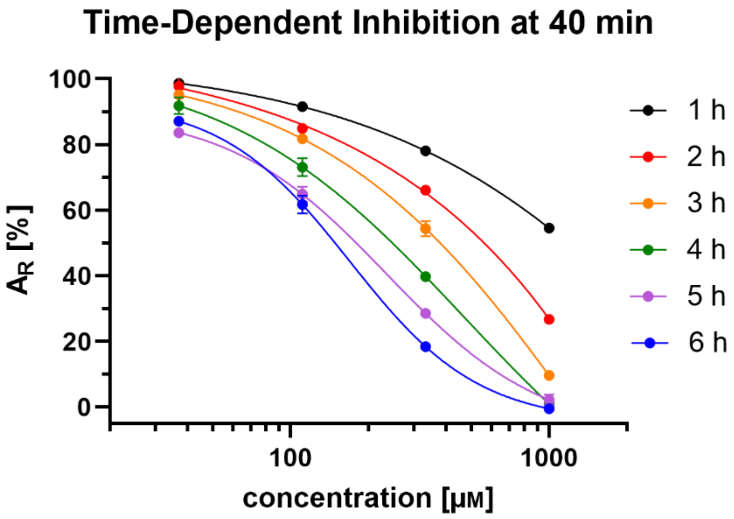

Figure S6. Preincubation time-dependent inhibition plot. The remaining enzyme activity (A_R) is plotted against the inhibitor concentration (mean of n = 2 ± SEM).

Determination of k_{obs}, k_{inact} and K_I was carried out according to literature.[3] The inactivation rate k_{obs} for every inhibitor concentration was determined by plotting the natural logarithm ($\ln(A_R)$) against the preincubation time (Figure 3D in the manuscript). Linear regression yielded the k_{obs} values in form of the negative slopes. Afterwards a double reciprocal plot of the k_{obs} values against the inhibitor concentrations was made. From the axis intercepts the constants can be calculated after k_{inact} = 1/y-intercept and K_I = -1/x-intercept (Figure 3D in the manuscript).

5. Inhibition of Aldehyde Dehydrogenase

The inhibition efficacy of both salinilactone B enantiomers was tested with an aldehyde dehydrogenase inhibitor screening kit (Sigma-Aldrich, MAK327). Commercially obtained disulfiram (Sigma-Aldrich, 86720) was used as a positive control. The provided protocol from the supplier was carried out with some modifications.

1. Dilution of ALDH to 2.2 U/mL (5.5 mg/mL) with assay buffer
2. Dilution of ALDH to 0.55 mg/mL with assay buffer and DTT solution (c = 160 µM) 18:1 (final concentration of DTT = 8 µM)
3. Addition of ALDH solution (0.55 mg/mL, 45 µL) to all needed wells in a 96 well plate (Greiner 655101)
4. Addition of inhibitor stock solution (salinilactone B: 5 mM, 0.5 mM, disulfiram: 500 µM, 100 µM, 5 µL) to the sample wells and DMSO (5 µL) to the vehicle and background wells
5. Place a foil (HJ-BIOANALYTIK GmbH, 900510) on the top of the plate and close the lid to reduce evaporation during incubation
6. Incubation for 6 h at 37 °C
7. Dilution of the 4X substrate solution to 1X with ultrapure water
8. Preparation of the reaction mixes, for each well 50 µL of the reaction mix is needed
    a. Mix for the inhibitor/vehicle wells: 45 µL assay buffer, 8 µL NAD/MTT, 1 µL diaphorase, 1X substrate solution
    b. Mix for the background wells: 45 µL assay buffer, 8 µL NAD/MTT, 1 µL diaphorase
9. Addition of the reaction mixes (50 µL) to the respective wells
10. Measurement of the optical density at λ = 565 nm every minute for 60 min

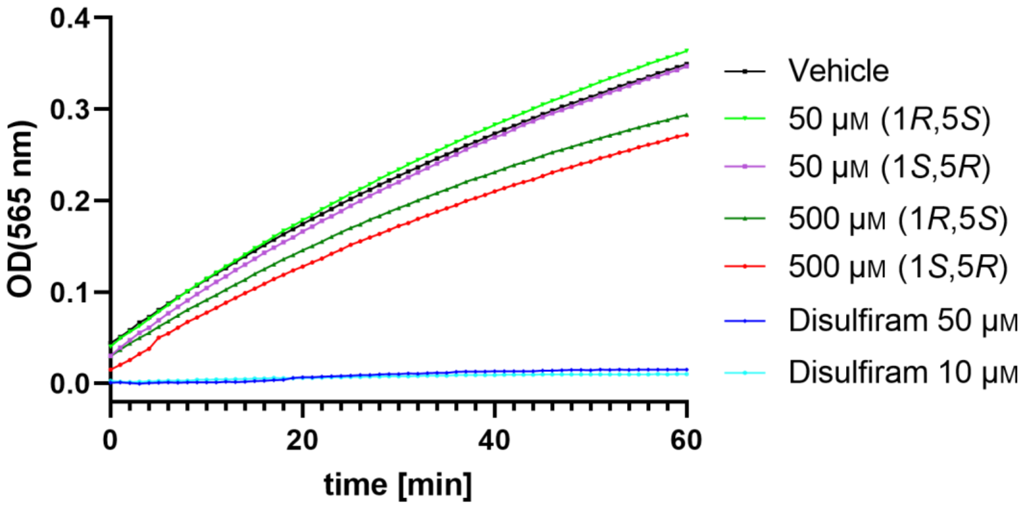

Figure S7. Time-dependent turbidity plot of ALDH inhibition by (1*R*,5*S*)-, (1*S*,5*R*)-salinilactone B and disulfiram after 6 h preincubation (n = 1).

6. Intact protein ESI measurement

For measuring the modification of intact rec. PDIA1 by the salinilactone B enantiomers, we carried out the following procedure.

1. Preparation of 50 mM Tris buffer pH 7.5
2. Dilution of the salinilactone B stock solutions (100 mM in DMSO) with Tris buffer to 2 mM (DMSO = 2%)
3. Preparation of DMSO-Tris buffer solution with 2% DMSO
4. Preparation of a DTT-Tris buffer solution with c = 32 µM
5. Dilution of rec. PDIA1 stock solution (0.8 mg/mL, in ultra pure water) (Novus Biologicals, NBP2-35195, *E. coli*) with DTT-Tris buffer from step 4. to 0.4 mg/mL
6. Pipetting salinilactone B-Tris buffer solution (25 µL) from step 2. and PDIA1-DTT-Tris buffer solution (25 µL) from step 5. together in one well of a 96 half area well plate
7. Pipetting DMSO-Tris buffer solution (25 µL) from step 3. and PDIA1-DTT-Tris buffer solution (25 µL) from step 5. together in one well of a 96 half area well plate
8. Put a foil (HJ-BIOANALYTIK GmbH, 900510) on the well plate and close the lid to minimize evaporation
9. Incubation for 1 h at 37 °C
   → final concentrations: PDIA1 = 200 µg/mL, salinilactone B = 1000 µM, DTT = 8 µM, DMSO = 1%

After the incubation time, samples were transferred to MS measuring tubes and subjected to LC-MS analysis. Ultra-high performance liquid chromatography (UHPLC) was carried out with a 1290 Agilent system. The column for separation was a Xbridge BEH C4, 300 Å, 3.5µm, 4.6×50 mm from Waters™. The following LC-conditions were applied.

Column temperature: 40 °C

Solvents: A: water with 0.1% formic acid
B: acetonitrile with 0.1% formic acid

Flow rate: 900 µL/min

The measurement of the mass spectra was done with a Bruker maXis™ HD UHR-TOF mass spectrometer with an Apollo II electrospray source. The following settings were applied.

Source type: ESI

Scan range: 500 – 4000 m/*z*

Ion polarity: positive

Capillary voltage: 4500 V

Nebulizer pressure: 4.0 bar

Dry heater: 200 °C

Dry gas: 9.0 L/min

Transfer time: 115 µs

Prepulse storage: 30 µs

Figure S8 shows the measured MS spectra of this experiment. The spectra were decovoluted with the Maximum Entropy function in the DataAnalysis 6.0 software. Deconvoluted spectra are shown in Figure 4A of the manuscript.

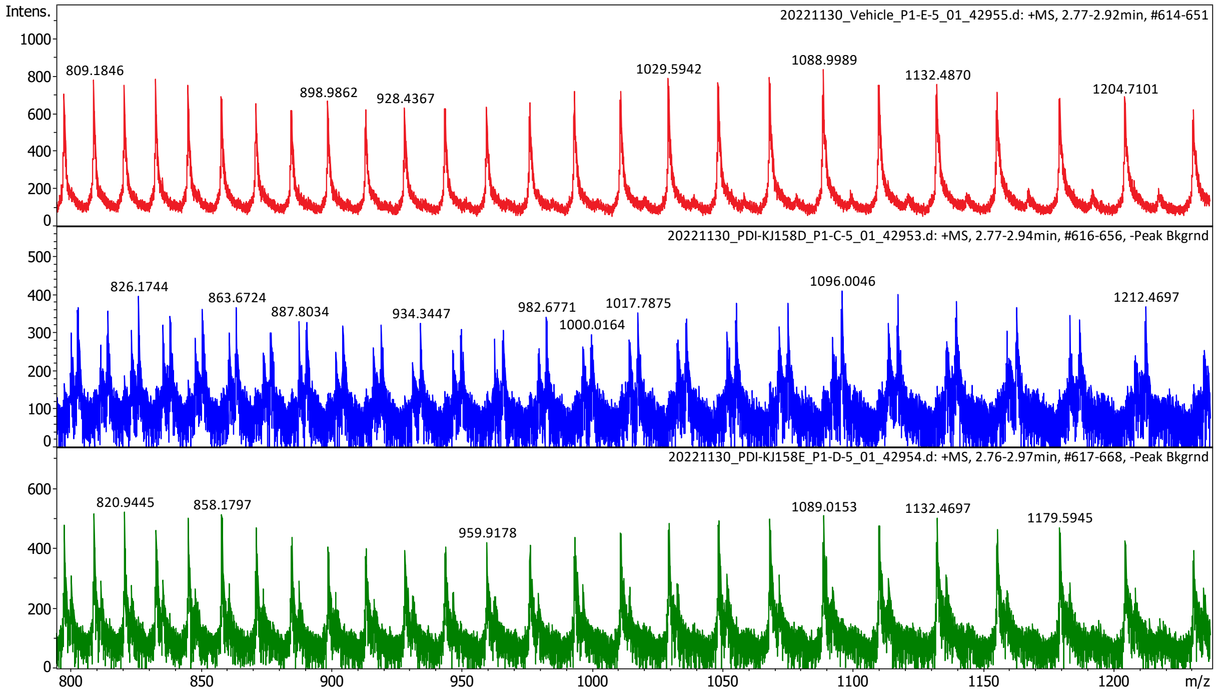

Figure S8. Unfiltered mass spectral data of PDIA1 incubated with a DMSO control (top in red), with (1*S*,5*R*)-salinilactone B **8** at 1 mM (middle in blue) and (1*R*,5*S*)-enantiomer **9** at 1 mM (bottom in green).

7. Binding site identification

The identification of the binding site of salinilactone B enantiomers was carried out according to the following protocol.

1. Preparation of 50 mM TEAB buffer pH 8.5 by dilution of 1 M stock solution with ultra pure water (Merck, 18597)
2. Preparation of a 80 µM DTT solution in 50 mM TEAB buffer
3. Preparation of a 500 µg/mL PDIA1 solution by addition of DTT-TEAB buffer (50 mM TEAB, 80 µM DTT) to a 1 mg/mL PDIA1 stock solution (20 mM Tris-HCl, 150 mM NaCl, 4 mM TCEP, pH 8.0, produced in *E. coli* by Dr. P. Lukat)
4. Dilution of salinilactone B stock solutions (26.6 mM and 8.85 mM in DMSO) or only DMSO with 50 mM TEAB buffer to c = 333 µM and 111 µM or 1.25% DMSO (V = 200 µL, 1.25% DMSO)
5. Addition of the diluted salinilactone B stock solutions and diluted DMSO (V = 80 µL) from step 4 to different wells of 96 well plate (Greiner 655101) (duplicates for each concentration and the DMSO negative control)
6. Addition of 500 µg/mL PDIA1 solution (V = 20 µL) from step 3 to the same wells from step 5
7. Place a foil (HJ-BIOANALYTIK GmbH, 900510) on the top of the well plate and close the lid to minimize evaporation during incubation
8. Incubation for 6 h at 37 °C
   → c(PDIA1) = 100 µg/mL, c(salinilactone B enantiomers) = 266 µM and 88.8 µM, DMSO = 1%, TCEP = 0.4 mM, DTT = 8 µM
9. After the incubation time, samples were transferred to individual 1.5 mL protein LoBind® tubes (Eppendorf)
10. Addition of a 50 mM TCEP solution in ultra pure water (10 µL) to the tubes
11. Incubation for 60 min at 56 °C and 300 rpm in an Eppendorf ThermoMixer C
12. Addition of 200 mM MMTS in *iso*-PrOH (5 µL) to the tubes
13. Incubation for 30 min at 24 °C and 300 rpm in an Eppendorf ThermoMixer C
14. Addition of Trypsin (Promega, V5111) (0.2 µg/mL, 5 µL) to the tubes
15. Incubation over night at 37 °C and 700 rpm in an Eppendorf ThermoMixer C
16. The samples were dried in a rotary vacuum concentrator RVC 2-18 CDplus from Christ at 30 °C and 1000 rpm
17. Addition of 0.1% formic acid (50 µL) to all samples

For MS/MS analyses, the peptides were loaded onto Evotips as described by the supplier Evosep:

1. Wash dry Evotips with 20 µL solvent B and centrifuge at 800 g for 60 s
2. Soak in 100 µL 2-propanol until all the tips are pale white
3. Equilibrate soaked Evotips with 20 µL solvent A and centrifuge at 800 g for 60 s
4. Load samples on wet tips (1 µL in formic acid) and centrifuge for 60 s at 800 g
5. Wash tips with 20 µL solvent A and centrifuge for 60 s at 800 g
6. Transfer 100 µL solvent A and centrifuge tips for 10 s at 800 g to keep tips wet

The peptides were eluted afterwards over an Evosep One HPLC connected to a timsTOF Pro from Bruker. Identification of the enriched peptides was done using the Peaks Studio 11 software with the following settings.

Search parameters: Parent Mass Error Tolerance: 20.0 ppm

Fragment Mass Error Tolerance: 0.03 Da

Precursor Mass Search Type: monoisotopic

Enzyme: Trypsin

Max Missed Cleavages: 1

Digest Mode: Specific

Fixed Modifications: Beta-methylthiolation: 45.99

Variable Modifications: Oxidation: 15.99

Sallinilactone B: 182.09

Max Variable PTM Per Peptide: 3

Database: UP_P07237_KJE21_20220630

Taxon: all species

Searched Entry: 1

Deep Learning Boost: Yes

FDR Estimation: Enabled

Peptide hit threshold (-10log*P*): 15

Filter RT: 0.0 – 21.0

Instrument parameters: Ion source: ESI(nano-spray)

Fragmentation Mode: CID, CAD(y and b ions)

MS Scan Mode: TimsTOF

MS/MS Scan Mode: TimsTOF

The identification of the binding site was done with the PTM (post-translational modification) profiling tool in the PEAKS Studio 11 software. The threshold was set to 2% minimal ion intensity. Only the assigned binding sites at C53 and C397 showed concentration dependency together with convincing MS2 spectra. Other putatively identified binding sites most likely result from incorrectly assigned fragments. This can occur, as shown by two DMSO negative controls in which putative binding sites were identified after the computer search despite the absence of salinilactone B (Figure S10). Quantification (LFQ, label free quantification) of the modified amino acids C53 and C397 was done by adding up the area under the curve of every feature (specific *m/z*, RT, charge, −10lg*P*) of the respective peptides identified in the entire measurement. A list of all peptides modified at C53 or C397 is shown in the tables below.

It has to be mentioned that cysteines, which were not modified by salinilactone B (+182.09) have been subsequently thiomethylated (+45.99) by MMTS as a necessary step in sample preparation.

Table S2. Quantification of modified peptides at C53 and C397 for (1*S*,5*R*)-enantiomer **8** (c = 88.8 µM, 50 eq. and 266 µM, 150 eq.).

**(1*S*,5*R*)-Sample1-50 eq.**					
**Sequence**	**Area**	***m/z***	**z**	**ppm**	**RT**
YLLVEFYAPWC[53](+182.09)GHC(+45.99)K	5.51E3	686.3144	3	-1.9	20.09
YLLVEFYAPWC[53](+182.09)GHC(+45.99)K	1.62E3	1028.9647	2	-5.1	20.09
YLLVEFYAPWC[53](+45.99)GHC(+45.99)K	4.46E4	640.9436	3	-7.3	20.07
YLLVEFYAPWC[53](+45.99)GHC(+45.99)K	1.2E2	640.9455	3	-4.4	20.79
YLLVEFYAPWC[53](+45.99)GHC(+45.99)K	3.77E2	640.9459	3	-3.8	20.01
YLLVEFYAPWC[53](+45.99)GHC(+45.99)K	3.94E2	640.9509	3	4.0	19.87
YLLVEFYAPWC[53](+45.99)GHC(+45.99)K	1.83E4	960.9121	2	-7.0	20.07
Total C53 modified by Salinilactone:<br>Total C53 not modified by Salinilactone:<br>Percentage C53 modified:	**7.13E3**<br>**6.38E4**<br>**10.1%**				
KNVFVEFYAPWC[397](+45.99)GHC(+45.99)K	5.97E2	505.7209	4	-6.0	19.82
KNVFVEFYAPWC[397](+45.99)GHC(+45.99)K	1.02E3	673.9592	3	-5.2	19.79
NVFVEFYAPWC[397](+182.09)GHC(+45.99)K	1.51E3	676.6286	3	-4.8	20.06
NVFVEFYAPWC[397](+182.09)GHC(+45.99)K	6.78E2	1014.4374	2	-6.6	20.05
NVFVEFYAPWC[397](+45.99)GHC(+45.99)K	2.67E2	631.2589	3	-8.7	20.78
NVFVEFYAPWC[397](+45.99)GHC(+45.99)K	5.67E4	631.2610	3	-5.3	20.04
NVFVEFYAPWC[397](+45.99)GHC(+45.99)K	1.11E3	631.2625	3	-3.1	19.95
NVFVEFYAPWC[397](+45.99)GHC(+45.99)K	5.03E2	631.2677	3	5.2	19.73
NVFVEFYAPWC[397](+45.99)GHC(+45.99)K	3.39E4	946.3845	2	-9.0	20.04
NVFVEFYAPWC[397](+45.99)GHC(+45.99)K	8.82E2	946.3846	2	-8.9	19.94
NVFVEFYAPWC[397](+45.99)GHC(+45.99)K	2.23E2	946.3948	2	1.9	19.73
Total C397 modified by Salinilactone:<br>Total C397 not modified by Salinilactone:<br>Percentage C397 modified:	**2.19E3**<br>**9.52E4**<br>**2.2%**				
**(1*S*,5*R*)-Sample2-50 eq.**					
**Sequence**	**Area**	***m/z***	**z**	**ppm**	**RT**
YLLVEFYAPWC[53](+182.09)GHC(+45.99)K	3.27E3	686.3140	3	-2.6	20.1
YLLVEFYAPWC[53](+182.09)GHC(+45.99)K	1.14E3	1028.9686	2	-1.3	20.1
YLLVEFYAPWC[53](+45.99)GHC(+45.99)K	3.27E4	640.9458	3	-3.9	20.07
YLLVEFYAPWC[53](+45.99)GHC(+45.99)K	1.24E4	960.9126	2	-6.5	20.08
Total C53 modified by Salinilactone:<br>Total C53 not modified by Salinilactone:<br>Percentage C53 modified:	**4.41E3**<br>**4.51E4**<br>**8.9%**				
KNVFVEFYAPWC[397](+45.99)GHC(+45.99)K	4.90E2	505.7193	4	-9.0	19.83
KNVFVEFYAPWC[397](+45.99)GHC(+45.99)K	1.73E3	673.9589	3	-5.6	19.83
NVFVEFYAPWC[397](+182.09)GHC(+45.99)K	6.76E2	676.6314	3	-0.6	20.06
NVFVEFYAPWC[397](+45.99)GHC(+45.99)K	4.84E4	631.2604	3	-6.4	20.04
NVFVEFYAPWC[397](+45.99)GHC(+45.99)K	6.13E3	631.2645	3	0.1	19.75

NVFVEFYAPWC^{397}(+45.99)GHC(+45.99)K	3.26E4	946.3854	2	-8.0	20.0
NVFVEFYAPWC^{397}(+45.99)GHC(+45.99)K	8.55E3	946.3878	2	-5.5	20.07
Total C397 modified by Salinilactone:<br>Total C397 not modified by Salinilactone:<br>Percentage C397 modified:	**6.76E2**<br>**9.57E4**<br>**0.7%**				
**(1*S*,5*R*)-Sample1-150 eq.**					
**Sequence**	**Area**	***m/z***	**z**	**ppm**	**RT**
SNFAEALAAHKYLLVEFYAPWC^{53}(+182.09)GHC(+45.99)K	4.35E2	799.8790	4	-3.4	20.01
YLLVEFYAPWC^{53}(+182.09)GHC(+45.99)K	3.23E4	686.3062	3	-14.0	20.08
YLLVEFYAPWC^{53}(+182.09)GHC(+45.99)K	1.84E4	686.3129	3	-4.1	20.13
YLLVEFYAPWC^{53}(+182.09)GHC(+45.99)K	1.11E4	1028.9664	2	-3.4	20.13
YLLVEFYAPWC^{53}(+182.09)GHC(+45.99)K	1.97E2	1028.9818	2	11.5	20.18
YLLVEFYAPWC^{53}(+45.99)GHC(+45.99)K	5.71E3	640.9440	3	-6.7	20.0
YLLVEFYAPWC^{53}(+45.99)GHC(+45.99)K	1.71E3	960.9134	2	-5.7	20.0
Total C53 modified by Salinilactone:<br>Total C53 not modified by Salinilactone:<br>Percentage C53 modified:	**6.24E4**<br>**7.42E3**<br>**89.4%**				
KNVFVEFYAPWC^{397}(+182.09)GHC(+45.99)K	2.44E2	719.3258	3	-6.0	19.94
NVFVEFYAPWC^{397}(+182.09)GHC(+45.99)K	4.10E3	676.628	3	-4.6	20.05
NVFVEFYAPWC^{397}(+182.09)GHC(+45.99)K	2.72E3	1014.4376	2	-6.4	20.05
NVFVEFYAPWC^{397}(+45.99)GHC(+45.99)K	3.15E3	631.2600	3	-7.0	19.93
NVFVEFYAPWC^{397}(+45.99)GHC(+45.99)K	1.65E3	946.3897	2	-3.4	19.93
Total C397 modified by Salinilactone:<br>Total C397 not modified by Salinilactone:<br>Percentage C397 modified:	**7.06E3**<br>**4.80E3**<br>**59.5%**				
**(1*S*,5*R*)-Sample2-150 eq.**					
**Sequence**	**Area**	***m/z***	**z**	**ppm**	**RT**
SNFAEALAAHKYLLVEFYAPWC^{53}(+182.09)GHC(+45.99)K	6.25E2	799.8786	4	-3.9	20.02
YLLVEFYAPWC^{53}(+182.09)GHC(+45.99)K	1.78E4	686.3113	3	-6.5	20.12
YLLVEFYAPWC^{53}(+182.09)GHC(+45.99)K	8.29E3	1028.9663	2	-3.5	20.09
YLLVEFYAPWC^{53}(+45.99)GHC(+45.99)K	1.74E4	640.9458	3	-3.9	20.04
YLLVEFYAPWC^{53}(+45.99)GHC(+45.99)K	6.62E3	960.9125	2	-6.6	20.02
Total C53 modified by Salinilactone:<br>Total C53 not modified by Salinilactone:<br>Percentage C53 modified:	**2.67E4**<br>**2.40E4**<br>**52.7%**				
NVFVEFYAPWC^{397}(+182.09)GHC(+45.99)K	4.38E3	676.6301	3	-2.5	20.05
NVFVEFYAPWC^{397}(+182.09)GHC(+45.99)K	2.46E3	1014.4432	2	-0.9	20.05
NVFVEFYAPWC^{397}(+182.09)GHC(+45.99)K	3.26E3	1014.4378	2	-6.2	20.05
NVFVEFYAPWC^{397}(+45.99)GHC(+45.99)K	1.73E2	631.2621	3	-3.6	19.94
NVFVEFYAPWC^{397}(+45.99)GHC(+45.99)K	1.77E4	631.2629	3	-2.4	19.99
NVFVEFYAPWC^{397}(+45.99)GHC(+45.99)K	1.05E4	946.3870	2	-6.3	19.98
Total C397 modified by Salinilactone:<br>Total C397 not modified by Salinilactone:<br>Percentage C397 modified:	**1.01E4**<br>**2.84E4**<br>**26.2%**				

The following MS2 spectra correspond to the best peptide modified by (1*S*,5*R*)-enantiomer **8** of the sample indicated in the upper right corner.

C53 Sample1-50eq.

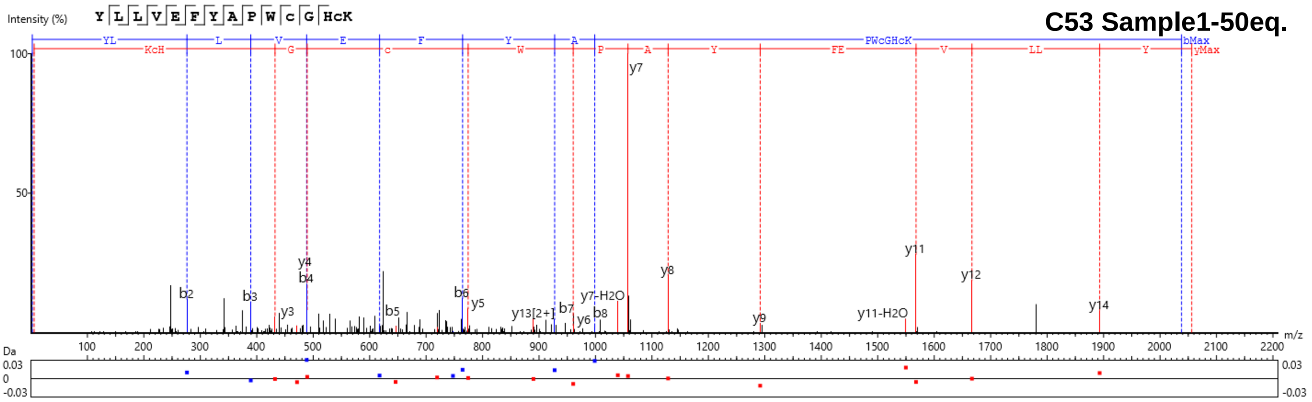

C53 Sample2-50eq.

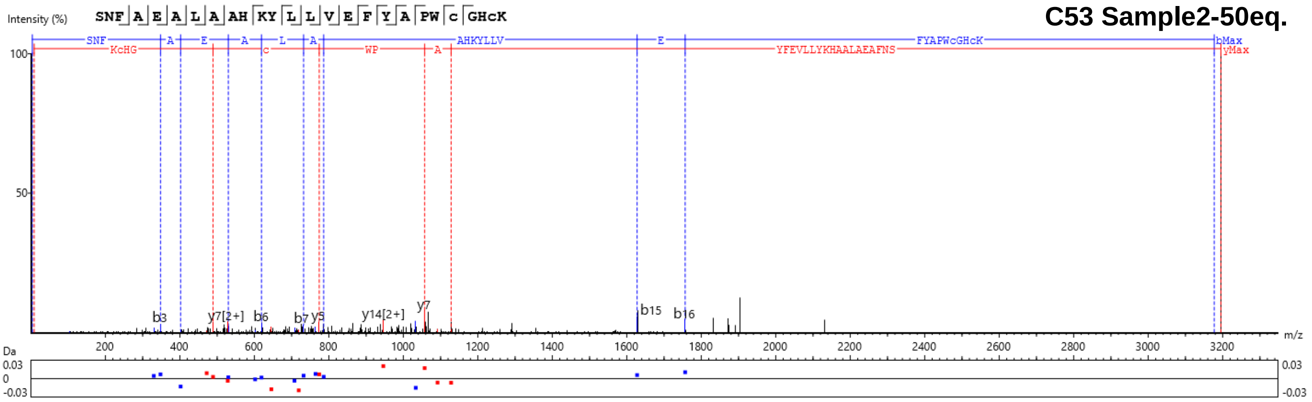

C397 Sample1-50eq.

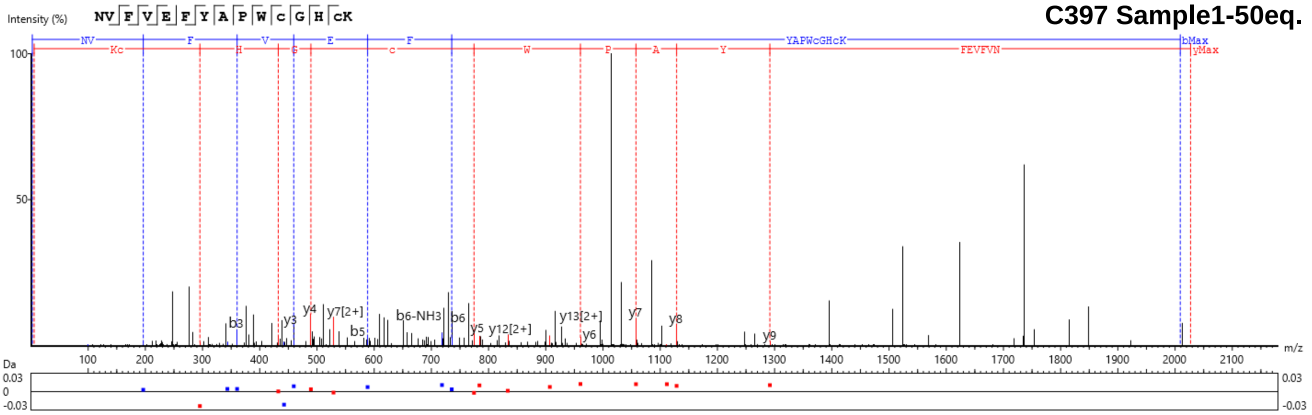

C397 Sample2-50eq.

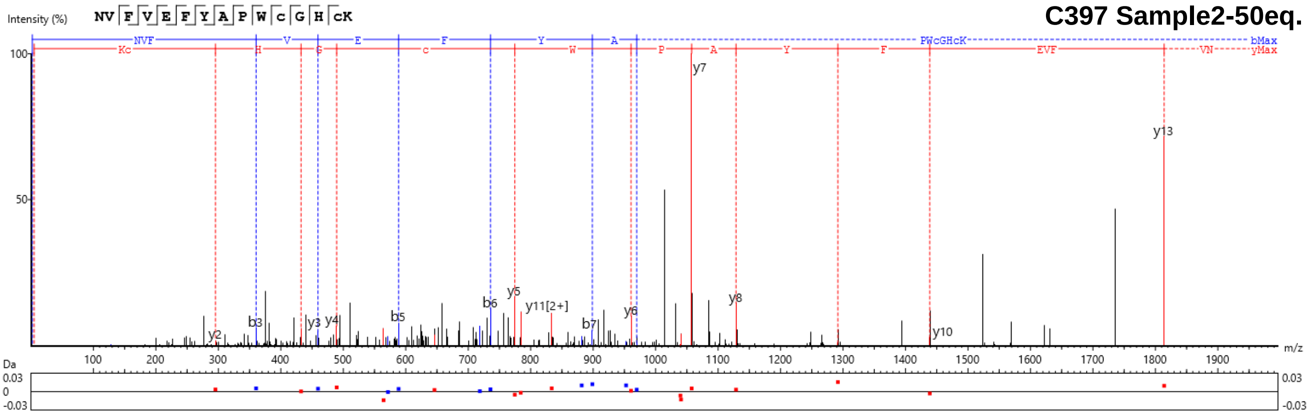

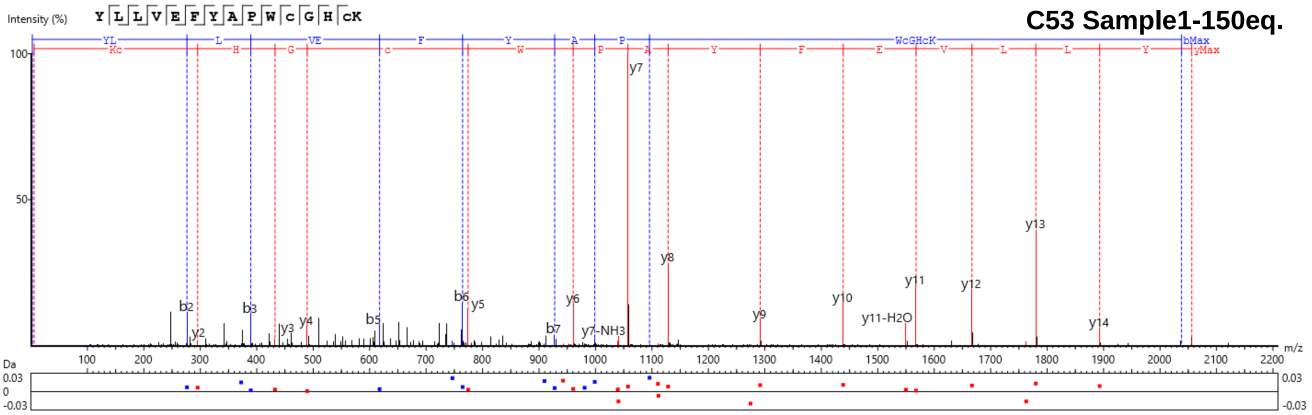
C53 Sample1-150eq.
Intensity (%)
YLLVEFYAPWcGHcK
y7
y13
y8
y11
y12
y10
y9
y14
b2
b3
b5
b6
y5
y6
b7
y7-NH3
y11-H2O
m/z
Da

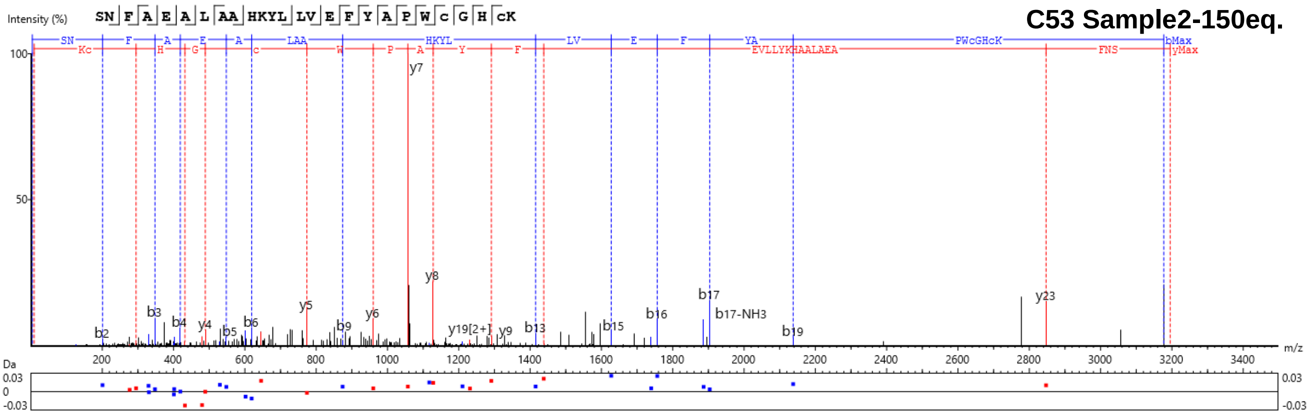
C53 Sample2-150eq.
Intensity (%)
SNFAEALAAHKYLLVEFYAPWcGHcK
y7
y8
y23
b17
b16
b17-NH3
b19
b15
b13
y19[2+]
y9
b9
y6
y5
b3
b4
y4
b5
b6
b2
m/z
Da

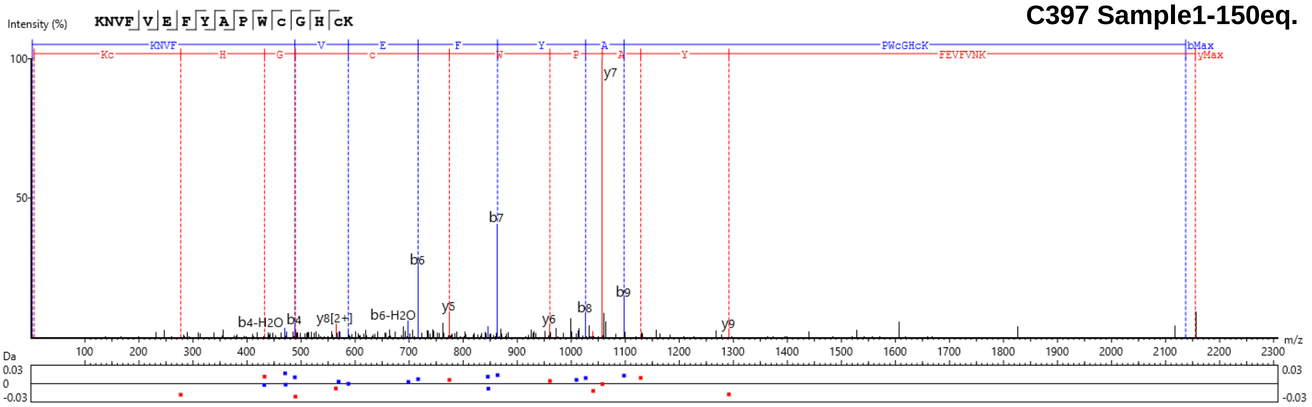
C397 Sample1-150eq.
Intensity (%)
KNVFVEFYAPWcGHcK
y7
b7
b6
b9
b8
y6
y5
y9
b4-H2O
b4
y8[2+]
b6-H2O
m/z
Da

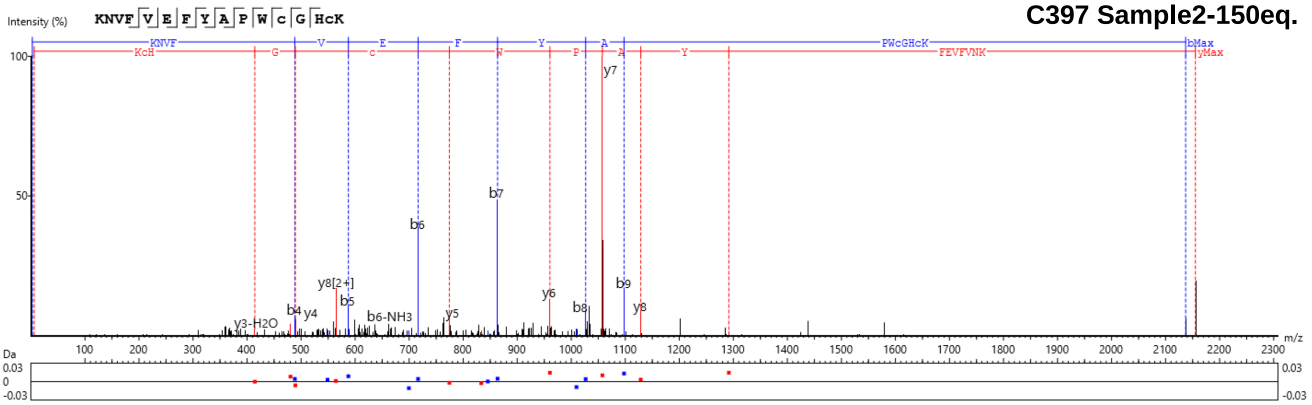
C397 Sample2-150eq.
Intensity (%)
KNVFVEFYAPWcGHcK
y7
b7
b6
b9
y6
b8
y8
y8[2+]
b5
b4
y4
y3-H2O
b6-NH3
y5
m/z
Da

Table S3. Quantification of modified peptides at C53 and C397 for (1*R*,5*S*)-enantiomer **9** (c = 88.8 µM, 50 eq. and 266 µM, 150 eq.).

**(1*R*,5*S*)-Sample1-50 eq.**					
**Sequence**	**Area**	***m/z***	**z**	**ppm**	**RT**
YLLVEFYAPW$\overset{53}{\text{C}}$(+182.09)GHC(+45.99)K	7.31E2	686.3123	3	-5.0	20.07
YLLVEFYAPW$\overset{53}{\text{C}}$(+45.99)GHC(+45.99)K	8.02E3	640.9446	3	-5.7	19.98
YLLVEFYAPW$\overset{53}{\text{C}}$(+45.99)GHC(+45.99)K	2.46E3	960.9183	2	-0.5	20.01
Total C53 modified by Salinilactone:<br>Total C53 not modified by Salinilactone:<br>Percentage C53 modified:	**7.31E2**<br>**1.05E4**<br>**6.5%**				
NVFVEFYAPW$\overset{397}{\text{C}}$(+45.99)GHC(+45.99)K	5.43E3	631.2599	3	-7.2	19.93
NVFVEFYAPW$\overset{397}{\text{C}}$(+45.99)GHC(+45.99)K	2.65E3	946.3875	2	-5.8	19.93
Total C397 modified by Salinilactone:<br>Total C397 not modified by Salinilactone:<br>Percentage C397 modified:	**0**<br>**8.08E3**<br>**0%**				
**(1*R*,5*S*)-Sample2-50 eq.**					
**Sequence**	**Area**	***m/z***	**z**	**ppm**	**RT**
YLLVEFYAPW$\overset{53}{\text{C}}$(+182.09)GHC(+45.99)K	1.83E3	686.3109	3	-7.1	20.07
YLLVEFYAPW$\overset{53}{\text{C}}$(+182.09)GHC(+45.99)K	6.17E2	1028.9648	2	-5.0	20.06
YLLVEFYAPW$\overset{53}{\text{C}}$(+45.99)GHC(+45.99)K	7.42E3	640.9446	3	-5.8	19.99
YLLVEFYAPW$\overset{53}{\text{C}}$(+45.99)GHC(+45.99)K	2.16E3	960.9153	2	-3.7	19.97
Total C53 modified by Salinilactone:<br>Total C53 not modified by Salinilactone:<br>Percentage C53 modified:	**2.45E3**<br>**9.58E3**<br>**20.4%**				
NVFVEFYAPW$\overset{397}{\text{C}}$(+182.09)GHC(+45.99)K	8.48E2	676.6287	3	-4.6	20.02
NVFVEFYAPW$\overset{397}{\text{C}}$(+45.99)GHC(+45.99)K	4.84E3	631.2604	3	-6.3	19.92
NVFVEFYAPW$\overset{397}{\text{C}}$(+45.99)GHC(+45.99)K	2.17E3	946.3917	2	-1.4	19.92
Total C397 modified by Salinilactone:<br>Total C397 not modified by Salinilactone:<br>Percentage C397 modified:	**8.48E2**<br>**7.01E3**<br>**10.8%**				
**(1*R*,5*S*)-Sample1-150 eq.**					
**Sequence**	**Area**	***m/z***	**z**	**ppm**	**RT**
YLLVEFYAPW$\overset{53}{\text{C}}$(+182.09)GHC(+45.99)K	1.32E3	686.3138	3	-2.8	20.09
YLLVEFYAPW$\overset{53}{\text{C}}$(+182.09)GHC(+45.99)K	2.89E2	1028.9661	2	-3.8	20.09
YLLVEFYAPW$\overset{53}{\text{C}}$(+45.99)GHC(+45.99)K	4.75E3	640.9444	3	-6.1	20.0
YLLVEFYAPW$\overset{53}{\text{C}}$(+45.99)GHC(+45.99)K	1.38E3	960.9117	2	-7.4	20.0
Total C53 modified by Salinilactone:<br>Total C53 not modified by Salinilactone:<br>Percentage C53 modified:	**1.61E3**<br>**6.13E3**<br>**20.8%**				
NVFVEFYAPW$\overset{397}{\text{C}}$(+182.09)GHC(+45.99)K	6.48E2	676.6312	3	-1.0	20.05
NVFVEFYAPW$\overset{397}{\text{C}}$(+45.99)GHC(+45.99)K	3.42E3	631.2601	3	-6.8	19.94
NVFVEFYAPW$\overset{397}{\text{C}}$(+45.99)GHC(+45.99)K	1.45E3	946.3867	2	-6.7	19.94
Total C397 modified by Salinilactone:<br>Total C397 not modified by Salinilactone:<br>Percentage C397 modified:	**6.48E2**<br>**4.87E3**<br>**11.7%**				
**(1*R*,5*S*)-Sample2-150 eq.**					
**Sequence**	**Area**	***m/z***	**z**	**ppm**	**RT**
YLLVEFYAPW$\overset{53}{\text{C}}$(+182.09)GHC(+45.99)K	4.21E3	686.3124	3	-4.8	20.08
YLLVEFYAPW$\overset{53}{\text{C}}$(+182.09)GHC(+45.99)K	1.47E3	1028.9679	2	-2.0	20.08
YLLVEFYAPW$\overset{53}{\text{C}}$(+45.99)GHC(+45.99)K	1.09E4	640.9450	3	-5.1	20.01

53<br>YLLVEFYAPWC(+45.99)GHC(+45.99)K	3.40E3	960.9129	2	-6.2	20.0
Total C53 modified by Salinilactone:<br>Total C53 not modified by Salinilactone:<br>Percentage C53 modified:	**5.68E3**<br>**1.43E4**<br>**28.4%**				
397<br>KNVFVEFYAPWC(+45.99)GHC(+45.99)K	8.51E1	673.9546	3	-12.1	19.84
397<br>NVFVEFYAPWC(+182.09)GHC(+45.99)K	1.99E3	676.6259	3	-3.5	20.05
397<br>NVFVEFYAPWC(+45.99)GHC(+45.99)K	5.77E3	631.2604	3	-6.4	19.94
397<br>NVFVEFYAPWC(+45.99)GHC(+45.99)K	2.56E3	946.3871	2	-6.2	19.94
Total C397 modified by Salinilactone:<br>Total C397 not modified by Salinilactone:<br>Percentage C397 modified:	**1.99E3**<br>**8.42E3**<br>**19.1%**				

The following MS2 spectra correspond to the best peptide modified by (1*R*,5*S*)-enantiomer **9** of the sample indicated in the upper right corner.

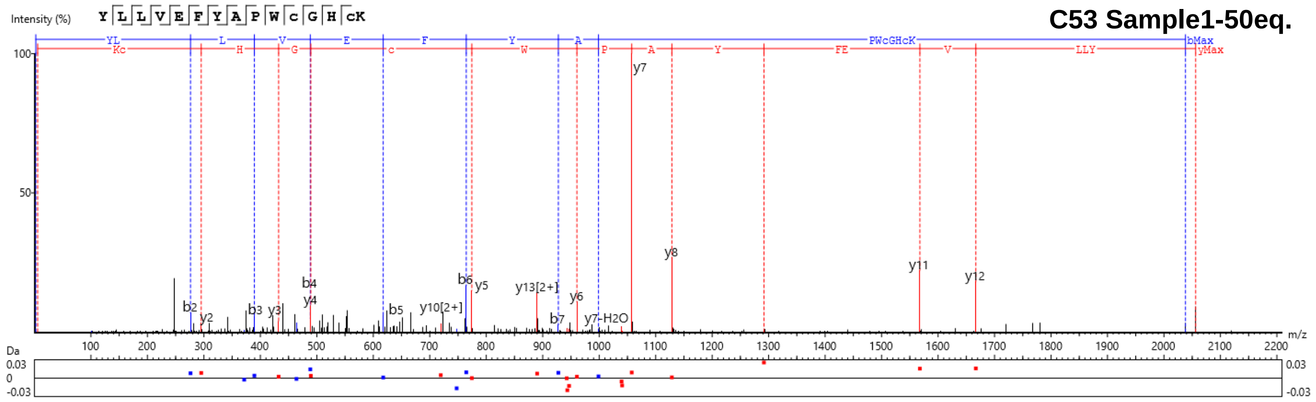

C53 Sample1-50eq.

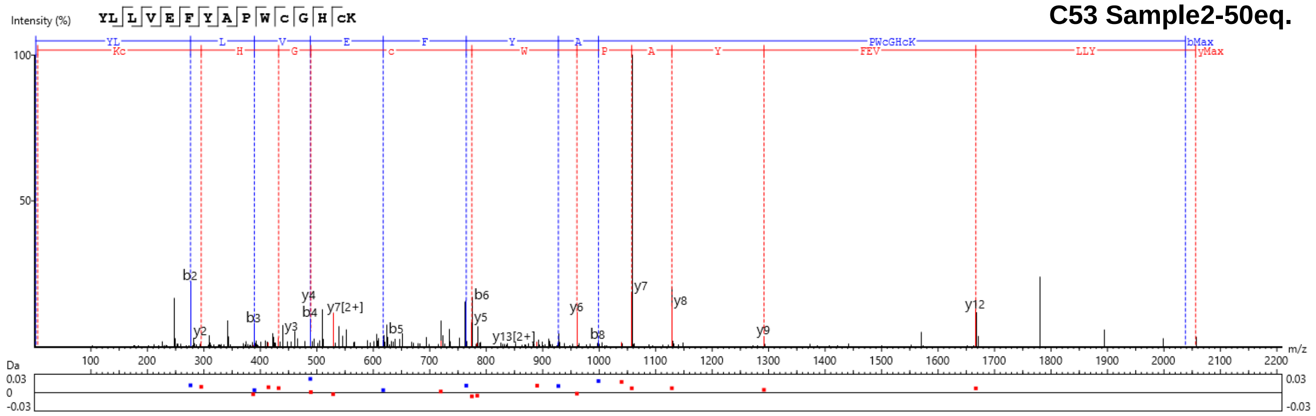

C53 Sample2-50eq.

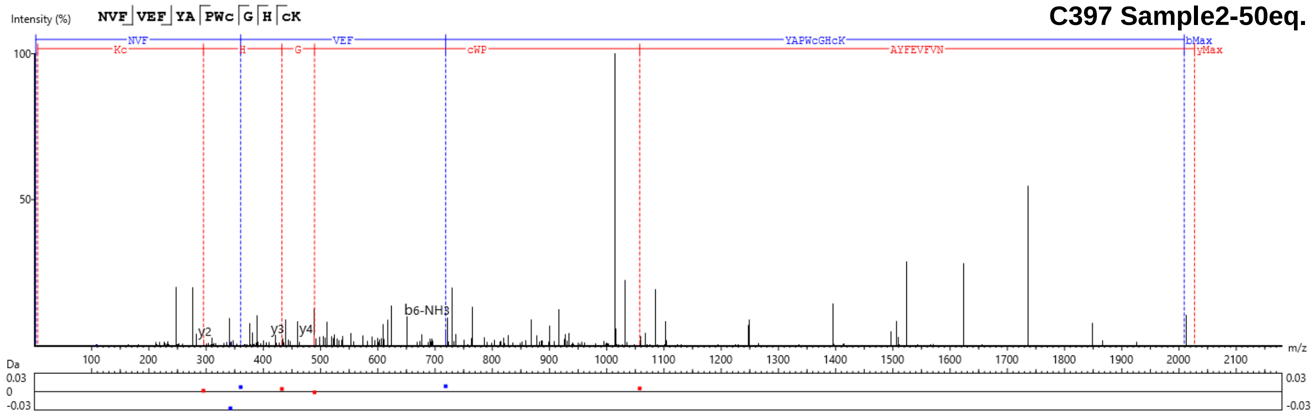

C397 Sample2-50eq.

C53 Sample1-150eq.

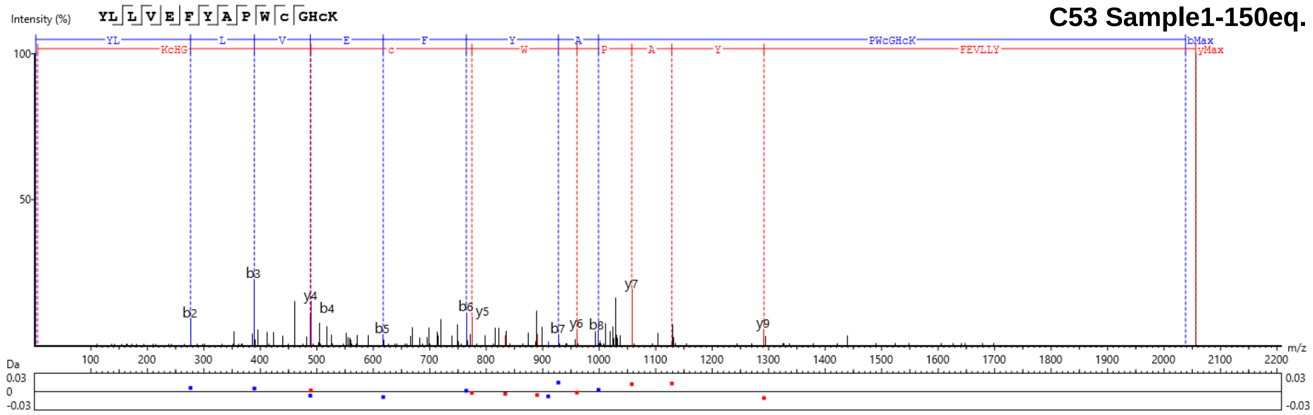

C53 Sample2-150eq.

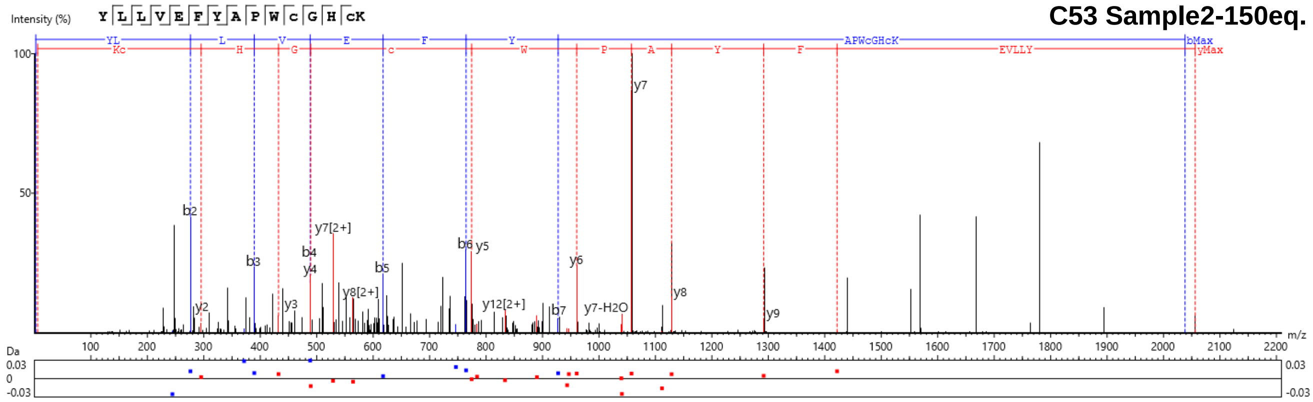

C397 Sample1-150eq.

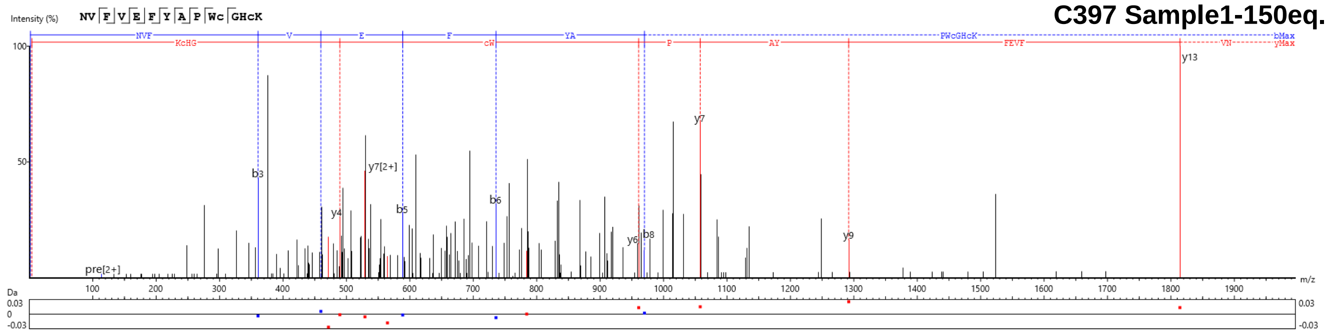

C397 Sample2-150eq.

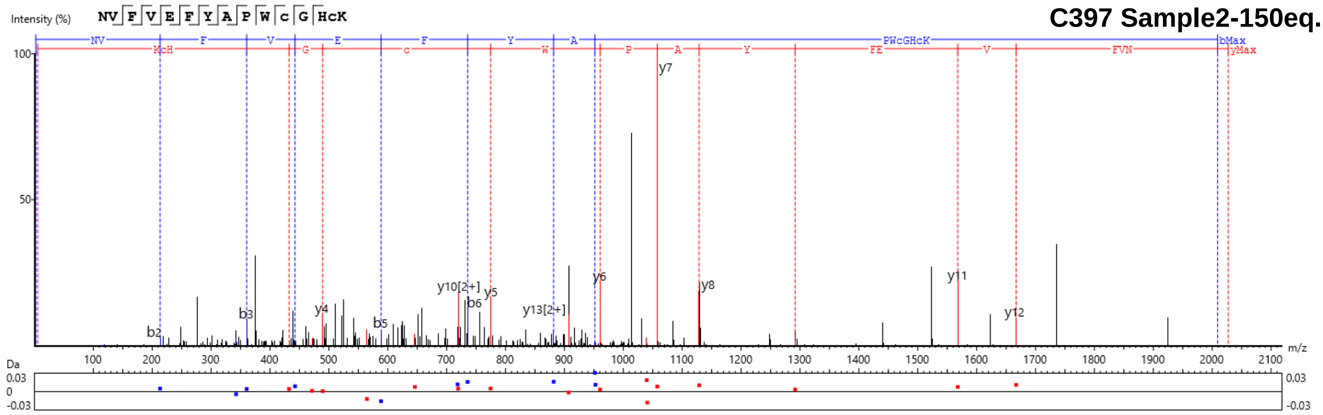

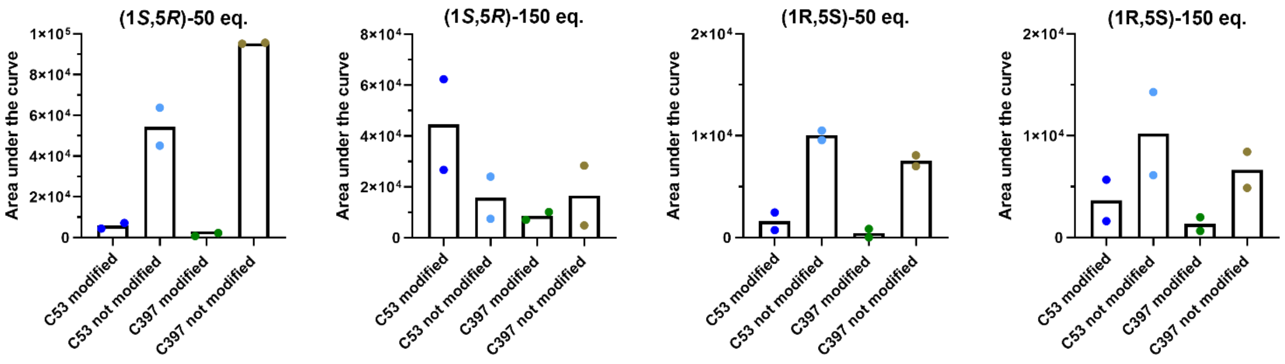

Figure S9. Quantification of all identified features of the catalytic active site peptides modified and not modified by salinilactone B (n = 2).

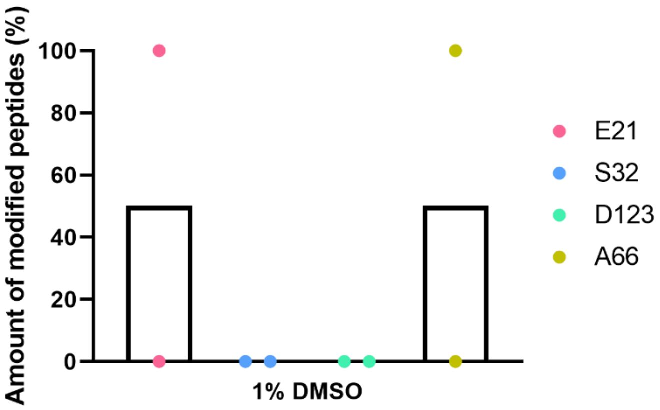

Figure S10. Putatively identified amino acids modified by salinilactone B of samples incubated with 1% DMSO as negative control. The threshold for binding site identifications was set to ion intensity of 2% (n = 2).

8. Fluorophore labeling experiments

Fluorescence labeling SDS Gel

The fluorescence labeling experiment with a gel was done according to the following protocol.

1. 5 · 10^5 A549 cells were seeded in four wells of a 6 well plate in 2 mL media (gibco DMEM with GlutaMAX + 10% gibco FBS)
2. Incubation for 8 h at 37 °C and 10% CO_2
3. Addition of the BODIPY-salinilactone conjugate **23** (10 mM in DMSO) (20 µL) to two wells
   → concentration: conjugate **23** = 100 µM, DMSO = 1%
4. Addition of DMSO (20 µL) to the other two wells
   → concentration: DMSO = 1%
5. Incubation over night at 37 °C and 10% CO_2
6. Removing media
7. Washing cells with PBS (2 × 3 mL)
8. Addition of Accutase® (BioLegend) (300 µL) to the wells
9. Incubation for 3 min at 37 °C
10. Addition of 2.7 mL media (gibco DMEM with GlutaMAX + 10% gibco FBS) to the detached cells
11. Transfer of the cells to different 15 mL Falkon™ tubes
12. Centrifugation for 5 min at 1000 rpm
13. Removing media from the tubes
14. Washing all cell pellets with PBS (1 × 3 mL)
15. Centrifugation for 5 min at 1000 rpm
16. Removing PBS
17. Addition of 0.1% Triton™ X-100 (Sigma-Aldrich T8787) (40 µL) in ultra pure water and protease inhibitor (Pierce™ A32963) (10 µL) in ultra pure water
18. Transfer to different 1.5 mL protein LoBind® tubes (Eppendorf)
19. Cell lysis for 20 min on ice, vortex every 10 min
20. Addition of 3X Laemmli sample buffer (25 µL)
21. Heating for 6 min at 96 °C in a Eppendorf ThermoMixer C
22. Preparation of two 1 mm thick SDS gels (10% polyacrylamide in the resolving gel and 5% polyacrylamide in the stacking gel), one gel was prepared with 1 vol.-% of 2,2,2-trichloroethanol (Sigma-Aldrich, T54801) in the resolving gel for cell lysate visualization
23. Loading the gels with 25 µL of each sample
24. Run the gel
25. Gel visualization with a Chemidoc system from Bio-Rad (gel with trichloroethanol: visualization with the Stain-Free gel settings; gel without trichloroethanol: visualization with Flamingo filter because of its similar fluorescence properties to BODIPY)

The pictures of the gels are in the manuscript Figure 5.

Fluorescence labeling and fluorescence microscopy

The fluorescence labeling experiment with a spinning disk microscope was done according to the following procedure.

1. Dilution of fibronectin stock solution (1 mg/mL in ultra pure water) (Roche, 11051407001) with PBS to 25 µg/mL
2. Addition of the diluted fibronectin solution (150 µL) to a 8 well chamber slide system (Thermo Scientific™, Nunc™ Lab-Tek™, 177445)
3. Incubation for 30 min at RT
4. Removing fibronectin
5. Washing wells with PBS (300 µL), removing afterwards
6. Seeding $1 \cdot 10^5$ cells/mL in media (gibco DMEM with GlutaMAX + 10% gibco FBS) (200 µL) in the wells
7. Incubation for 5 h at 37 °C and 10% CO_2
8. Addition of BODIPY-salinilactone conjugate **23** (stock solution 10 mM in DMSO) (2 µL) or BODIPY-iodide **21** (stock solution 10 mM in DMSO) (2 µL) to the cells as a negative control
   → concentration: BODIPY-salinilactone conjugate **23** and BODIPY-iodide **21** = 100 µM, DMSO = 1%
9. Incubation for 18 h at 37 °C and 10% CO_2
10. Removing media
11. Washing cells with PBS (1 × 300 µL), removing afterwards
12. Fixation of the cells with −20 °C EtOH (300 µL) for 10 min at RT
13. Sequential rehydration of the cells with PBS (removing 150 µL, addition of 150 µL PBS, three times repetition)
14. Removal of PBS
15. Permeabilization of the cells with 0.2% Triton™ X-100 (Sigma-Aldrich T8787) in PBS (300 µL) for 5 min at RT
16. Washing cells thrice with PBS (3 × 400 µL), remove afterwards
17. Addition of 1% BSA in PBS as blocking buffer (A7030, Sigma-Aldrich) (300 µL) for 1 h at RT, remove afterwards
18. Addition of PDI antibody (Proteintech, CL594-66422) (24 µL) to PBS (2376 µL) (dilution = 1:100)
19. Addition of diluted antibody (200 µL) from step 18 to all wells
20. Incubation for 2 h at RT
21. Removing PBS and washing cells thrice with PBS (3 × 400 µL, 5 min), removing PBS afterwards
22. Removing well chambers and silicone seal
23. Applying 2-3 drops of Roti®Mount FluorCare DAPI (Carl Roth, HP20.1)
24. Putting on cover glass (24 × 60 mm)
25. Sample drying over night
26. Fluorescence microscopy (Spinning Disk Confocal)

The three channels for fluorescence detection were measured with the following conditions:

Channel 1: PDI-CoraLite®594 AB

Exposure: 700 ms

Readout Mode: Rolling shutter at 12-bit

Readout Rate: 540 MHz

Conversion Gain: Gain 4

NIDAQ, MultiLaser: ExW: 561 nm, Power: 70.9

EmW: 600/52 nm, single bandpass filter

Channel 2: BODIPY-salinilactone conjugate **23**

Exposure: 200 ms

Readout Mode: Rolling shutter at 12-bit

Readout Rate: 540 MHz

Conversion Gain: Gain 4

NIDAQ, MultiLaser: ExW: 488 nm, Power: 30.0

EmW: 525/50 nm, single bandpass filter

Channel 3: DAPI

Exposure: 300 ms

Readout Mode: Rolling shutter at 12-bit

Readout Rate: 540 MHz

Conversion Gain: Gain 4

NIDAQ, MultiLaser: ExW: 405 nm, Power: 42.4

EmW: 447/60 nm, single bandpass filter

Specific fluorescence labeling of PDIs was only possible with the BODIPY-salinilactone conjugate **23** (Manuscript Figure 6). In contrast to that, BODIPY-iodide **21** resulted in a non-specific fluorescence signal throughout the well (Figure S11).

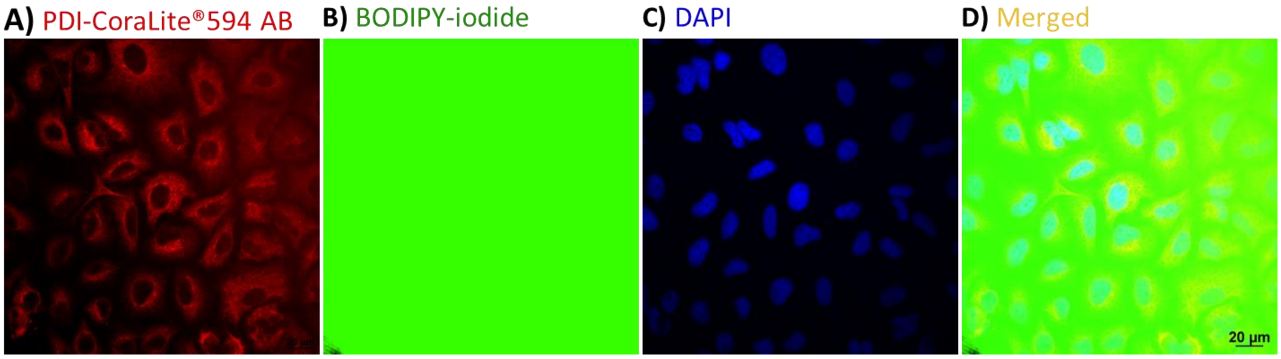

Figure S11. Spinning disk microscopy images of BODIPY-iodide **21** as control. A) CoraLite®594 fluorophore-tagged anti-PDI monoclonal antibody. B) Fluorescence detection of **21** (λ_{em} = 525/50 nm). C) DAPI staining of nuclei. D) The overlay of signals from A-C.

Pictures of the fluorescence microscopy with the BODIPY-salinilactone conjugate **23** are shown in the manuscript Figure 6.

9. Synthetic procedures and compound characterization

Biotin salinilactone conjugate 1

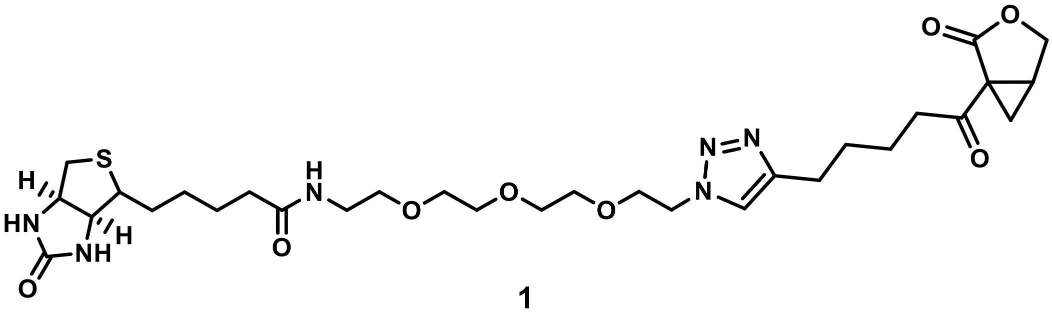

1

Under argon atmosphere 1-(hept-6-ynoyl)-3-oxabicyclo[3.1.0]hexan-2-one* (12.1 mg, 58.7 µmol, 1 eq.), biotin-PEG3-azide (57.2 mg, 129 µmol, 2.2 eq.), tris(3-hydroxypropyltriazolylmethyl)amine (5.94 mg, 13.7 µmol, 0.2 eq.), sodium ascorbate (6.18 mg, 31.2 µmol, 0.5 eq.) and copper (II) sulfate (2.11 mg, 13.2 µmol, 0.2 eq.) were dissolved in dry dimethyl sulfoxide (0.2 mL) and dry *tert*-butyl alcohol (0.2 mL). The reaction was stirred for 2 h at room temperature followed by 21 h at 60 °C and 1 h at 80 °C. Because of the very slow reaction progress, additional amounts of copper (II) sulfate and sodium ascorbate were added (~15 mg each). The reaction was stirred for additional 2 h at 80 °C. The reaction was cooled to room temperature and diluted with ethyl acetate (50 mL). The organic phase was washed with deion. water (3 × 25 mL), dried over sodium sulfate, filtered and volatiles removed under reduced pressure. Due to the unexpected water solubility of the product, the water phase from the washing step was saturated with sodium chloride and again extracted with ethyl acetate (3 × 60 mL). The second organic phase was dried over sodium sulfate, filtered and combined with the first obtained crude product. After removing the solvent under reduced pressure, the crude material was purified by column chromatography (CH_2Cl_2/MeOH, 97:3 to 90:10). The product was obtained as a colorless solid (8.54 mg, 13.1 µmol, 22%).

*The starting material was kindly provided by Dr. C. Schlawis[4]

^{1}H NMR (500 MHz, acetone-d_6): δ = 7.74 (s, 1H), 7.15 (s. 1H), 5.99 (s, 1H), 5.73 (s, 1H), 4.52 (t, ^3J = 5.4 Hz, 2H), 4.50-4.48 (m, 1H), 4.37 (dd, ^3J = 9.3 Hz, ^4J = 4.8 Hz, 1H), 4.34-4.31 (m, 1H), 4.19 (d, ^3J = 9.3 Hz, 1H), 3.88 (t, ^3J = 5.4 Hz, 2H), 3.61-3.54 (m, 8H), 3.50 (t, ^3J = 5.8 Hz, 2H), 3.33 (m_c, 2H), 3.21 (m_c, 1H), 3.12-3.04 (m, 1H), 2.96-2.86 (m, 2H), 2.79 (m_c, 1H), 2.72-2.67 (m, 3H), 2.18 (t, ^3J = 7.2 Hz, 2H), 1.95 (dd, ^3J = 8.0 Hz, ^4J = 4.1 Hz, 1H), 1.82-1.73 (m, 1H), 1.72-1.58 (m, 7H), 1.50-1.41 (m, 2H), 1.40 (dd, ^3J = 5.6 Hz, ^4J = 4.1 Hz, 1H) ppm.

^{13}C NMR (175 MHz, acetone-d_6): δ = 202.8, 173.7, 173.1, 163.7, 147.9, 122.7, 71.2, 71.1, 71.1, 70.9, 70.5, 70.3, 68.1, 62.3, 60.8, 56.5, 50.5, 41.5, 41.0, 39.8, 36.9, 36.2, 30.6, 29.7, 29.1, 29.1, 26.4, 26.1, 23.7, 23.1 ppm.

ESI-HRMS: calc. for $C_{30}H_{47}N_6O_8S$ $[M+H]^+$: m/z = 651.3171; found: 651.3167

Allyl hept-2-ynoate (3)

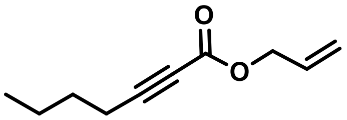

3

1-Hexyne (**2**) (2.00 g, 2.79 mL, 24.3 mmol, 1 eq.) was dissolved in 40 mL dry tetrahydrofuran and cooled to −78 °C. A 2.5 M solution of *n*-BuLi in tetrahydrofuran (9.74 mL, 24.3 mmol, 1 eq.) was added dropwise to the solution followed by stirring for 35 min at this temperature. After adding allyl chloroformate (2.93 g, 2.6 mL, 24.3 mmol, 1 eq.) the reaction was stirred 1 h at −78 °C. The reaction was warmed up to room temperature within 45 min and afterwards quenched with aq. sat. NH_4Cl solution (40 mL). The quenched solution was added to further aq. sat. NH_4Cl solution (100 mL) and extracted with Et_2O (3 × 100 mL). The organic phase was dried over sodium sulfate, filtered and volatiles were removed under reduced pressure. The crude product was purified by column chromatography (cyclohexane/EtOAc, 97.5:2.5) to obtain a colorless oil (3.64 g, 21.9 mmol, 90%).

^{1}H NMR (500 MHz, MeCN-d_3): δ = 5.94 (ddt, ^3J = 17.2 Hz, ^3J = 10.5 Hz, ^3J = 5.8 Hz, 1H), 5.33 (dq, ^3J = 17.2 Hz, $^{2/4}J$ = 1.6 Hz, 1H), 5.26 (dq, ^3J = 10.5 Hz, $^{2/4}J$ = 1.3 Hz, 1H), 4.62 (dt, ^3J = 5.8 Hz, ^4J = 1.4 Hz, 2H), 2.37 (t, ^3J = 7.1 Hz, 2H), 1.57-1.51 (m, 2H), 1.45-1.37 (m, 2H), 0.91 (t, ^3J = 7.4 Hz, 3H) ppm.

^{13}C NMR (125 MHz, MeCN-d_3): δ = 154.1, 132.9, 119.3, 90.9, 73.6, 67.0, 30.3, 22.7, 18.7, 13.8 ppm.

ESI-HRMS: calc. for $C_{10}H_{14}O_2Na$ $[M+Na]^+$: *m/z* = 189.0886; found: 189.0886

1-Pentanoyl-3-oxabicyclo[3.1.0]hexan-2-one (4) (racemic mixture)

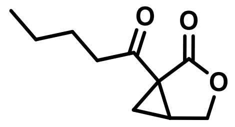

4

This compound was synthesized according to literature with changes in the purification.[5] Allyl hept-2-ynoate (**3**) (1.12 g, 6.74 mmol, 1 eq.) was dissolved in 100% acetic acid (67.4 mL) under argon atmosphere. (Diacetoxyiodo)benzene (4.34 g, 13.5 mmol, 2 eq.) and palladium(II) acetate (155 mg, 690 µmol, 0.1 eq.) was added to the solution. The reaction was stirred for 4 h at 80 °C. After cooling to room temperature the reaction was quenched with deion. Water (150 mL). The aqueous phase was extracted with ethyl acetate (3 × 100 mL). The organic phase was washed with aq. sat. NaCl solution (3 × 100 mL), dried over sodium sulfate, filtered and volatiles were removed under reduced pressure. The crude was purified by column chromatography (toluene/MeCN, 99:1 to 95:5) to obtain a colorless oil (793 mg, 4.35 mmol, 65%). For the usage in assays and MS/MS analysis, a part of the product was further purified by HPLC (water/MeCN).

^{1}H NMR (500 MHz, MeCN-d_3): δ = 4.30 (dd, ^2J = 9.3 Hz, ^3J = 4.8 HZ, 1H), 4.14 (d, ^2J = 9.3 Hz, 1H), 2.99 (ddd, ^2J = 17.7 Hz, ^3J = 8.3 Hz, ^3J = 6.5 Hz, 1H), 2.80 (ddd, ^2J = 17.7 Hz, ^3J = 8.3 Hz, ^3J = 6.5 Hz, 1H), 2.70 (dddd, ^3J = 7.9 Hz, ^3J = 5.7 Hz, ^3J = 4.8 Hz, ^3J = 0.9 Hz, 1H), 1.92 (dd, ^3J = 7.9 Hz, ^2J = 4.2 Hz, 1H), 1.60-1.46 (m, 2H), 1.37 (dd, ^3J = 5.7 Hz, ^2J = 4.2 Hz, 1H), 1.32 (m$_c$, 2H), 0.90 (t, ^3J = 7.4 Hz, 3H) ppm.

^{13}C NMR (125 MHz, MeCN-d_3): δ = 203.6, 174.3, 68.4, 41.6, 37.1, 30.7, 26.3, 23.6, 22.9, 14.2 ppm.

ESI-HRMS: calc. for $C_{10}H_{14}O_3Na$ $[M+Na]^+$: m/z = 205.0835; found: 205.0835

$[\alpha]_D^{20}$ = 0.20° (c = 0.663, $CHCl_3$)

1-(1,1-Dimethoxypentyl)-3-oxabicyclo[3.1.0]hexan-2-one (5) (racemic mixture)

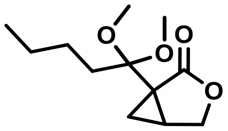

5

Under argon atmosphere 1-pentanoyl-3-oxabicyclo[3.1.0]hexan-2-one (**4**) (580 mg, 2.04 mmol, 1 eq.) and *p*-toluenesulfonic acid monohydrate (48.1 mg, 253 µmol, 0.1 eq.) were dissolved in dry MeOH (5 mL). Trimethyl orthoformate (2.16 g, 2.23 mL, 20.4 mmol, 10 eq.) was added and the reaction was stirred at room temperature for 17 h. The reaction was added to aq. sat. $NaHCO_3$ solution (150 mL) followed by extraction with methyl *tert*-butyl ether (4 × 100 mL). The organic phase was dried over sodium sulfate, filtered and volatiles were removed under reduced pressure to obtain a colorless oil. The crude was used without purification.

(1*R*,5*S*)-1-((4*R*,5*R*)-2-butyl-4,5-diphenyl-1,3-dioxolan-2-yl)-3-oxabicyclo[3.1.0]hexan-2-one (6)

and

(1*S*,5*R*)-1-((4*R*,5*R*)-2-butyl-4,5-diphenyl-1,3-dioxolan-2-yl)-3-oxabicyclo[3.1.0]hexan-2-one (7)

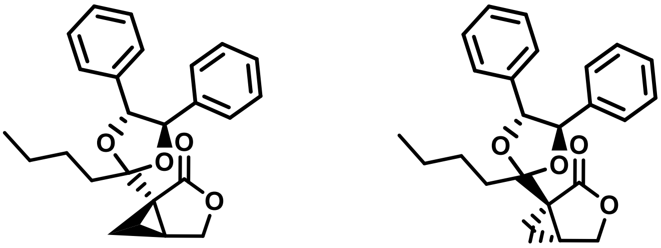

6 and **7**

Under argon atmosphere (*R*,*R*)-(+)-hydrobenzoin (1.46 g, 6.81 mmol, 2.1 eq.) and camphorsulfonic acid (197 mg, 848 µmol, 0.3 eq) were dissolved in dry MeCN (15 mL) and stirred at room temperature for 30 min together with 3 Å (five spatula). 1-(1,1-Dimethoxypentyl)-3-oxabicyclo[3.1.0]hexan-2-one (**5**) (726 mg, 3.18 mmol, 1 eq) was dissolved in dry MeCN (3 mL) and added to the reaction. The flask, which contained the starting material **5**, was washed with dry MeCN (2 × 1 mL) and added to the reaction. After stirring at room temperature for 3 h additional dry MeCN (5 mL) was added. The reaction was stirred at 30 °C for 18 h. The reaction was added to aq. sat. $NaHCO_3$ solution (150 mL) and extracted with EtOAc (3 × 150 mL). The organic phase was dried over sodium sulfate, filtered and volatiles removed under reduced pressure. The crude was purified by column chromatography (cyclohexane/EtOAc, 90:10 to 80:20) to obtain a colorless oil composed of both diastereomers **6** and **7** (1.05 g, 2.77 mmol, 87% over two steps). They were separated by multiple HPLC runs (water/MeCN) to obtain 387 mg (1.02 mmol) of diastereomer **6** and 410 mg (1.08 mmol) of diastereomer **7**.

Diastereomer **6:**

^{1}H NMR (500 MHz, MeCN-d_3): δ = 7.34-7.31 (m, 6H), 7.30-7.27 (m, 2H), 7.20-7.15 (m, 2H), 4.86 (s, 2H), 4.36 (dd, ^{2}J = 9.4 Hz, ^{3}J = 4.8 Hz, 1H), 4.15 (d, ^{2}J = 9.4 Hz, 1H), 2.66 (m_c, 1H), 2.35 (m_c, 2H), 1-67-1.52 (m, 3H), 1.44 (m_c, 2H), 1.05 (m_c, 1H), 0.98 (t, ^{3}J = 7.4 Hz, 3H) ppm.

^{13}C NMR (125 MHz, MeCN-d_3): δ = 175.5, 137.2, 136.7, 129.7, 129.6, 129.6, 129.6, 128.2, 127.8, 109.3, 86.0, 85.9, 68.6, 38.2, 34.7, 27.1, 23.8, 22.8, 15.2, 14.4 ppm.

ESI-HRMS: calc. for $C_{24}H_{26}O_4Na$ [M+Na]$^+$: *m/z* = 401.1723; found: 401.1723

Diastereomer **7:**

^{1}H NMR (500 MHz, MeCN-d_3): δ = 7.38-7.32 (m, 6H), 7.28-7.23 (m, 2H), 7.20-7.15 (m, 2H), 4.83 (d, ^{3}J = 8.8 Hz, 1H), 4.78 (d, ^{3}J = 8.8 Hz, 1H), 4.42 (dd, ^{2}J = 9.4 Hz, ^{3}J = 4.8 Hz, 1H), 4.20 (d, ^{2}J = 9.4 Hz, 1H), 2.55 (m_c, 1H), 2.37 (ddd, ^{2}J = 14.0 Hz, ^{3}J = 11.9 Hz, ^{3}J = 4.7 Hz, 1H), 2.28 (ddd, ^{2}J = 14.0 Hz, ^{3}J = 11.8 Hz, ^{3}J = 5.0 Hz, 1H), 1.71-1.52 (m, 3H), 1.45 (m_c, 2H), 1.06 (m_c, 1H), 0.99 (t, ^{3}J = 7.4 Hz, 3H) ppm.

^{13}C NMR (125 MHz, MeCN-d_3): δ = 175.6, 137.1, 137.1, 129.7, 129.6, 129.6, 129.6, 128.2, 127.8, 109.7, 86.8, 86.0, 68.8, 38.2, 35.2, 26.6, 23.8, 22.6, 16.5, 14.4 ppm.

ESI-HRMS: calc. for $C_{24}H_{26}O_4Na$ [M+Na]$^+$: *m/z* = 401.1723; found: 401.1725

(1*S*,5*R*)-1-pentanoyl-3-oxabicyclo[3.1.0]hexan-2-one (8)

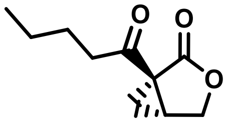

8

(1*S*,5*R*)-1-((4*R*,5*R*)-2-butyl-4,5-diphenyl-1,3-dioxolan-2-yl)-3-oxabicyclo[3.1.0]hexan-2-one (**7**) (397 mg, 1.05 mmol) was placed in a flask and deion. water (1 mL) was added. The flask was cooled to 0 °C. Trifluoroacetic acid (10 mL) was added dropwise and the reaction was stirred for 1 h at 0 °C and 1 h at room temperature. After the TLC showed the absence of the starting material, the reaction was carefully quenched with aq. sat. $NaHCO_3$ solution (10 mL). The mixture was poured into further aq. sat. $NaHCO_3$ solution (150 mL) and extracted with EtOAc (3 × 150 mL). The organic phase was dried over sodium sulfate, filtered and volatiles were removed under reduced pressure. The crude was purified by column chromatography (cyclohexane/EtOAc, 90:10) to yield a colorless oil (165 mg, 906 µmol, 86%).

^{1}H NMR (500 MHz, MeCN-d_3): δ = 4.30 (dd, ^2J = 9.3 Hz, ^3J = 4.8 Hz, 1H), 4.14 (d, ^2J = 9.3 Hz, 1H), 2.99 (ddd, ^2J = 17.7 Hz, ^3J = 8.3 Hz, ^3J = 6.5 Hz, 1H), 2.80 (ddd, ^2J = 17.7 Hz, ^3J = 8.3 Hz, ^3J = 6.4 Hz, 1H), 2.70 (dddd, ^3J = 8.0 Hz, ^3J = 5.6 Hz, ^3J = 4.8 Hz, ^3J = 0.8 Hz, 1H), 1.91 (dd, ^3J = 8.0 Hz, ^2J = 4.3 Hz, 1H), 1.60-1.46 (m, 2H), 1.37 (dd, ^3J = 5.6 Hz, ^2J = 4.3 Hz, 1H), 1.32 (m_c, 2H), 0.90 (t, ^3J = 7.4 Hz, 3H) ppm.

^{13}C NMR (125 MHz, MeCN-d_3): δ = 203.6, 174.3, 68.4, 41.6, 37.1, 30.7, 26.3, 23.6, 22.9, 14.2 ppm.

ESI-HRMS: calc. for $C_{10}H_{14}O_3Na$ $[M+Na]^+$: m/z = 205.0835; found: 205.0837

$[\alpha]_D^{20}$ = −155 (c = 0.733, $CHCl_3$)

(1*R*,5*S*)-1-pentanoyl-3-oxabicyclo[3.1.0]hexan-2-one (9)

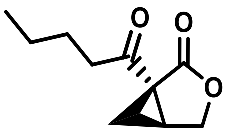

9

(1*R*,5*S*)-1-((4*R*,5*R*)-2-butyl-4,5-diphenyl-1,3-dioxolan-2-yl)-3-oxabicyclo[3.1.0]hexan-2-one (**6**) (360 mg, 951 µmol) was placed in a flask and deion. water (1 mL) was added. The flask was cooled to 0 °C. Trifluoroacetic acid (10 mL) was added dropwise and the reaction was stirred for 1 h at 0 °C and 1 h at room temperature. After the TLC showed the absence of the starting material, the reaction was carefully quenched with aq. sat. $NaHCO_3$ solution (10 mL). The mixture was poured into further aq. sat. $NaHCO_3$ solution (150 mL) and extracted with EtOAc (3 × 150 mL). The organic phase was dried over sodium sulfate, filtered and volatiles were removed under reduced pressure. The crude was purified by column chromatography (cyclohexane/EtOAc, 90:10) to yield a colorless oil (153 mg, 840 µmol, 88%).

^{1}H NMR (500 MHz, MeCN-d_3): δ = 4.30 (dd, ^2J = 9.3 Hz, ^3J = 4.8 Hz, 1H), 4.14 (d, ^2J = 9.3 Hz, 1H), 2.99 (ddd, ^2J = 17.7 Hz, ^3J = 8.3 Hz, ^3J = 6.5 Hz, 1H), 2.80 (ddd, ^2J = 17.7 Hz, ^3J = 8.3 Hz, ^3J = 6.4 Hz, 1H), 2.70 (dddd, ^3J = 8.0 Hz, ^3J = 5.6 Hz, ^3J = 4.8 Hz, ^3J = 0.8 Hz, 1H), 1.91 (dd, ^3J = 8.0 Hz, ^2J = 4.3 Hz, 1H), 1.60-1.46 (m, 2H), 1.37 (dd, ^3J = 5.6 Hz, ^2J = 4.3 Hz, 1H), 1.32 (m_c, 2H), 0.90 (t, ^3J = 7.4 Hz, 3H) ppm.

^{13}C NMR (125 MHz, MeCN-d_3): δ = 203.6, 174.3, 68.4, 41.6, 37.1, 30.7, 26.3, 23.6, 22.9, 14.2 ppm.

ESI-HRMS: calc. for $C_{10}H_{14}O_3Na$ $[M+Na]^+$: m/z = 205.0835; found: 205.0835

$[\alpha]_D^{20}$ = 139 (c = 0.751, $CHCl_3$)

Cysteine opened salinilactone 11

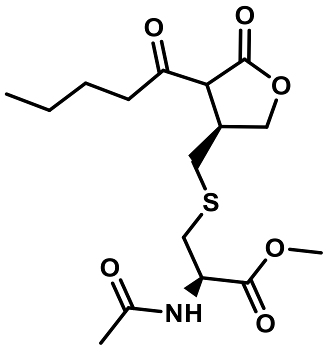

11

(1*R*,5*S*)-1-pentanoyl-3-oxabicyclo[3.1.0]hexan-2-one (**9**) (18.9 mg, 104 µmol, 1 eq.) and *N*-acetyl-L-cysteine methyl ester (**10**) (64.3 mg, 363 µmol, 3.5 eq.) were dissolved in dry MeCN (1.00 mL) under argon atmosphere. 1,8-Diazabicyclo[5.4.0]undec-7-ene (50 µL, 51 mg, 335 µmol, 3.2 eq.) was added to the solution. The reaction was stirred at room temperature for 3 h. After LC-MS did not show any remaining starting material **9**, the solution was poured into 1 M aqueous HCl solution (20 mL). It was extracted with ethyl acetate (3 × 50 mL). The organic phase was dried over sodium sulfate, filtered and volatiles were removed under reduced pressure. The crude was purified by HPLC (water/MeCN) to obtain the product as a colorless oil (39.5 mg, 110 µmol, quant.).

^{1}H NMR (700 MHz, MeCN-d_3): δ = 6.82 (d, ^3J = 6.2 Hz, 1H), 4.57 (m_c, 1H), 4.42 (m_c, 1H), 3.98 (m_c, 1H), 3.71-3.69 (m, 1H), 3.69 (s, 3H), 3.21 (m_c, 1H), 2.93 (dd, ^2J = 13.8 Hz, ^3J = 5.3 Hz, 1H), 2.86-2.80 (m, 2H), 2.73-2.62 (m, 3H), 1.92 (s, 3H), 1.55 (quin, ^3J = 7.5 Hz, 2H), 1.32 (m_c, 2H), 0.90 (t, ^3J = 7.4 Hz, 3H) ppm.

^{13}C NMR (175 MHz, MeCN-d_3): δ = 204.5, 173.6, 172.1, 170.7, 71.7, 58.3, 53.0, 52.9, 43.3, 38.5, 34.5, 34.1, 26.0, 22.7, 22.7, 14.1 ppm.

ESI-HRMS: calc. for $C_{16}H_{26}NO_6S$ $[M+H]^+$: m/z = 360.1475; found: 360.1475

Hept-6-enoyl chloride (14)

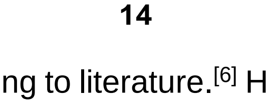

14

This synthesis was carried out according to literature.[6] Hept-6-enoic acid (**13**) (2.00 g, 2.11 mL, 15.6 mmol, 1 eq.) was dissolved in dry *n*-hexane (100 mL) under argon atmosphere. Oxalyl chloride (4.18 g, 2.70 mL, 32.9 mmol, 2 eq.) was added dropwise to the solution. The reaction was stirred at room temperature for 24 h. Volatiles were removed under reduced pressure (max. 10 min at 30 mbar). The colorless oil was stored under argon and used without purification.

Allyl 3-oxonon-8-enoate (16)

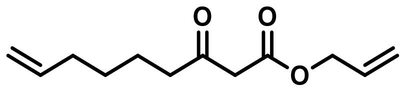

16

Allyl acetate (**15**) (1.72 g, 1.85 mL, 17.2 mmol, 1.1 eq.) was dissolved in dry tetrahydrofuran (10 mL) under argon atmosphere and cooled to −78 °C. A 1 M solution of lithium bis(trimethylsily)amide in tetrahydrofuran (34.0 mL, 34.0 mmol, 2.2 eq.) was added dropwise to the solution. The reaction was stirred for 1 h at −78 °C. Hept-6-enoyl chloride (**14**) (15.6 mmol, 1 eq.) was added via a syringe. The flask, which contained the acid chloride, was washed with dry tetrahydrofuran (3 mL) and added to the reaction with the same syringe. The reaction was stirred for 30 min at −78 °C. Afterwards it was warmed up to room temperature and stirred for 2 h. The reaction was quenched with an aq. 10% HCl solution (7 mL) under ice cooling. Deion. water (24 mL) was added to the flask. After separating the phases, the aqueous phase was extracted with methyl *tert*-butyl ether (2 × 80 mL). The combined organic phases were washed with aq. 10% HCl solution (1 × 50 mL), with aq. sat. $NaHCO_3$ solution (1 × 80 mL) and with aq. sat. NaCl solution (1 × 80 mL). The organic phase was dried over sodium sulfate, filtered and volatiles removed under reduced pressure. The crude was purified by column chromatography (cyclohexane/EtOAc, 96:4 to 93:7). The product was obtained as a yellow oil (2.46 g, 11.7 mmol, 75% over two steps).

^{1}H NMR (500 MHz, MeCN-d_3): δ = 5.93 (ddt, ^3J = 17.2 Hz, ^3J = 10.6 Hz, ^3J = 5.6 Hz, 1H), 5.82 (ddt, ^3J = 17.1 Hz, ^3J = 10.3 Hz, ^3J = 6.7 Hz, 1H), 5.33 (dq, ^3J = 17.3 Hz, $^{2/4}J$ = 1.6 Hz, 1H), 5.22 (dq, ^3J = 10.5 Hz, $^{2/4}J$ = 1.4 Hz, 1H), 5.01 (m_c, 1H), 4.94 (m_c, 1H), 4.59 (dt, ^3J = 5.6 Hz, ^4J = 1.5 Hz, 1H), 3.49 (s, 2H), 2.53 (t, ^3J = 7.3 Hz, 2H), 2.04 (m_c, 2H), 1.57-1.50 (m, 2H), 1.40-1.32 (m, 2H) ppm.

^{13}C NMR (125 MHz, MeCN-d_3): δ = 204.4, 168.1, 139.7, 133.3, 118.4, 115.0, 66.2, 49.7, 43.3, 34.1, 28.9, 23.5 ppm.

ESI-HRMS: calc. for $C_{12}H_{18}O_3Na$ [M+Na]$^+$: m/z = 233.1148; found: 233.1149

Allyl 2-diazo-3-oxonon-8-enoate (17)

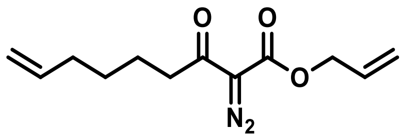

17

Allyl 3-oxonon-8-enoate (**16**) (2.46 g, 11.7 mmol, 1 eq.) was dissolved in dry acetonitrile (24 mL). 4-Acetamidobenzenesulfonyl azide (2.81 g, 11.7 mmol, 1 eq.) was added to the solution. The atmosphere inside the flask was exchanged against argon. The flask was cooled to 0 °C and triethylamine (3.55 g, 4.90 mL, 35.1 mmol, 3 eq) was added dropwise. The reaction was stirred at 0 °C for 20 min and afterwards for 24 h at room temperature in the dark. Volatiles were removed under reduced pressure. The residue was suspended in 1:1 mixture of pentane and diethylether (30 mL). The suspension was filtered and the solid washed with the same mixture (3 × 30 mL). The solvent of the filtrate was removed under reduced pressure. The crude was purified by column chromatography using basic aluminum oxide as solid phase (cyclohexane/methyl *tert*-butyl ether, 90:10 to 85:15) to yield a yellow oil (2.37 g, 10.0 mmol, 85%).*

*Purification may be carried out with silica gel or neutral aluminum oxide as solid phase since the decomposition of the product was observed when using a new batch of the basic aluminum oxide. With an old batch, no decomposition was observed.

^{1}H NMR (500 MHz, MeCN-d_3): δ = 5.98 (ddt, ^{3}J = 17.2 Hz, ^{3}J = 10.8 Hz, ^{3}J = 5.5 Hz, 1H), 5.83 (ddt, ^{3}J = 17.0 Hz, ^{3}J = 10.2 Hz, ^{3}J = 6.7 Hz, 1H), 5.35 (dq, ^{3}J = 17.3 Hz, $^{2/4}J$ = 1.6 Hz, 1H), 5.26 (dq, ^{3}J = 10.5 Hz, $^{2/4}J$ = 1.3 Hz, 1H), 5.01 (dq, ^{3}J = 17.2 Hz, $^{2/4}J$ = 1.8 Hz, 1H), 4.94 (m_c, 1H), 4.71 (dt, ^{3}J = 5.5 Hz, ^{4}J = 1.5 Hz, 2H), 2.80 (t, ^{3}J = 7.4 Hz, 2H), 2.09 (m_c, 2H), 1.63-1.55 (m, 2H), 1.44-1.37 (m, 2H) ppm.

^{13}C NMR (125 MHz, DMSO-d_6): δ = 191.7, 160.6, 138.5, 132.2, 118.2, 114.9, 75.2, 65.2, 39.2*, 33.0, 27.7, 23.3 ppm.

*Assigned via HMBC

ESI-HRMS: calc. for $C_{12}H_{16}N_2O_3Na$ [M+Na]$^+$: m/z = 259.1053; found: 259.1054

1-(Hept-6-enoyl)-3-oxabicyclo[3.1.0]hexan-2-one (18)

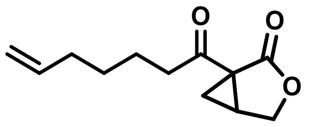

18

Cu(tbs)$_2$ **25** (213 mg, 512 µmol, 0.1 eq.) was dissolved in dry toluene (5 mL) under argon atmosphere and heated to reflux. Allyl 2-diazo-3-oxonon-8-enoate (**17**) (1.21 g, 5.12 mmol, 1 eq.) was dissolved in dry toluene (20 mL) and added dropwise over a period of 1 h to the copper(II) solution. The flask which contained the diazo compound **17** was washed with dry toluene (2 mL) and added to the reaction with the same syringe. The reaction was stirred for 2 h at reflux. After the TLC showed the consumption of the starting material, the reaction was cooled to room temperature and volatiles were removed under reduced pressure. The crude was purified by column chromatography (cyclohexane/EtOAc, 80:20 to 70:30) to yield a colorless oil (685 mg, 3.29 mmol, 64%).

^{1}H NMR (500 MHz, MeCN-d_3): δ = 5.83 (ddt, ^3J = 17.0 Hz, ^3J = 10.3 Hz, ^3J = 6.7 Hz, 1H), 5.02 (m$_c$, 1H), 4.94 (m$_c$, 1H), 4.30 (dd, ^2J = 9.3 Hz, ^3J = 4.8 Hz, 1H), 4.14 (d, ^2J = 9.3 Hz, 1H), 3.00 (ddd, ^2J = 17.8 Hz, ^3J = 8.0 Hz, ^3J = 6.6 Hz, 1H), 2.80 (ddd, ^2J = 17.8 Hz, ^3J = 8.0 Hz, ^3J = 6.5 Hz, 1H), 2.70 (dddd, ^3J = 8.0 Hz, ^3J = 5.7 Hz, ^3J = 4.8 Hz, ^3J = 0.8 Hz, 1H), 2.06 (m$_c$, 2H), 1.92 (dd, ^3J = 8.0 Hz, ^2J = 4.2 Hz, 1H), 1.61-1.50 (m, 2H), 1.42-1.35 (m, 3H) ppm.

^{13}C NMR (125 MHz, MeCN-d_3): δ = 203.5, 174.3, 139.9, 128.9, 115.1, 68.4, 41.7, 37.1, 34.3, 30.7, 29.1, 23.7 ppm.

ESI-HRMS: calc. for $C_{12}H_{16}O_3Na$ [M+Na]$^+$: m/z = 231.0992; found: 231.0992

[1-[(3,5-Dimethyl-1*H*-pyrrol-2-yl)(3,5-dimethyl-2*H*-pyrrol-2-ylidene)methyl]-4-iodobenzene](difluoroborane) (21)

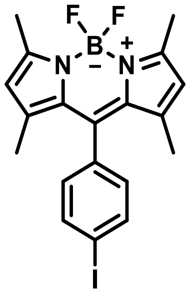

21

The BODIPY derivative **21** was synthesized according to literature.[7] 4-Iodobenzoyl chloride (**19**) (2.65 g, 9.95 mmol, 1 eq.) and 2,4-dimethylpyrrole (**20**) (2.77 g, 3.00 mL, 29.1 mmol, 3 eq.) were combined under argon atmosphere and reacted for 6 h at room temperature. Triethylamine (2.92 g, 4.00 mL, 28.9 mmol, 3 eq.) was added and reacted for 30 min at room temperature. Boron trifluoride etherate (10.4 g, 9.00 mL, 73.3 mmol, 7.4 eq.) was added and the reaction shaken for 18 h on an orbital shaker at room temperature. Volatiles were removed under reduced pressure. The crude was purified by column chromatography (petroleum ether/CH_2Cl_2, 6:1) yielding an orange solid (1.07 g, 2.38 mmol, 24%).

^{1}H NMR (500 MHz, MeCN-d_3): δ = 7.91 (d, ^{3}J = 8.3 Hz, 2H), 7.15 (d, ^{3}J = 8.3 Hz, 2H), 6.10 (s, 2H), 2.48 (s, 6H), 1.42 (s, 6H) ppm.

^{13}C NMR (125 MHz, MeCN-d_3): δ = 156.7, 144.4, 141.8, 139.3, 135.2, 131.9, 131.2, 122.4, 95.4, 14.8, 14.7 ppm.

ESI-HRMS: calc. for $C_{19}H_{19}BF_2IN_2$ $[M+H]^+$: m/z = 451.0649; found: 451.0648

Unsaturated BODIPY-salinilactone conjugate 22

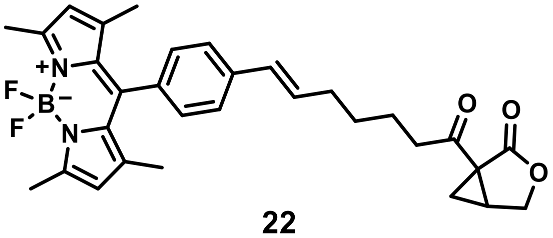

22

1-(Hept-6-enoyl)-3-oxabicyclo[3.1.0]hexan-2-one (**18**) (42.4 mg, 204 µmol, 1 eq.), [1-[(3,5-Dimethyl-1*H*-pyrrol-2-yl)(3,5-dimethyl-2*H*-pyrrol-2-ylidene)methyl]-4-iodobenzene](difluoro-borane) (**21**) (187 mg, 415 µmol, 2 eq.), bis(tri-*tert*-butylphosphine)palladium(0) (31.4 mg, 61.4 µmol, 0.3 eq.) and tetrabutylammonium chloride (18.0 mg, 64.8 µmol, 0.3 eq.) were dissolved in dry dimethylacetamide (2 mL) under argon atmosphere. *N*,*N*-dicyclohexylmethylamine (199 mg, 218 µL, 1.02 mmol, 5 eq.) was added and the reaction was stirred 21 h at 60 °C and 2 h at 80 °C. The mixture was taken up in EtOAc (100 mL) and washed with aq. sat. NaCl solution (3 × 80 mL). The organic phase was dried over sodium sulfate, filtered and volatiles were removed under reduced pressure. The crude was purified by column chromatography (cyclohexane/EtOAc, 90:10 to 70:30) to yield a mixture of three isomers* as a red film (52.3 mg, 98.6 µmol, 47%).

*A mixture of three isomers was obtained, which were identified as the *cis*- and *trans*-diastereomers and a regioisomer with a terminal double bond, resulting from a regioisomeric addition of the Pd-arene to the olefin of **18**.[8] The branched byproduct was removed by HPLC after the next reducing step.

^{1}H and ^{13}C NMR were to complex to assign the signals. Nevertheless, the signals for the *trans* double bond and the signals of the regioisomer with a terminal double bond could be identified. The spectra are displayed in chapter 8.

ESI-HRMS: calc. for $C_{31}H_{34}BF_2N_2O_3$ $[M+H]^+$: m/z = 531.2625; found: 531.2624

Table S4. Tested reaction conditions for the Heck reaction.

Catalyst	Base	Additive	Reaction Condition	Outcome
Pd-PEPPSI™-*i*-Pent	Et_3N	$Bu_4N^+I^-$	DMF, 120 °C, 8 h	poor conversion
$PdCl_2(P(cy)_3)_2$	Cs_2CO_3	-	1,4-dioaxane, 80 °C, 16 h	no product in NMR
$Pd_2(dba)_3$ + $Pd(t\text{-}Bu_3P)_2$	$(C_6H_{11})_2NCH_3$	-	1,4-dioxane, reflux, 24 h	no product in NMR
$Pd(t\text{-}Bu_3P)_2$	$(C_6H_{11})_2NCH_3$	-	1,4-dioxane, RT, 18 h	poor conversion
$Pd(t\text{-}Bu_3P)_2$	$(C_6H_{11})_2NCH_3$	$Bu_4N^+Cl^{-*}$	DMA, 60 °C, 21 h, 80 °C, 2 h	47%

***** The quaternary amine chloride was important, as it served to increase the catalytic activity of the Pd(0) species by coordination of the chloride anion.[9]

Reduced BODIPY-salinilactone conjugate 23

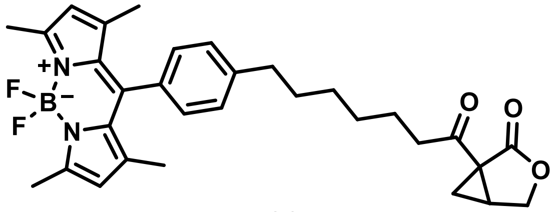

23

The unsaturated BODIPY-salinilactone conjugate **22** (54.2 mg, 102 µmol, 1 eq.) and 10% palladium on activated charcoal (10.5 mg, 9.87 µmol, 0.1 eq.) were placed in a flask and the atmosphere was exchanged against hydrogen. Dry EtOAc (2 mL) was added and the reaction was stirred at room temperature for 5 h. The palladium catalyst was removed by filtration through a pad of Celite® 535 under reduced pressure. The pad was washed with EtOAc. Volatiles were removed under reduced pressure. The crude was purified by HPLC (water/MeCN). The product was obtained as a red film (31.8 mg, 59.7 µmol, 60%).

^{1}H NMR (500 MHz, MeCN-d_3): δ = 7.36 (d, ^{3}J = 8.1 Hz, 2H), 7.22 (d, ^{3}J = 8.1 Hz, 1H), 6.08 (d, 2H), 4.29 (dd, ^{2}J = 9.3 Hz, ^{3}J = 4.8 Hz, 1H), 4.14 (d, ^{2}J = 9.3 Hz, 1H), 2.98 (ddd, ^{2}J = 17.7 Hz, ^{3}J = 8.3 Hz, ^{3}J = 6.4 Hz, 1H), 2.78 (ddd, ^{2}J = 17.7 Hz, ^{3}J = 8.3 Hz, ^{3}J = 6.3 Hz, 1H), 2.73-2.66 (m, 3H), 2.47 (s, 6H), 1.91 (dd, ^{3}J = 8.0 Hz, ^{2}J = 4.3 Hz, 1H), 1.69-1.60 (m, 2H), 1.58-1.48 (m, 2H), 1.29 (s, 6H), 1.37 (dd, ^{3}J = 5.7 Hz, ^{2}J = 4.3 Hz, 1H), 1.35-1.30 (m, 4H) ppm.

^{13}C NMR (125 MHz, MeCN-d_3): δ = 203.6, 174.3, 156.2, 145.2, 144.6, 143.9, 132.9, 132.4, 130.3, 128.8, 122.2, 68.4, 41.8, 37.1, 36.1, 32.1, 30.7, 29.5, 29.5, 24.1, 23.7, 14.8, 14.6 ppm.

ESI-HRMS: calc. for $C_{31}H_{36}BF_2N_2O_3$ $[M+H]^+$: m/z = 533.2782; found: 533.2783

Bis(*N*-*tert*-butylsalicylideneiminate)copper(II) (Cu(tbs)$_2$) (25)

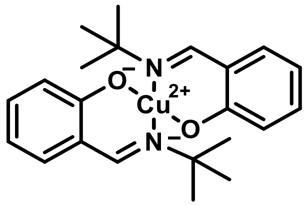

25

Cu(tbs)$_2$ was synthesized according to literature.[10] Copper(II) acetate monohydrate (399 mg, 2.00 mmol, 1 eq.) was dissolved in deion. water (5 ml). Salicylic aldehyde (**24**) (501 mg, 4.10 mmol, 2.1 eq.) was dissolved in EtOH (2 mL) and added to the copper(II) solution. The reaction was stirred at 55 °C for 1 h. The reaction was filtered and the precipitate suspended in EtOH (2 mL). *Tert*-butylamine (366 mg, 526 µL, 5.00 mmol, 2.5 eq.) was added to the suspension and stirred under reflux for 1.5 h. Volatiles were removed under reduced pressure. The product was used without purification yielding a black solid (722 mg, 1.74 mmol, 87%).

10. NMR spectra

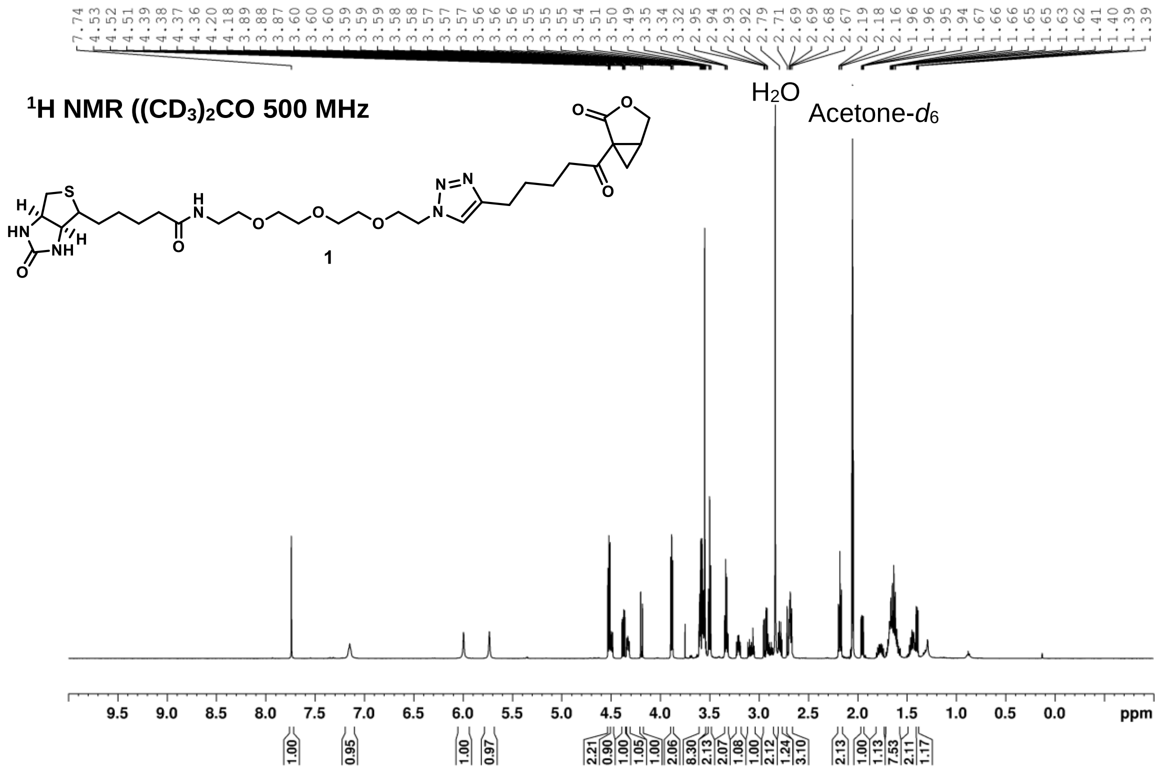

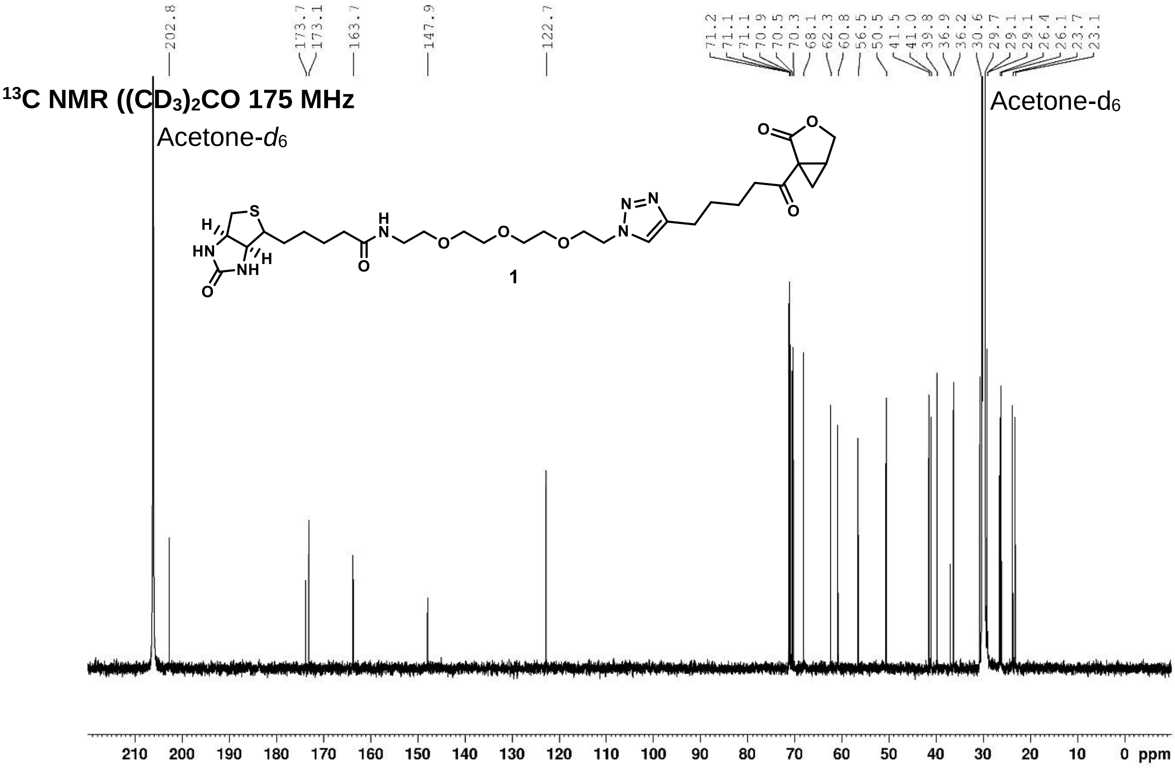

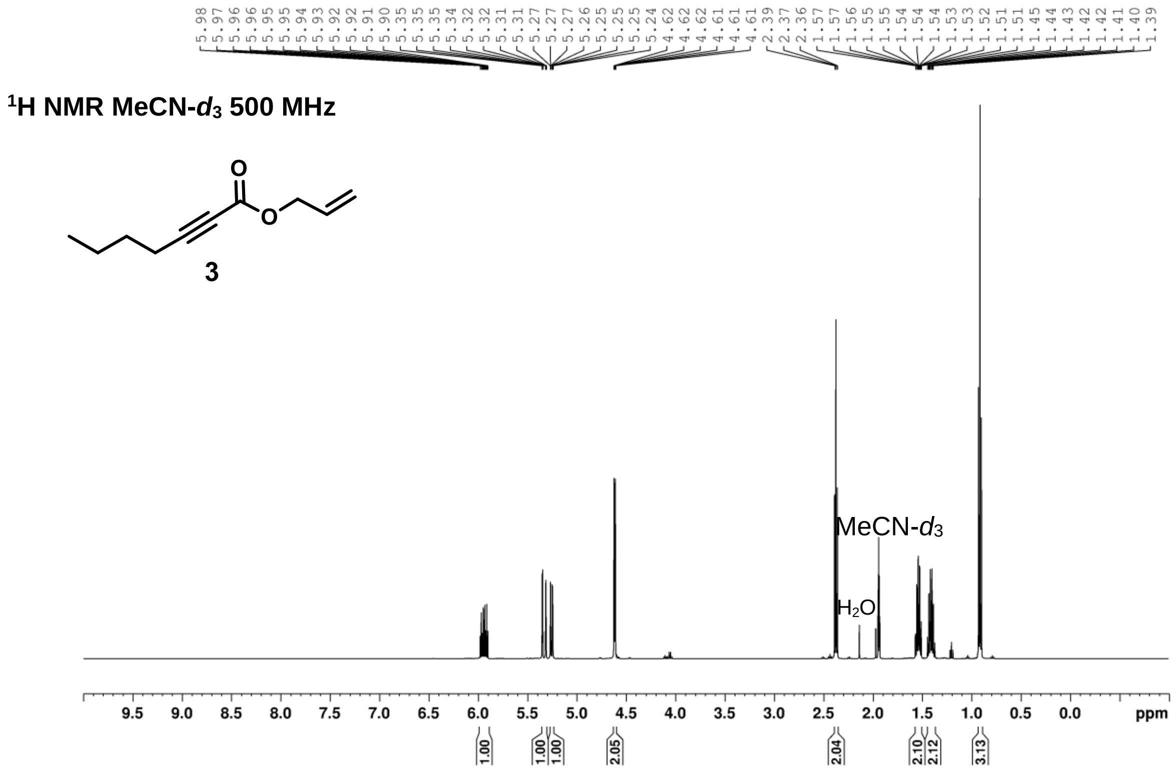
1H NMR MeCN-d3 500 MHz
5.98 5.97 5.96 5.96 5.95 5.95 5.94 5.93 5.92 5.92 5.92 5.91 5.90 5.35 5.35 5.35 5.34 5.32 5.32 5.31 5.31 5.27 5.27 5.27 5.26 5.25 5.25 5.25 5.24 4.62 4.62 4.62 4.62 4.61 4.61 4.61 2.39 2.37 2.36 1.57 1.57 1.56 1.55 1.55 1.54 1.54 1.54 1.53 1.53 1.52 1.51 1.51 1.45 1.44 1.43 1.42 1.42 1.41 1.40 1.39
O
O
3
MeCN-d3
H2O
9.5 9.0 8.5 8.0 7.5 7.0 6.5 6.0 5.5 5.0 4.5 4.0 3.5 3.0 2.5 2.0 1.5 1.0 0.5 0.0 ppm
1.00 1.00 1.00 2.05 2.04 2.10 2.12 3.13

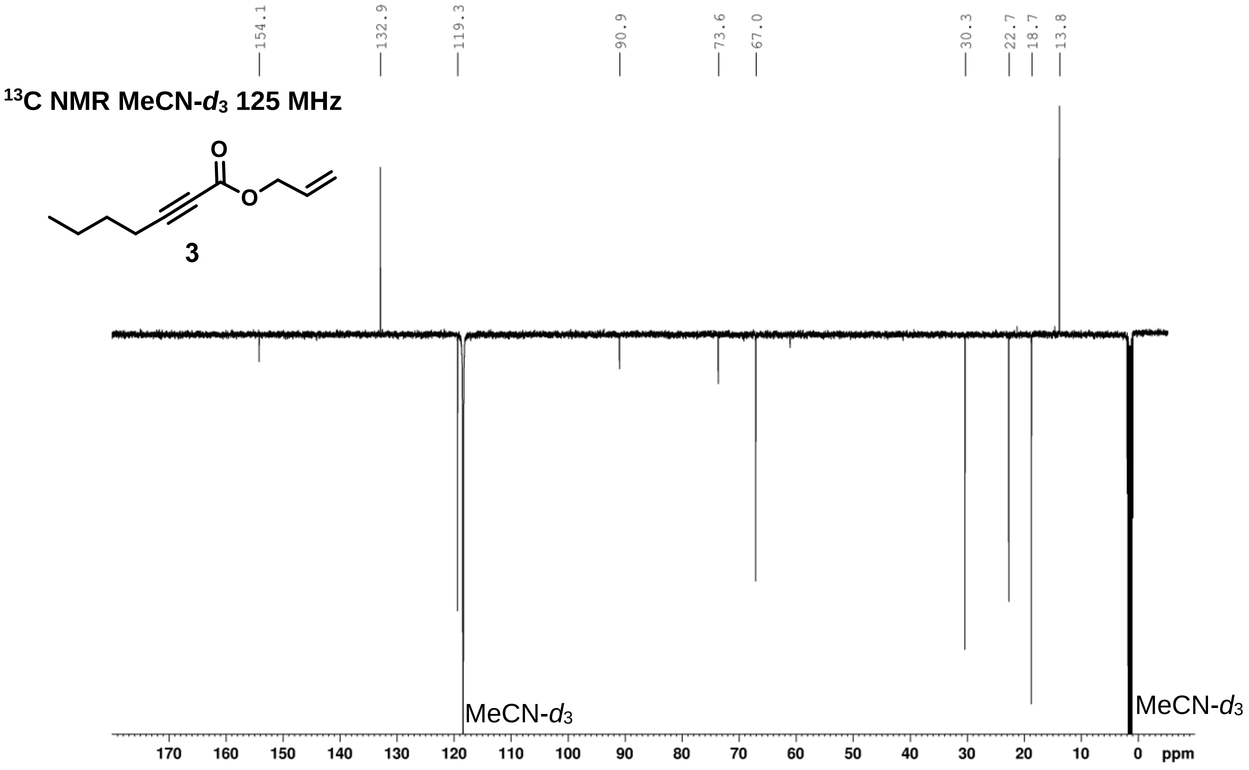
154.1 132.9 119.3 90.9 73.6 67.0 30.3 22.7 18.7 13.8
13C NMR MeCN-d3 125 MHz
O
O
3
MeCN-d3
MeCN-d3
170 160 150 140 130 120 110 100 90 80 70 60 50 40 30 20 10 0 ppm

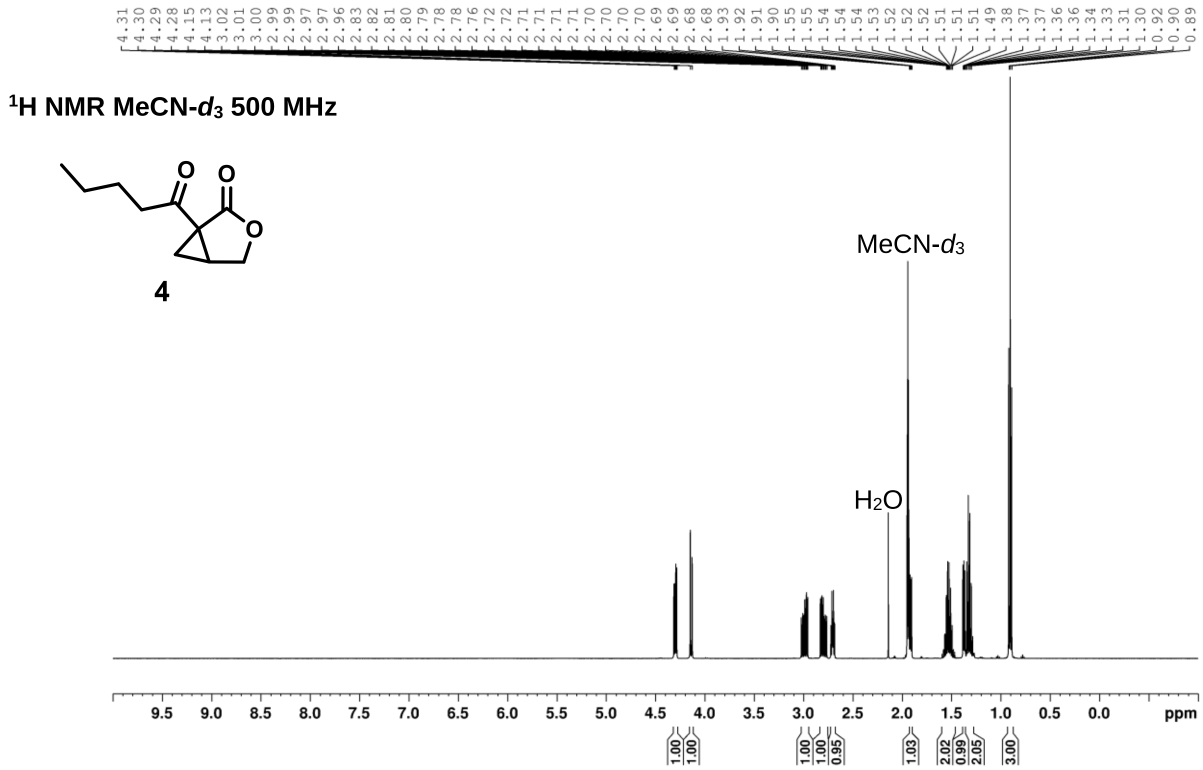
4.31
4.30
4.29
4.28
4.15
4.13
3.02
3.01
3.00
2.99
2.99
2.97
2.97
2.96
2.83
2.82
2.81
2.80
2.79
2.79
2.78
2.78
2.76
2.72
2.72
2.71
2.71
2.71
2.71
2.70
2.70
2.70
2.70
2.69
2.69
2.68
1.93
1.92
1.92
1.91
1.90
1.55
1.55
1.54
1.54
1.54
1.53
1.52
1.52
1.52
1.51
1.51
1.51
1.49
1.38
1.37
1.37
1.36
1.34
1.33
1.31
1.30
0.92
0.90
0.89
1H NMR MeCN-d3 500 MHz
O
O
O
4
MeCN-d3
H2O
9.5 9.0 8.5 8.0 7.5 7.0 6.5 6.0 5.5 5.0 4.5 4.0 3.5 3.0 2.5 2.0 1.5 1.0 0.5 0.0 ppm
1.00
1.00
1.00
1.00
0.95
1.03
2.02
0.99
2.05
3.00

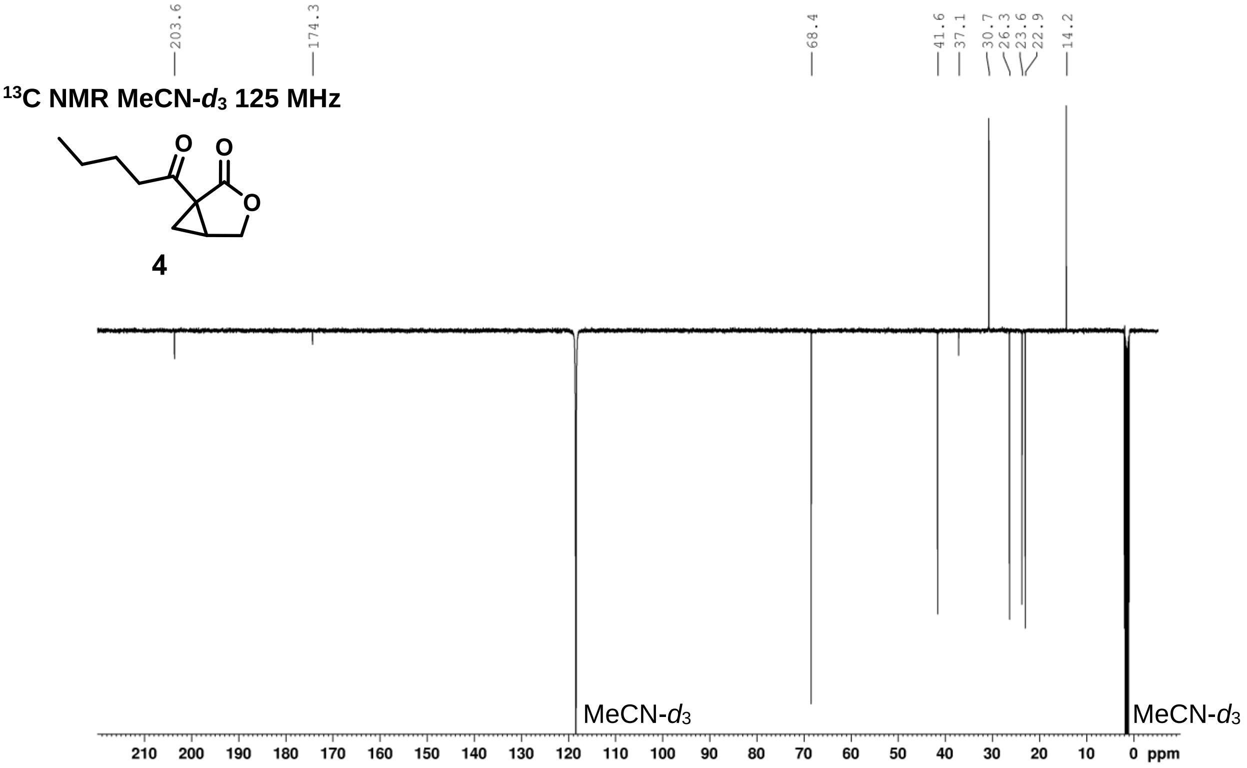
203.6
174.3
68.4
41.6
37.1
30.7
26.3
23.6
22.9
14.2
13C NMR MeCN-d3 125 MHz
O
O
O
4
MeCN-d3
MeCN-d3
210 200 190 180 170 160 150 140 130 120 110 100 90 80 70 60 50 40 30 20 10 0 ppm

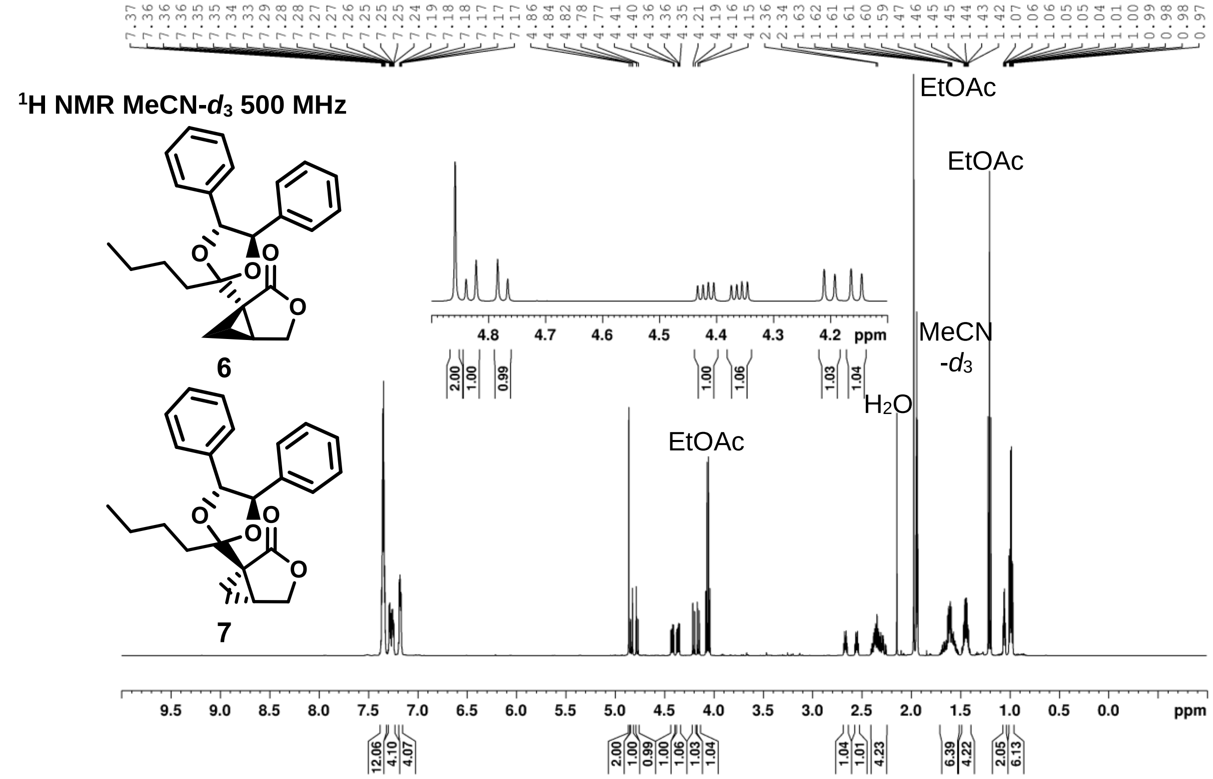
1H NMR MeCN-d3 500 MHz
6
7
EtOAc
EtOAc
EtOAc
MeCN
-d3
H2O
ppm
ppm

^{1}H NMR MeCN-d_3 500 MHz

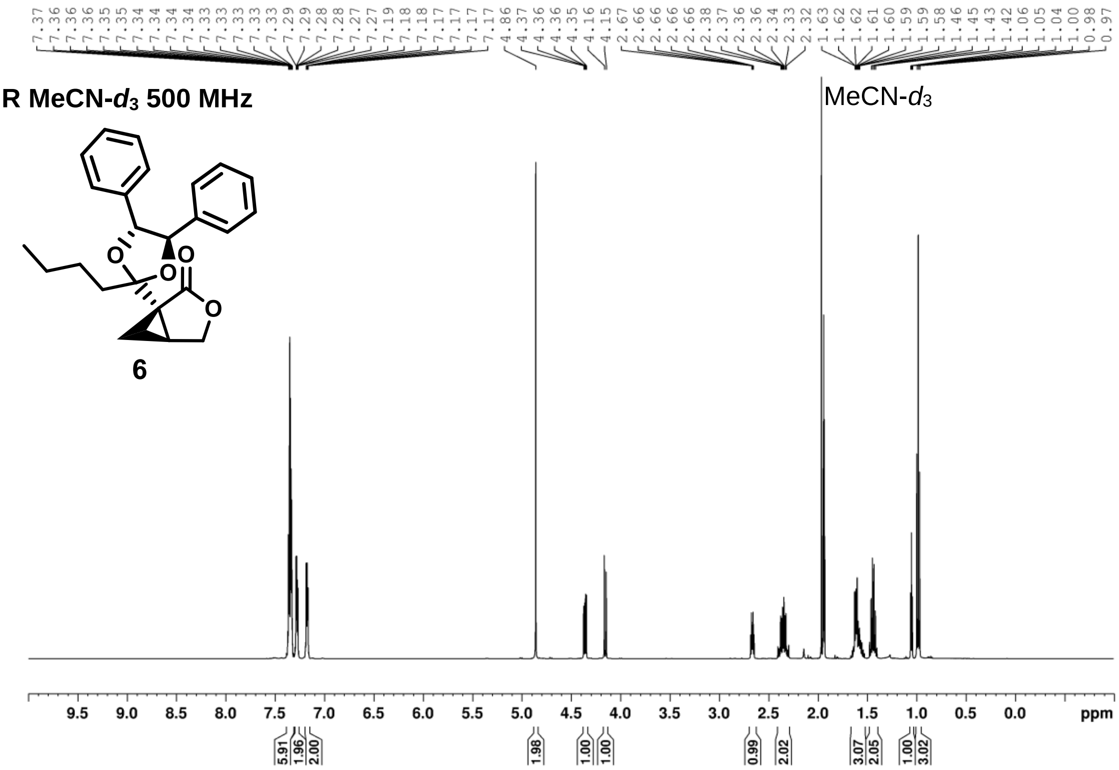

^{13}C NMR MeCN-d_3 125 MHz

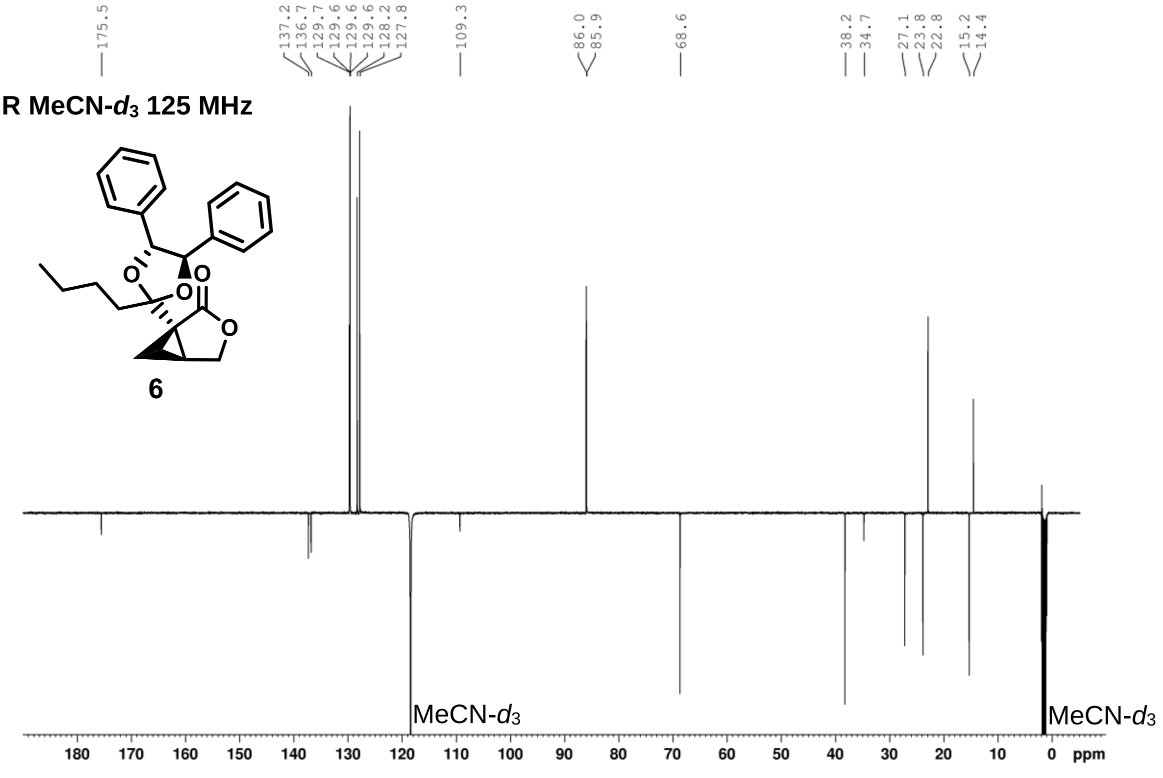

^{1}H NMR MeCN-d_3 500 MHz

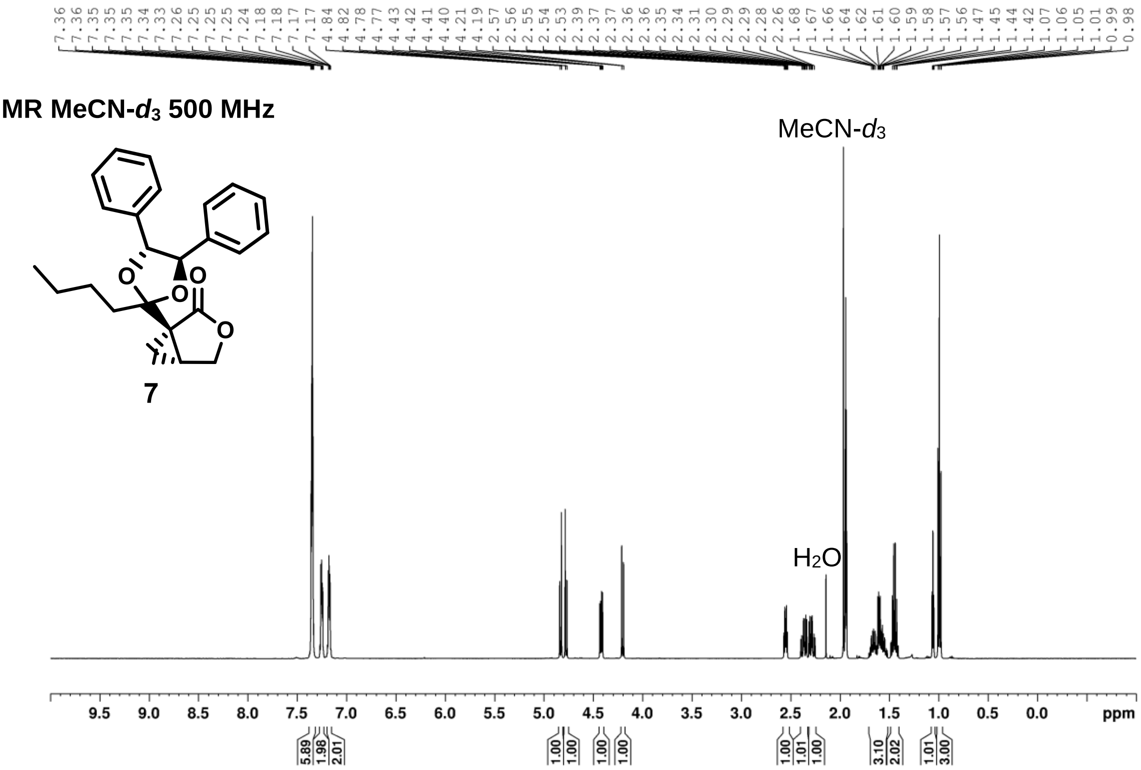

^{13}C NMR MeCN-d_3 125 MHz

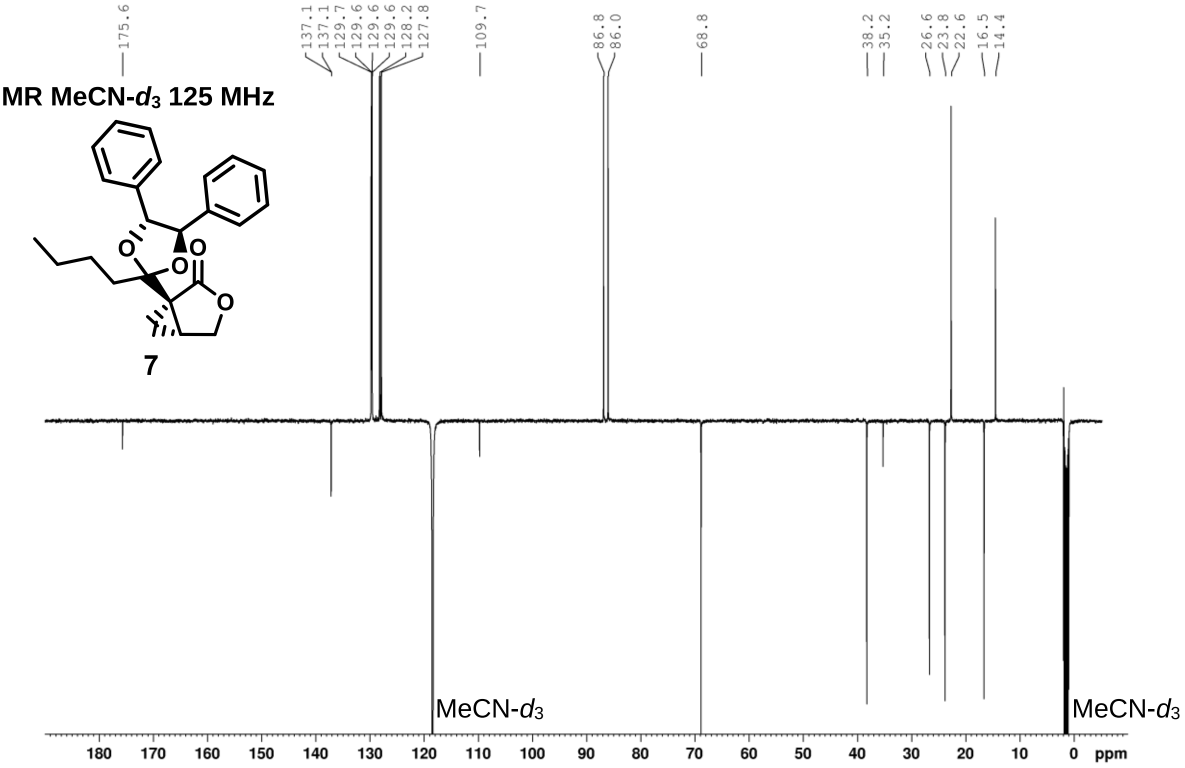

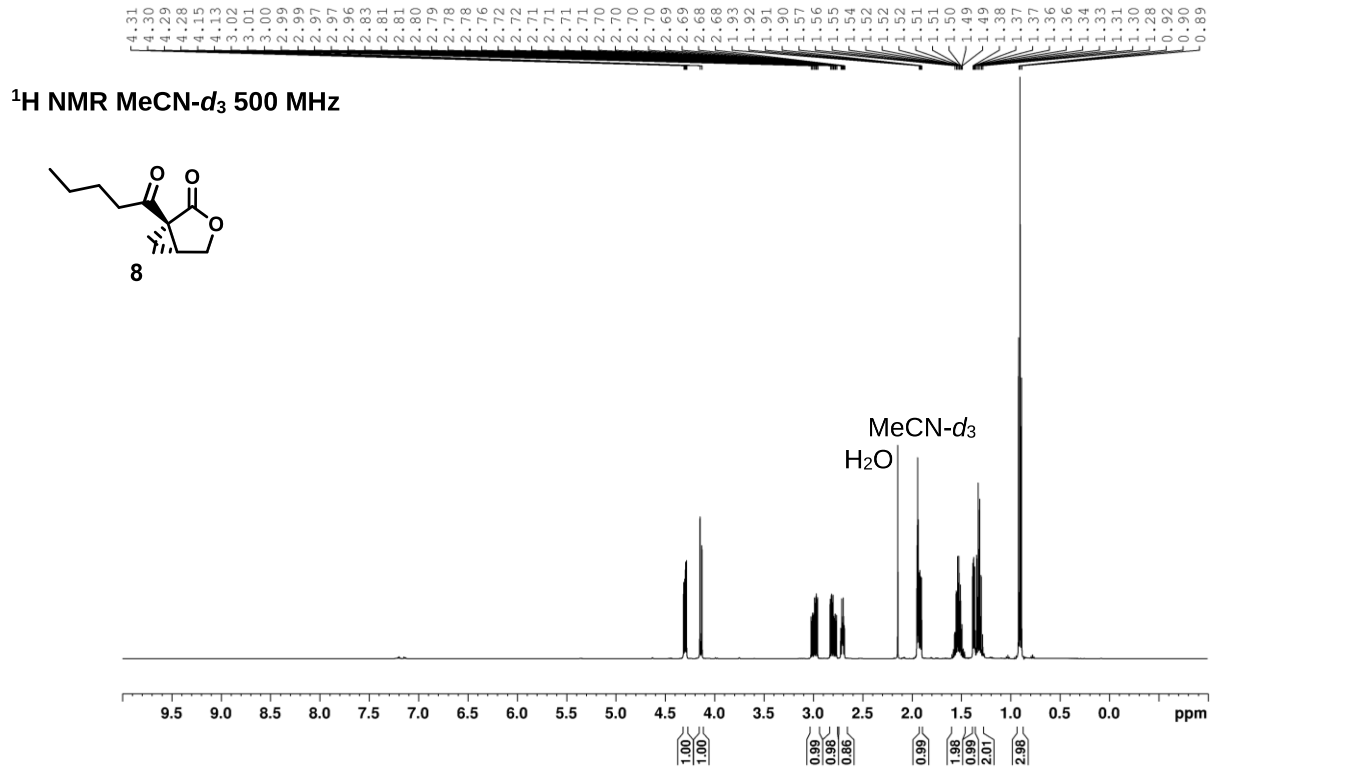
4.31
4.30
4.29
4.28
4.15
4.13
3.02
3.01
3.00
2.99
2.99
2.97
2.97
2.96
2.83
2.81
2.81
2.80
2.79
2.78
2.78
2.76
2.72
2.72
2.72
2.71
2.71
2.71
2.71
2.70
2.70
2.70
2.70
2.69
2.69
2.68
1.93
1.92
1.91
1.90
1.57
1.56
1.55
1.54
1.52
1.52
1.52
1.51
1.51
1.50
1.49
1.49
1.38
1.37
1.37
1.36
1.36
1.34
1.33
1.31
1.30
1.28
0.92
0.90
0.89
1H NMR MeCN-d3 500 MHz
8
MeCN-d3
H2O
9.5 9.0 8.5 8.0 7.5 7.0 6.5 6.0 5.5 5.0 4.5 4.0 3.5 3.0 2.5 2.0 1.5 1.0 0.5 0.0 ppm
1.00 1.00 0.99 0.98 0.86 0.99 1.98 0.99 2.01 2.98

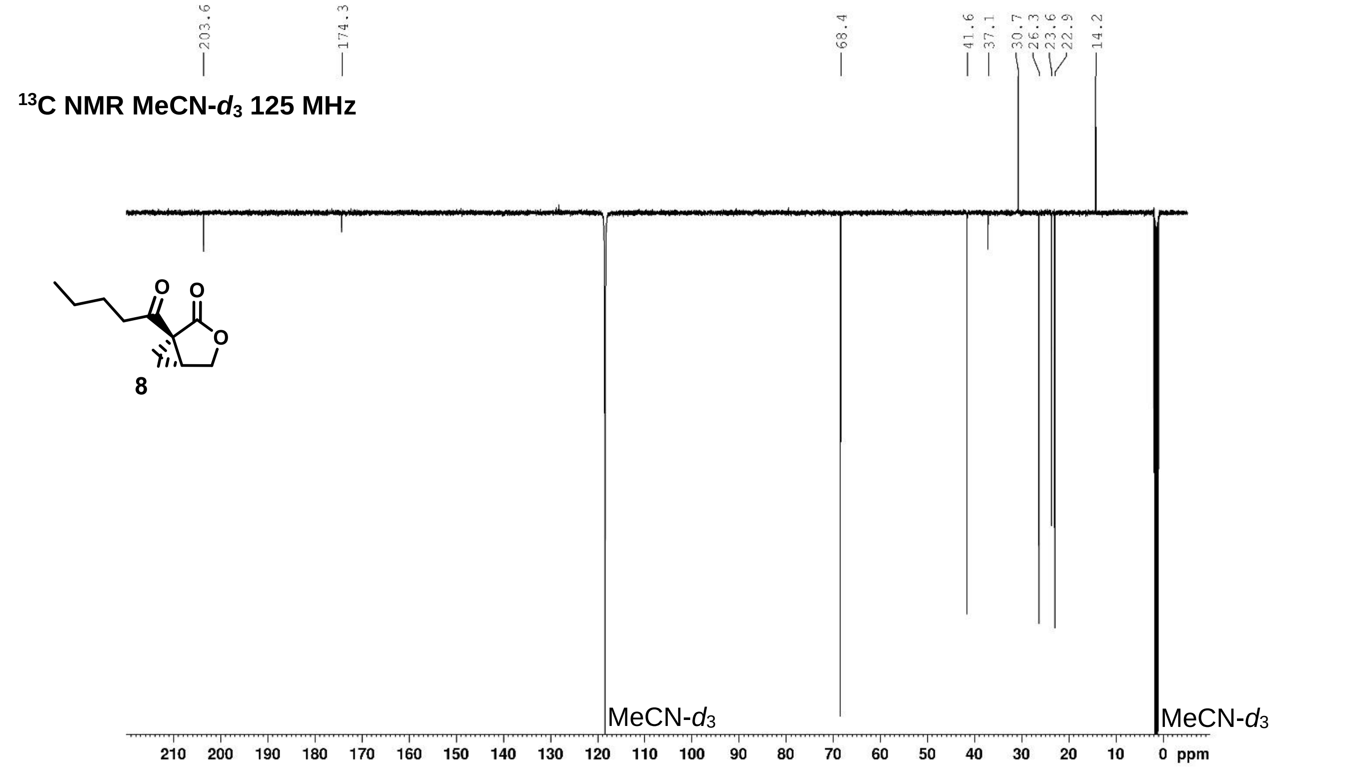
203.6
174.3
68.4
41.6
37.1
30.7
26.3
23.6
22.9
14.2
13C NMR MeCN-d3 125 MHz
8
MeCN-d3
MeCN-d3
210 200 190 180 170 160 150 140 130 120 110 100 90 80 70 60 50 40 30 20 10 0 ppm

^{1}H NMR MeCN-*d*$_3$ 500 MHz

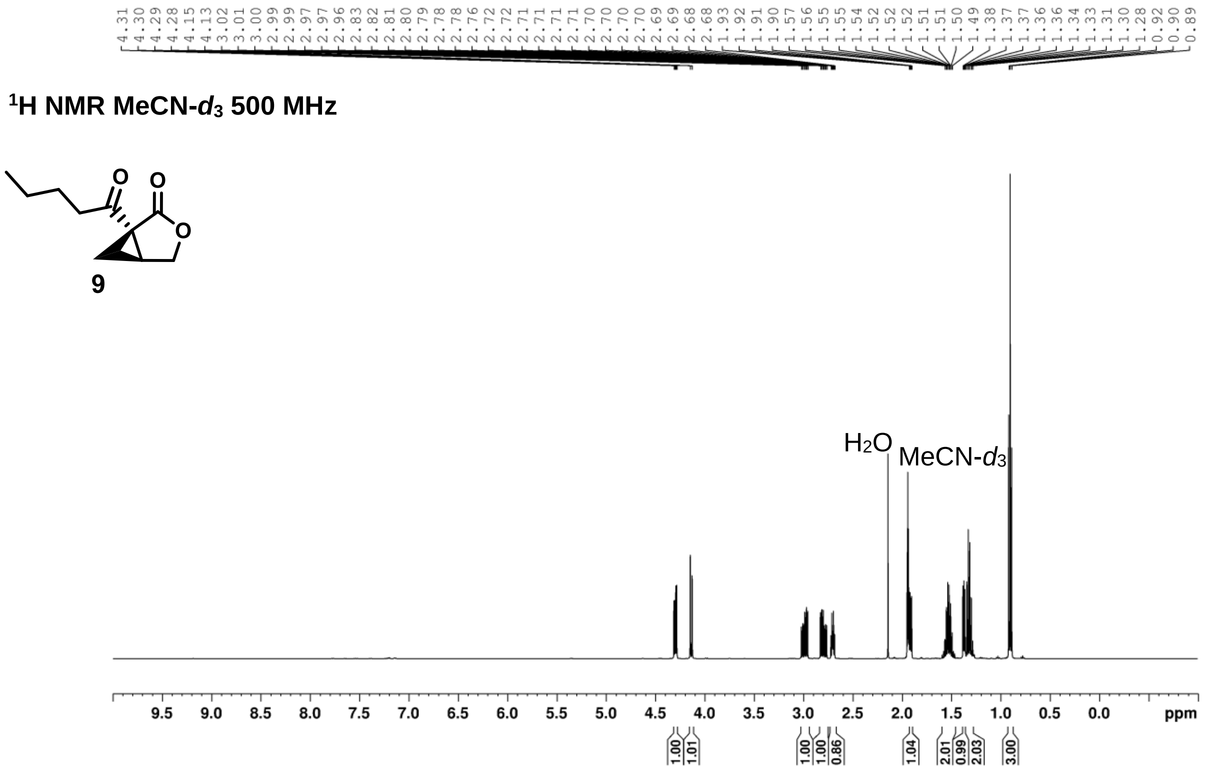

^{13}C NMR MeCN-*d*$_3$ 125 MHz

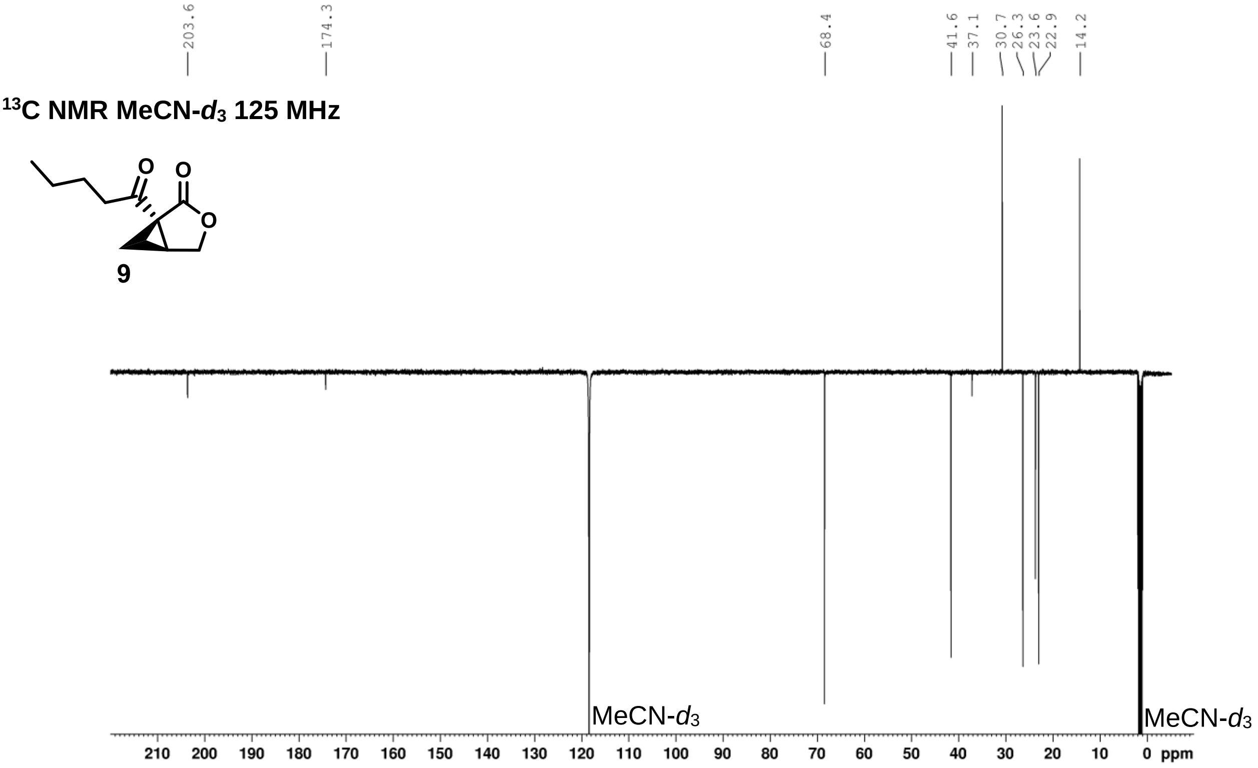

^{1}H NMR MeCN-*d*$_3$ 500 MHz

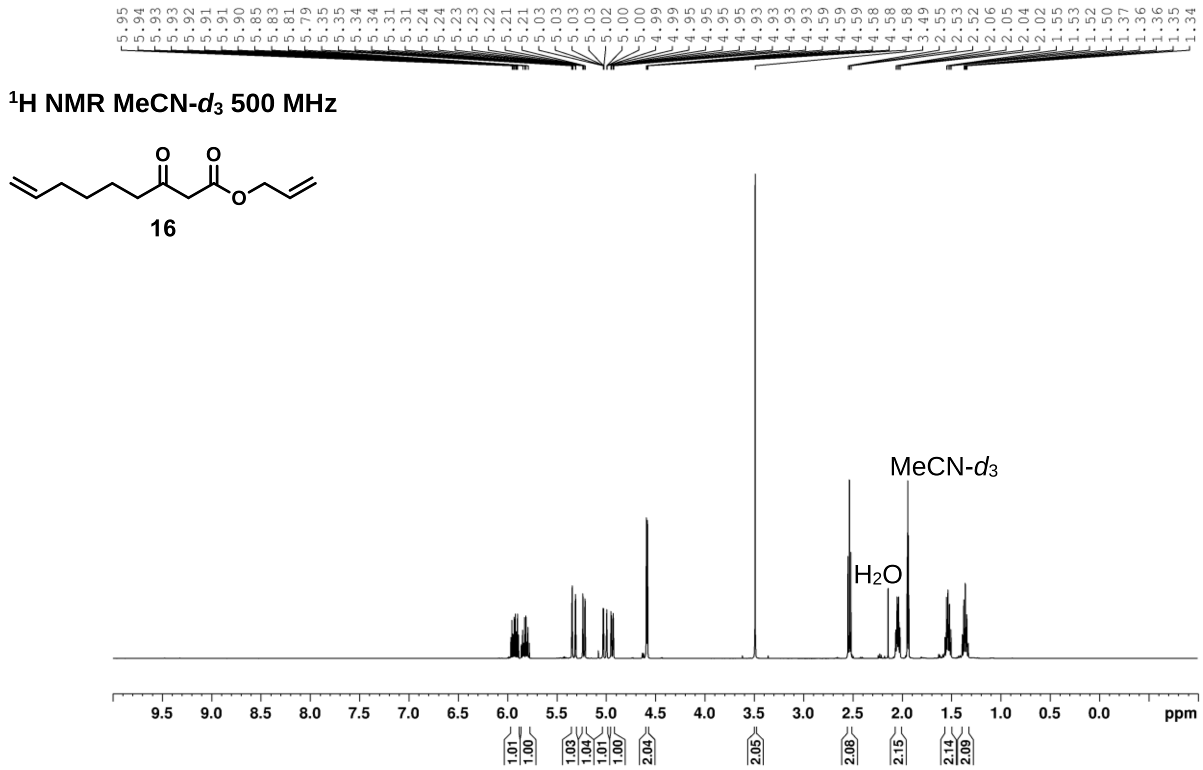

^{13}C NMR MeCN-*d*$_3$ 125 MHz

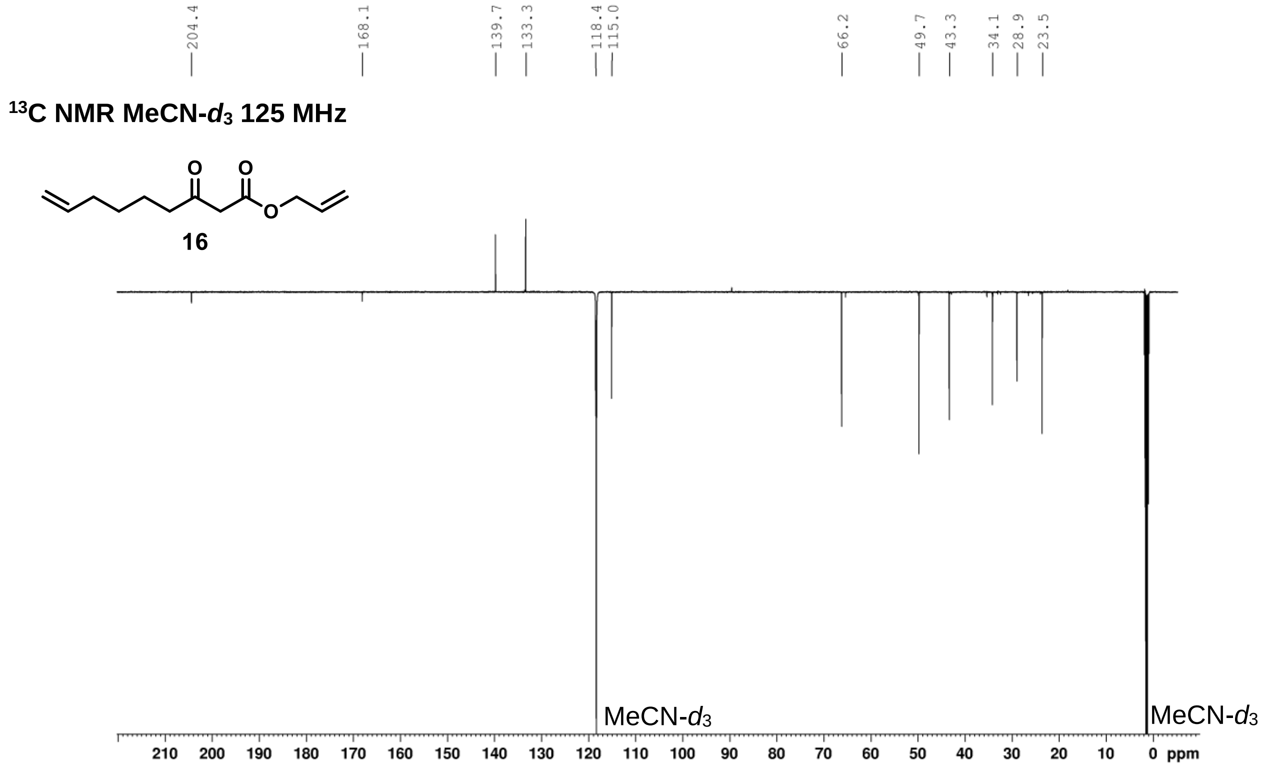

^{1}H NMR MeCN-d_3 500 MHz

17

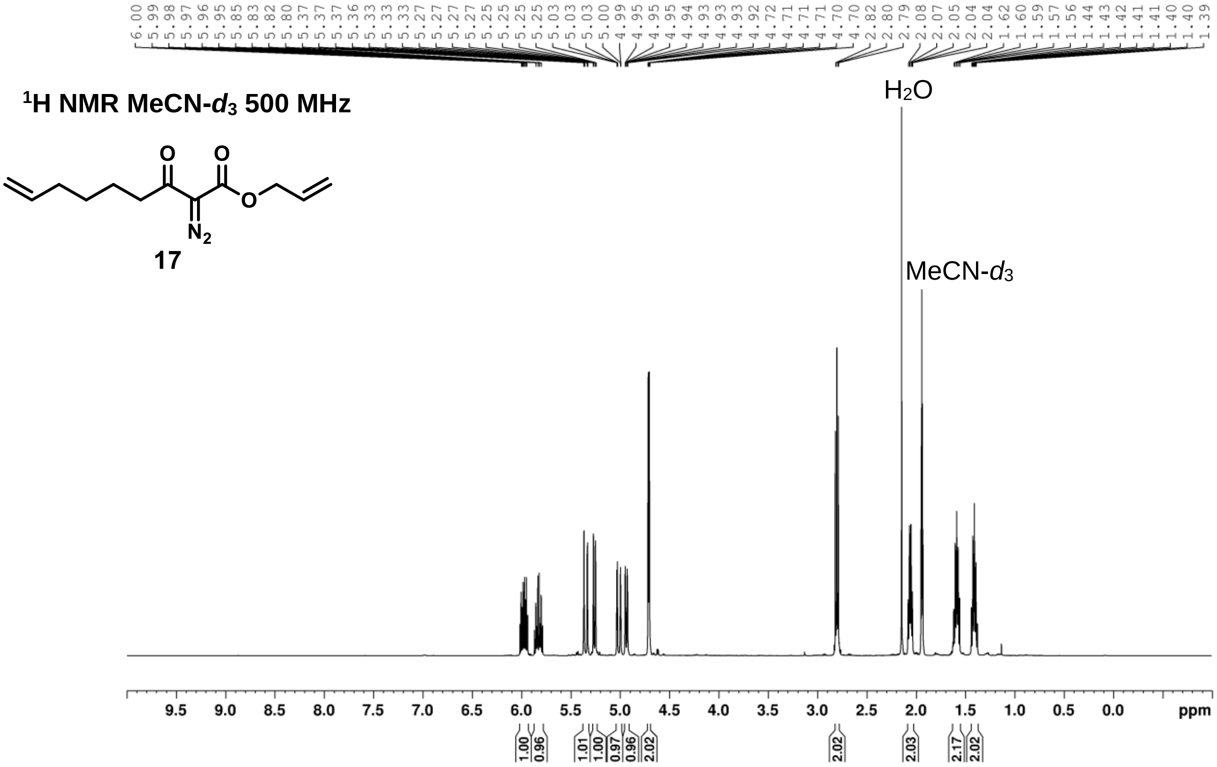

^{13}C NMR DMSO-d_6 125 MHz

17

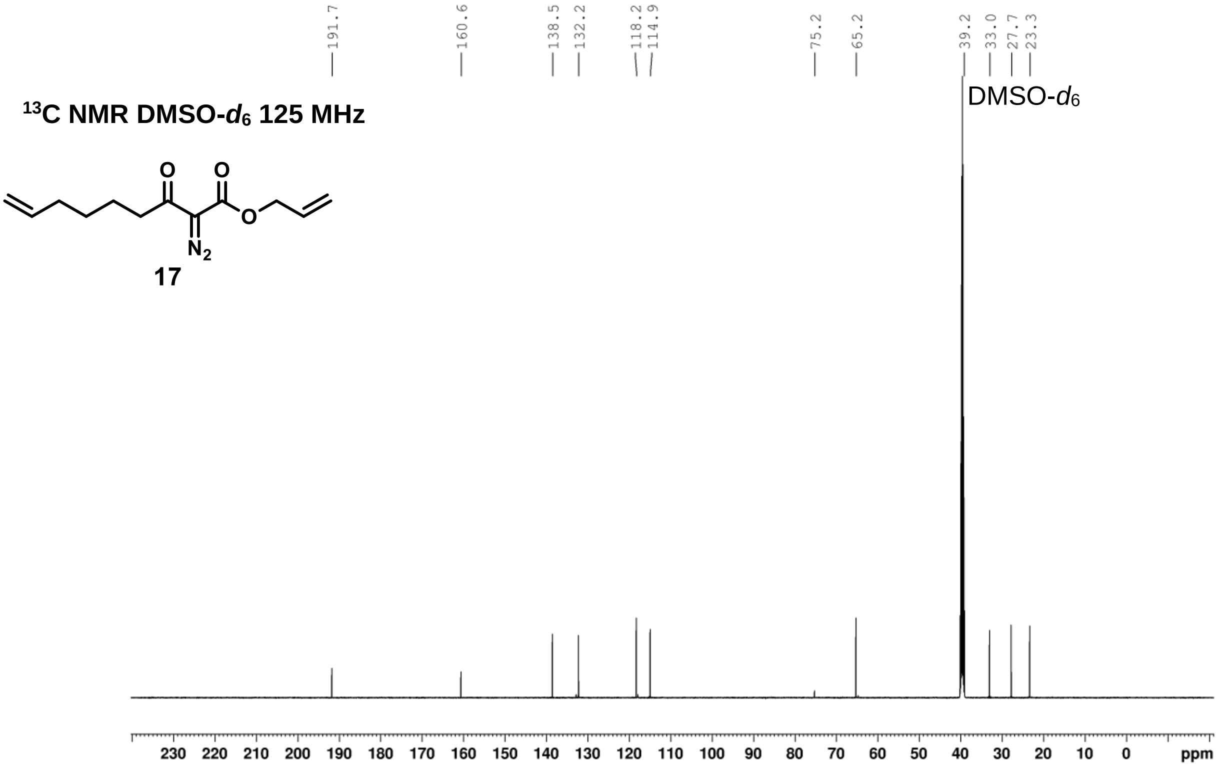

^{1}H NMR MeCN-d_3 500 MHz

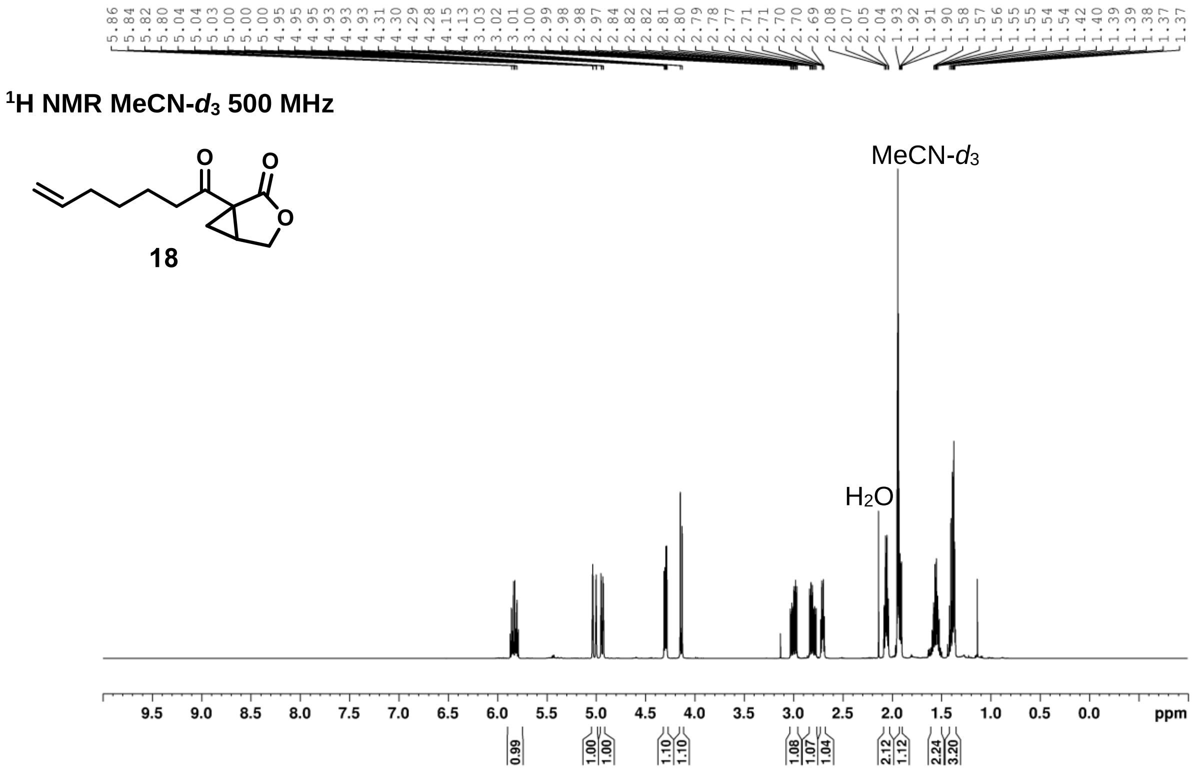

^{13}C NMR MeCN-d_3 125 MHz

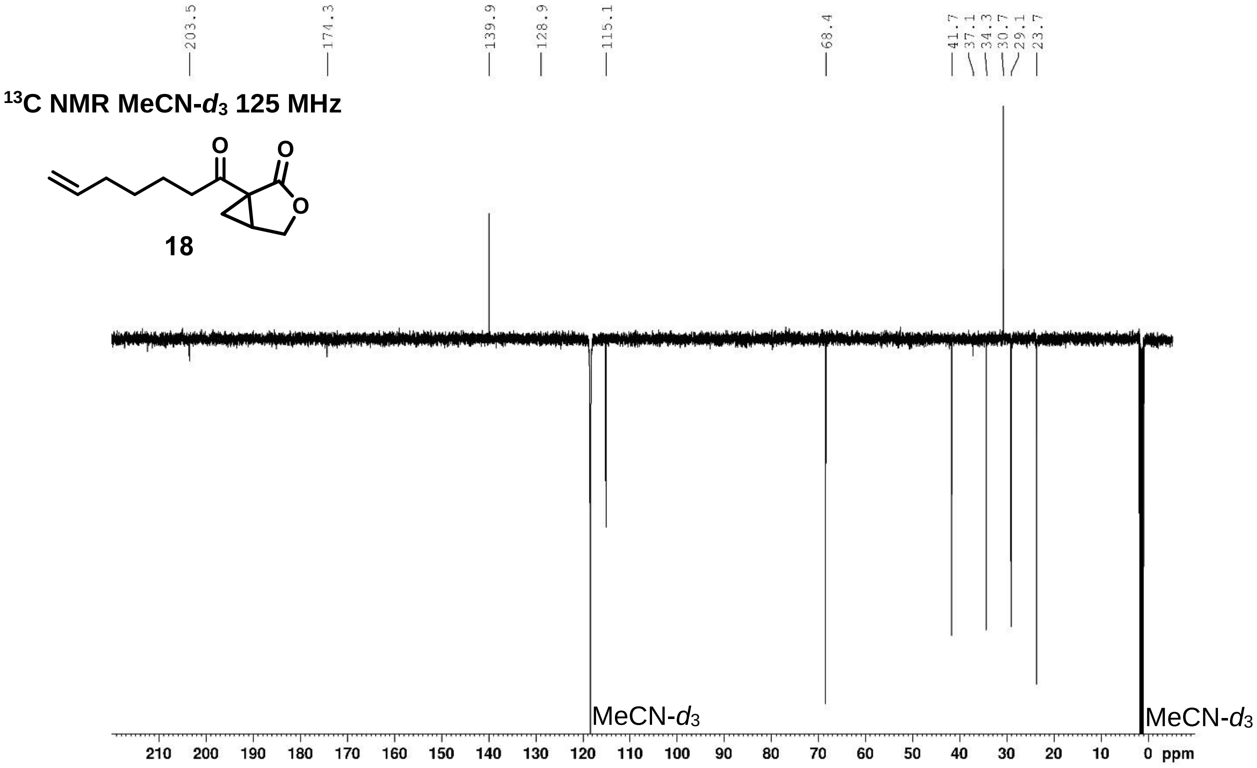

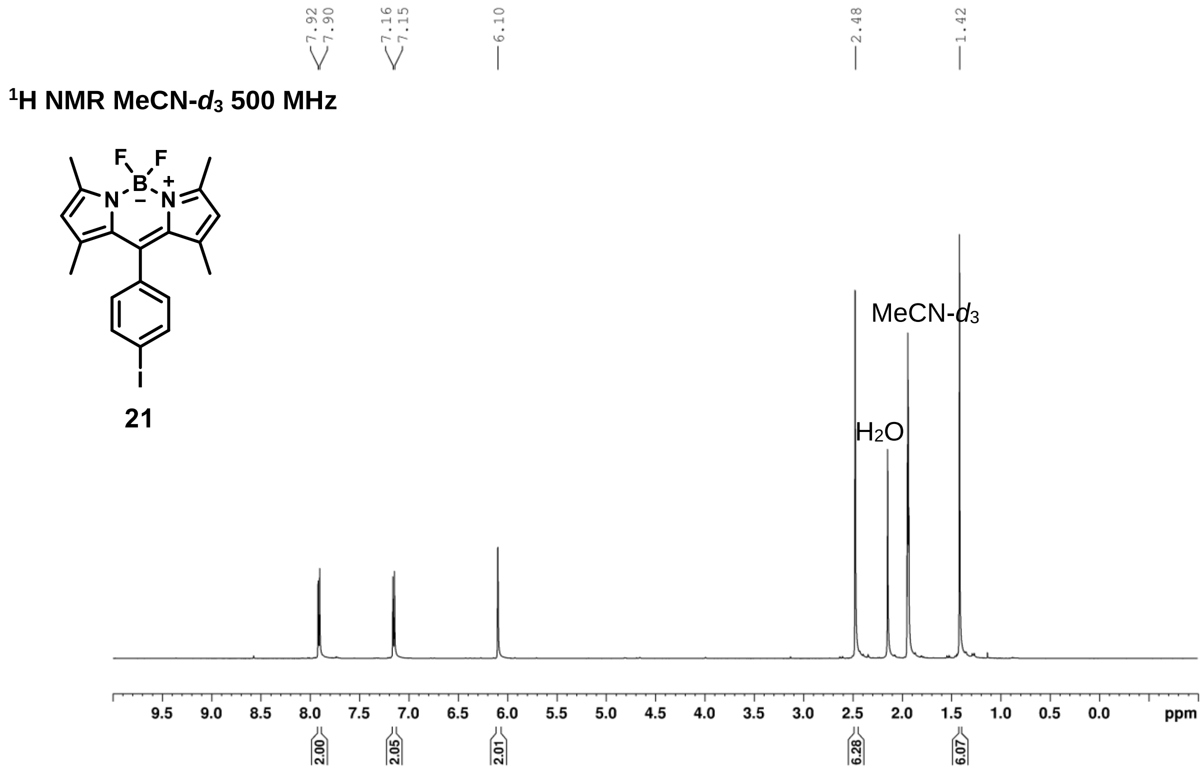
7.92
7.90
7.16
7.15
6.10
2.48
1.42
1H NMR MeCN-d3 500 MHz
21
MeCN-d3
H2O
9.5 9.0 8.5 8.0 7.5 7.0 6.5 6.0 5.5 5.0 4.5 4.0 3.5 3.0 2.5 2.0 1.5 1.0 0.5 0.0 ppm
2.00
2.05
2.01
6.28
6.07

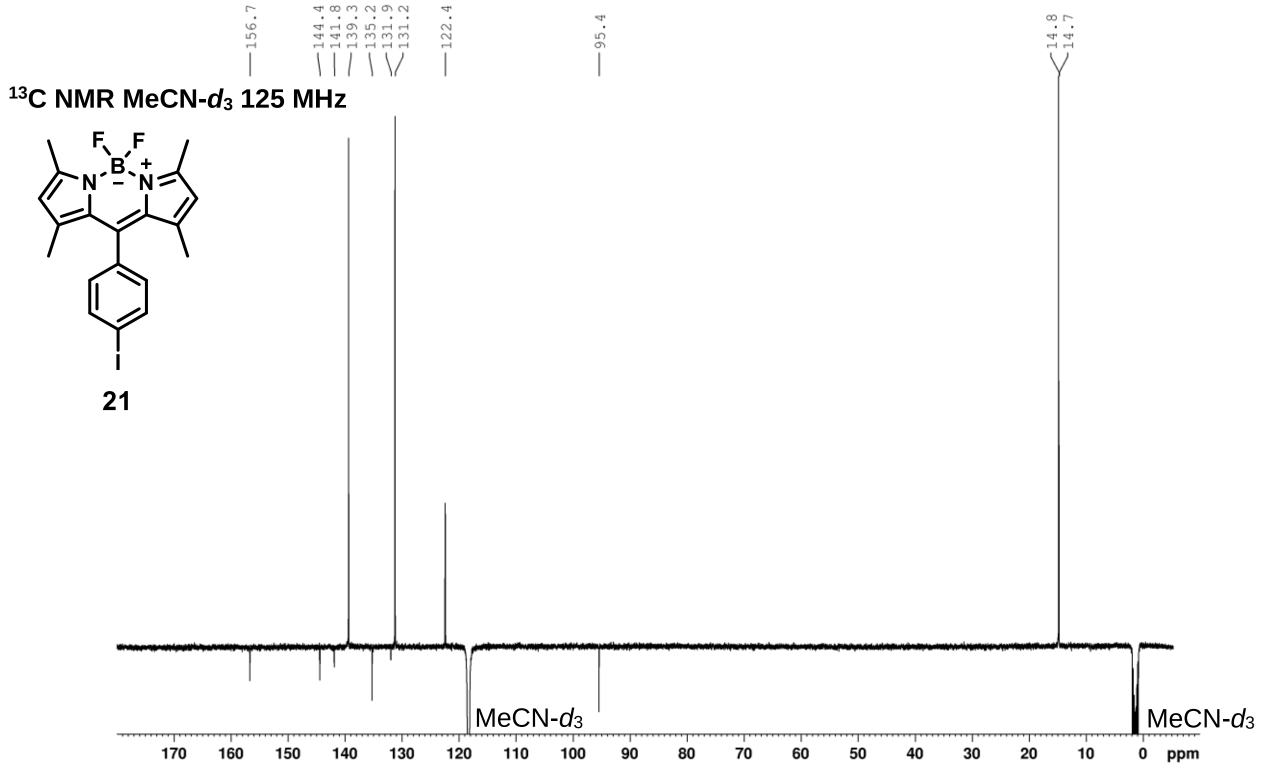
156.7
144.4
141.8
139.3
135.2
131.9
131.2
122.4
95.4
14.8
14.7
13C NMR MeCN-d3 125 MHz
21
MeCN-d3
MeCN-d3
170 160 150 140 130 120 110 100 90 80 70 60 50 40 30 20 10 0 ppm

^{1}H NMR MeCN-d_3 700 MHz

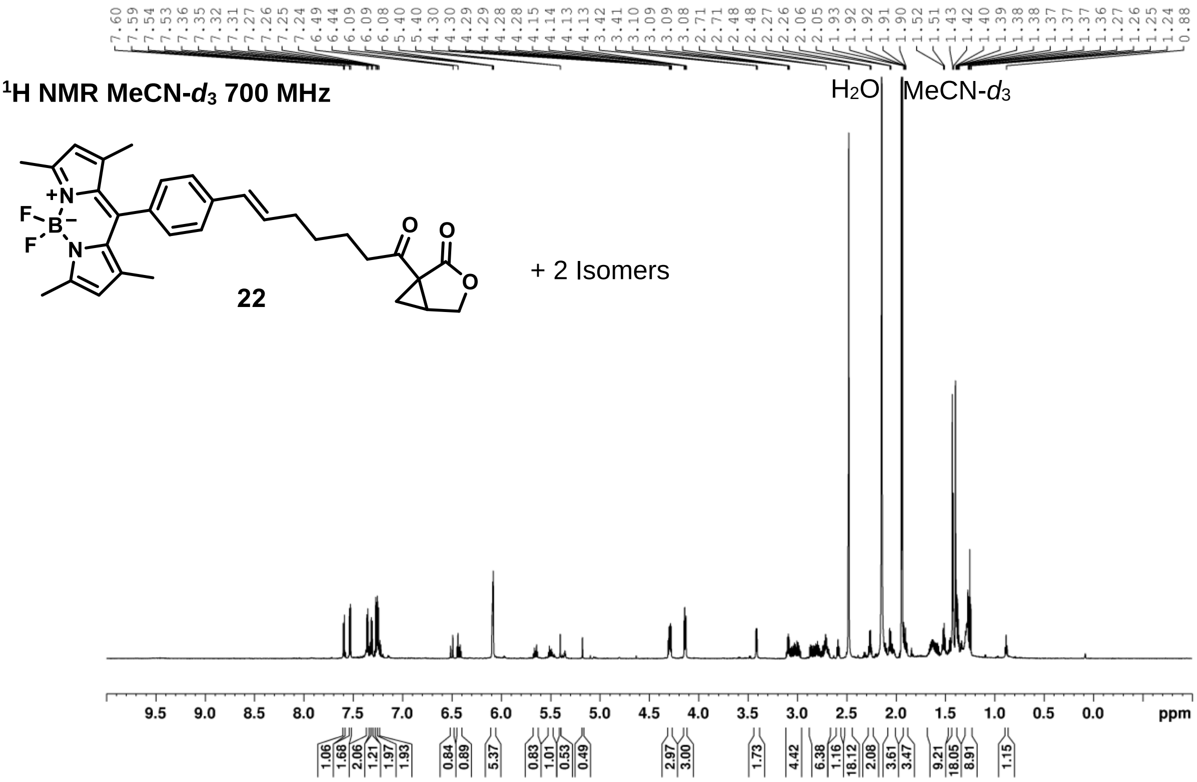

^{13}C NMR MeCN-d_3 175 MHz

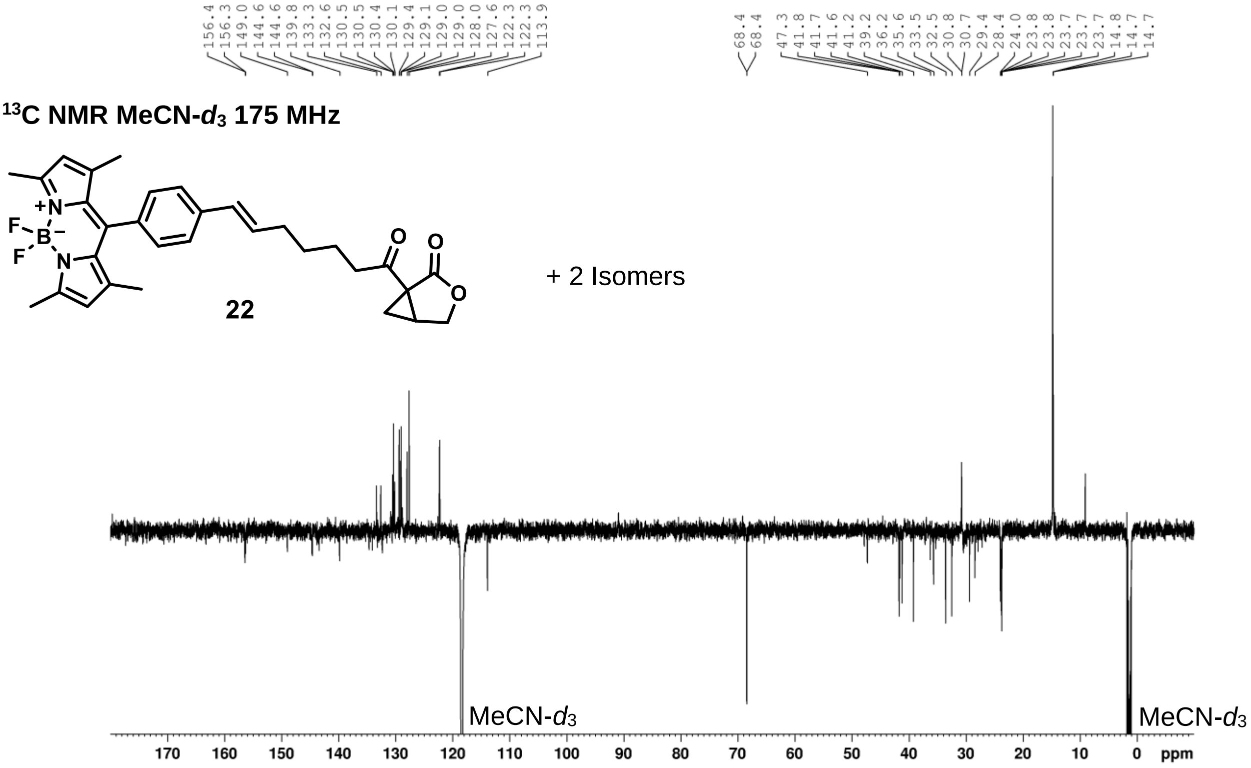

^{1}H NMR MeCN-d_3 500 MHz

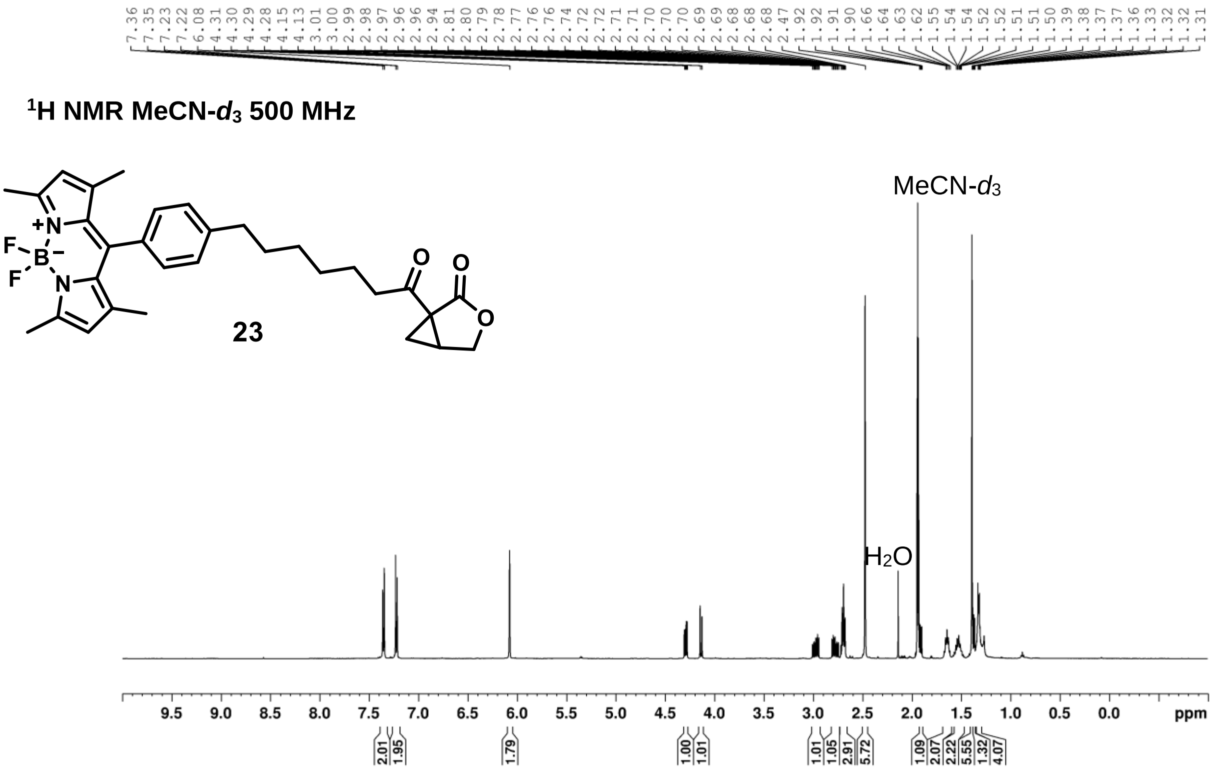

^{13}C NMR MeCN-d_3 125 MHz

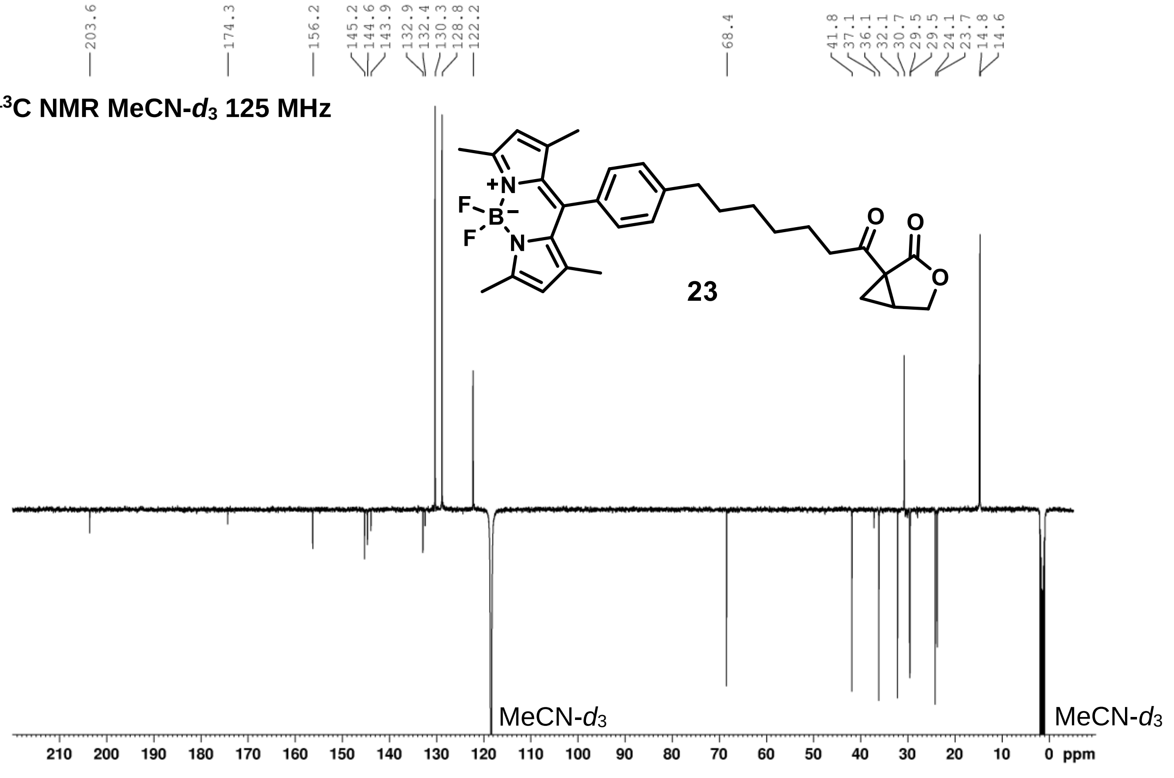

11. Structure elucidation of the ring opening reaction

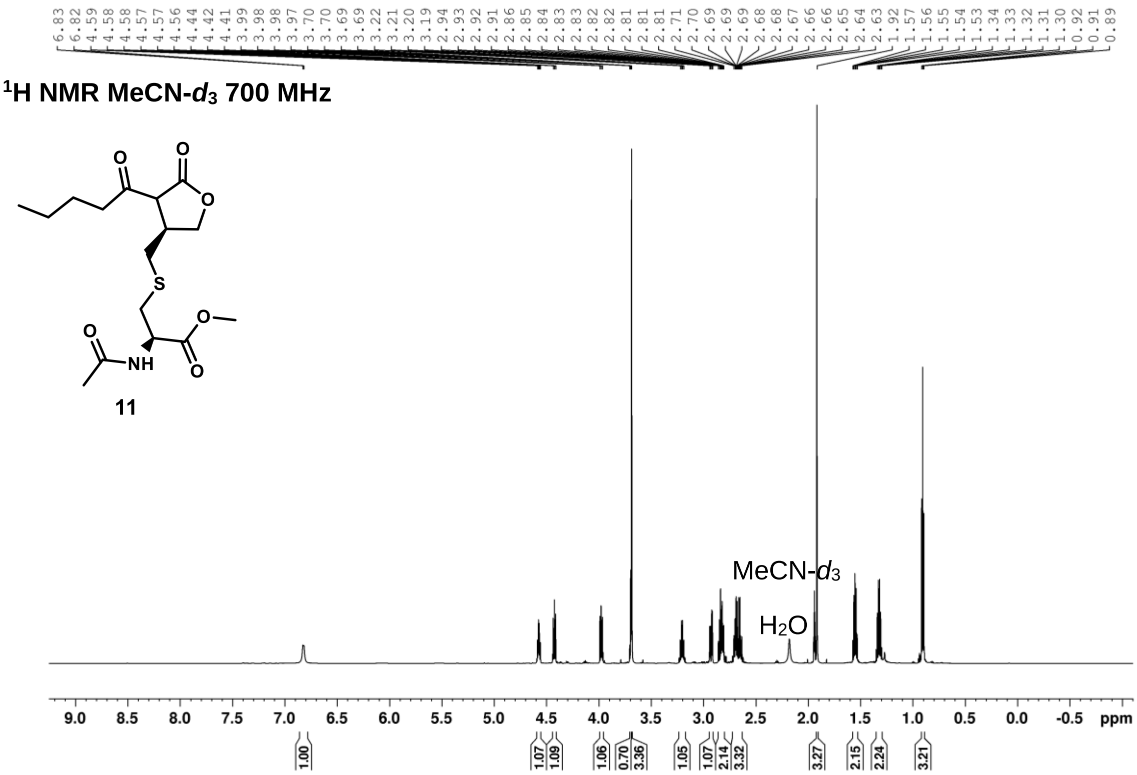

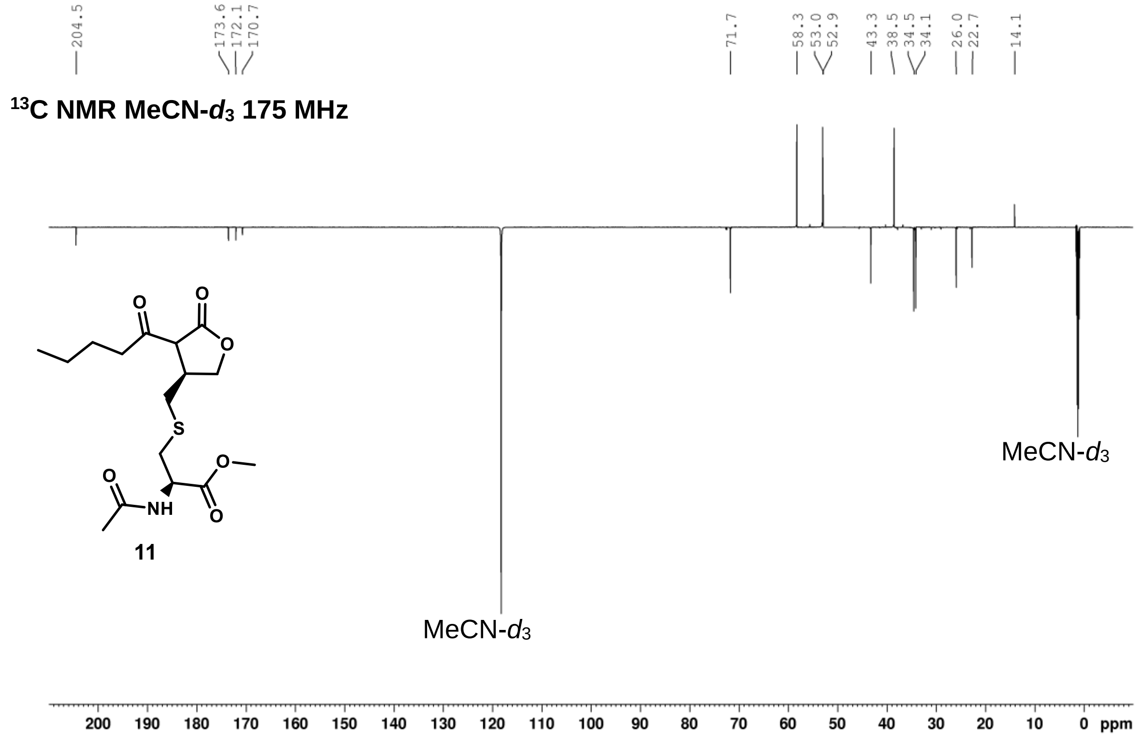

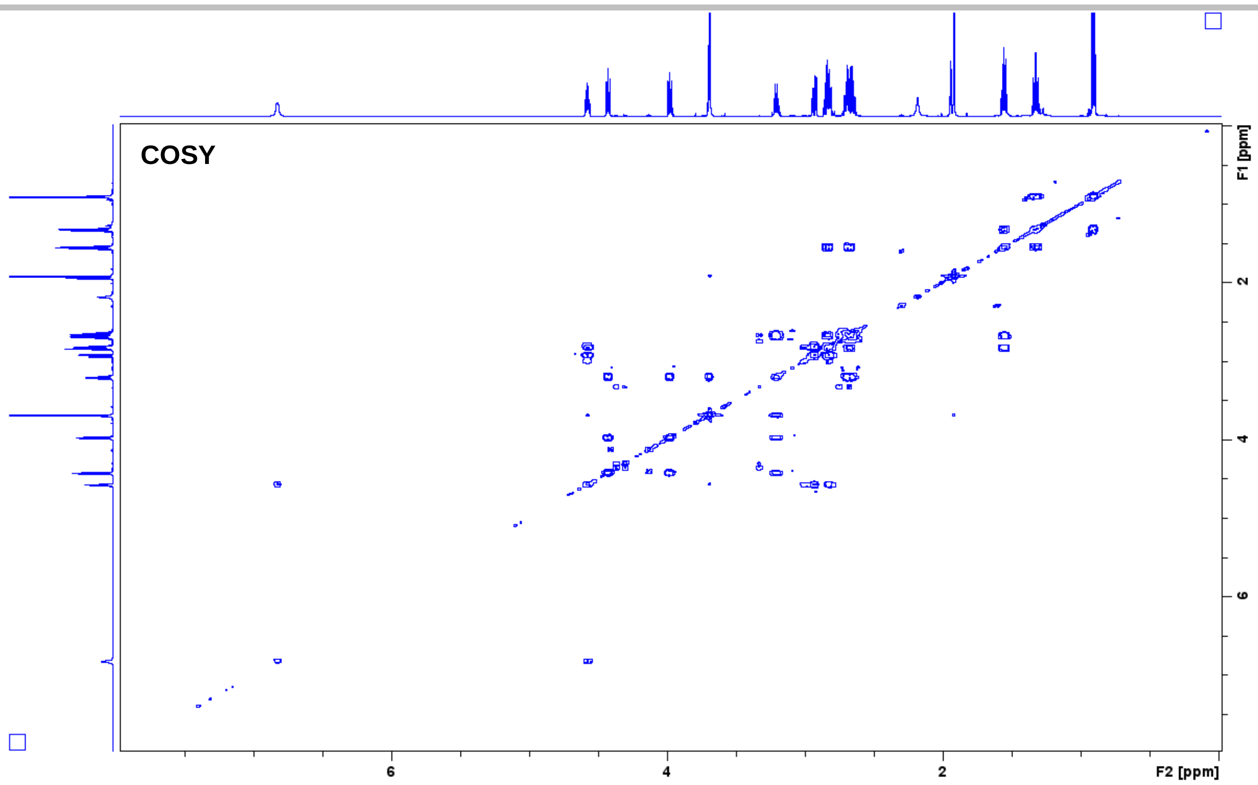
COSY
F1 [ppm]
2
4
6
6
4
2
F2 [ppm]

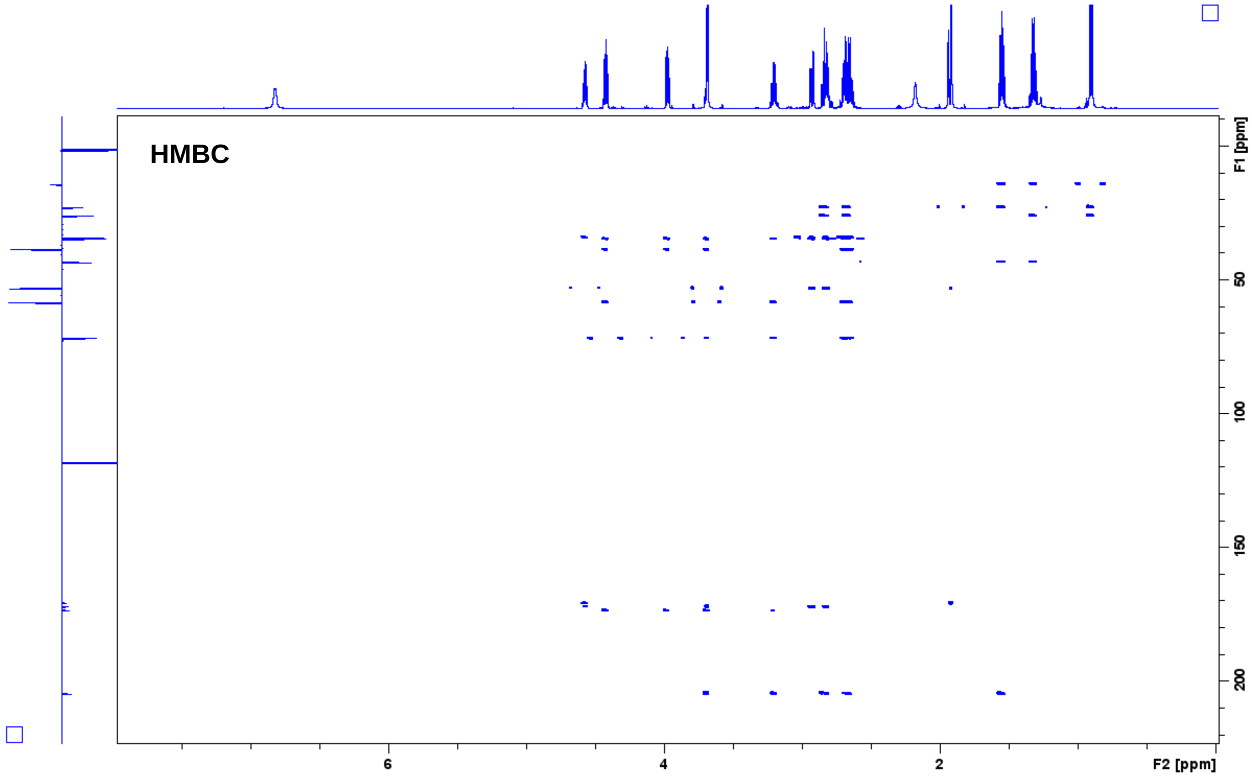
HMBC
F1 [ppm]
50
100
150
200
6
4
2
F2 [ppm]

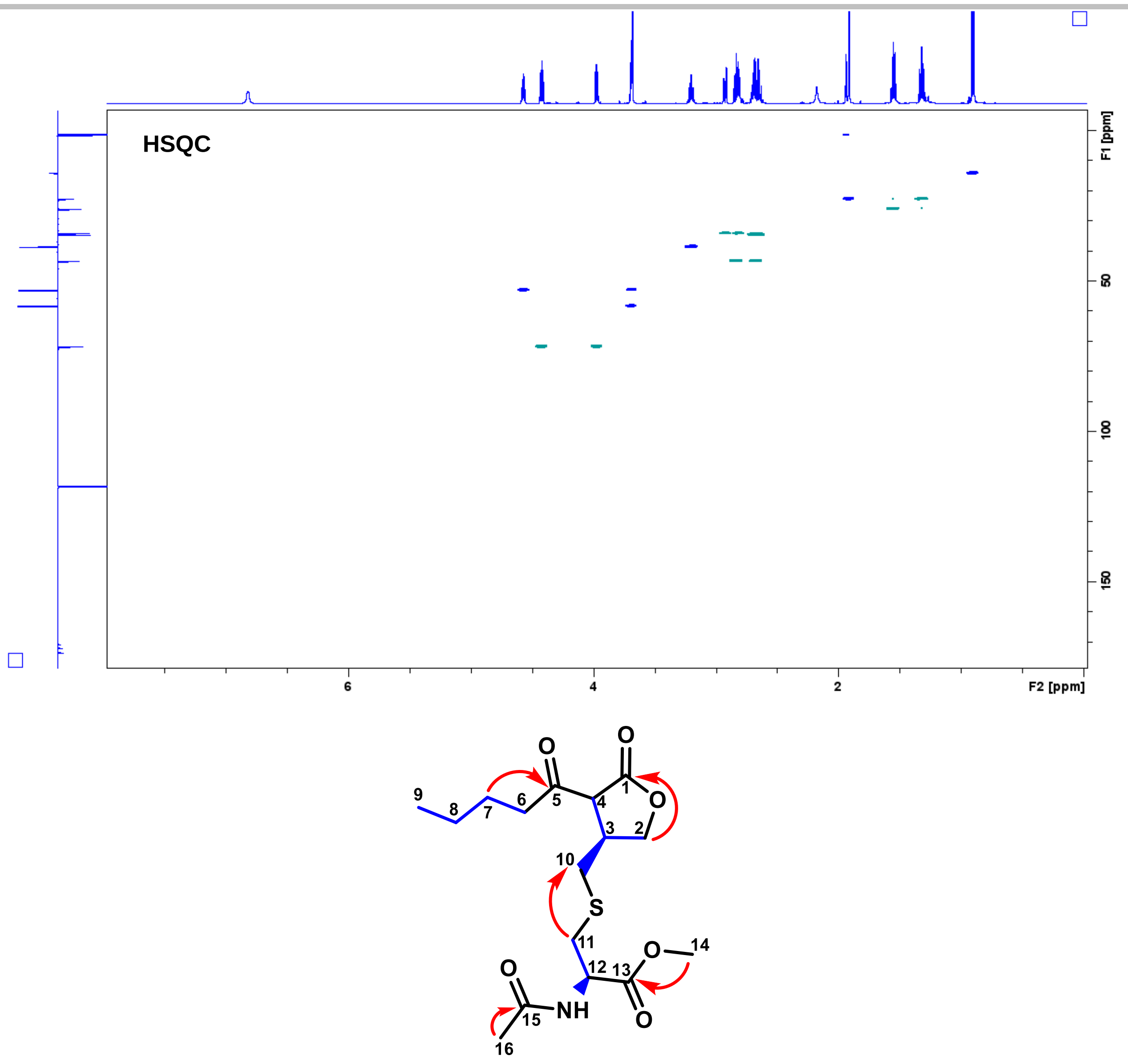

Figure S12. Key correlations in COSY (blue) and HMBC (red arrows).

Table S5. Chemical shifts oh the carbon atoms (175 MHz) and the corresponding proton signals (700 MHz) in MeCN-d_3.

position	δ_C, type	δ_H (*J* in Hz)
1	173.6, C	
2α<br>2β	71.7, CH_2	4.42, m_c<br>3.98, m_c
3	38.5, CH	3.21, m_c
4*	58.3, CH	3.71-3.69, m
5	204.5, C	
6α<br>6β	43.3, CH_2	2.73-2.62, m<br>2.86-2.80, m
7	26.0, CH_2	1.55, quin (7.5)
8	22.7, CH_2	1.32, m_c
9	14.1, CH_3	0.90, t (7.4)
10	34.5, CH_2	2.73-2.62, m
11α<br>11β	34.1, CH_2	2.93, dd (13.8, 5.3)<br>2.86-2.80, m
12	53.0, CH	4.57, m_c
13	172.1, C	
14	52.9, CH_3	3.69, s
15	170.7, C	
16	22.7, CH_3	1.92, s

*The proton of position 4 couples only to one signal in COSY with an integral of 1. If it would be a six-membered ring, it should couple to a signal of an integral with 2.

12. References

[1] Y. Perez-Riverol, J. Bai, C. Bandla, D. García-Seisdedos, S. Hewapathirana, S. Kamatchinathan, Deepti J. Kundu, A. Prakash, A. Frericks-Zipper, M. Eisenacher, M. Walzer, S. Wang, A. Brazma, Juan A. Vizcaíno, *Nucleic Acids Res.* **2022**, *50*, D543-D552.
[2] N. Dyballa, S. Metzger, *J. Vis. Exp.* **2009**, 1-4.
[3] F. Ghanbari, K. Rowland-Yeo, J. C. Bloomer, S. E. Clarke, M. S. Lennard, G. T. Tucker, A. Rostami-Hodjegan, *Curr. Drug. Metab.* **2006**, *7*, 315-334.
[4] C. Schlawis, *Infrarotspektroskopie von Naturstoffen* **2020**.
[5] C. Schlawis, S. Kern, Y. Kudo, J. Grunenberg, B. S. Moore, S. Schulz, *Angew. Chem. Int. Ed.* **2018**, *57*, 14921-14925.
[6] D. Jakubczyk, C. Merle, G. Brenner-Weiss, B. Luy, S. Bräse, *Eur. J. Org. Chem.* **2013**, *2013*, 5323-5330.
[7] X. Li, R. Pan, **2021**, US 2021/0277027 A0277021.
[8] C. Walter, L. Candiani, *Acc. Chem. Res.* **1995**, *28*, 2-7.
[9] C. Amatore, A. Jutand, *Acc. Chem. Res.* **2000**, *33*, 314-321.
[10] T. J. M. Beenakker, D. P. A. Wander, J. D. C. Codée, J. M. F. G. Aerts, G. A. van der Marel, H. S. Overkleeft, *Eur. J. Org. Chem.* **2018**, *2018*, 2504-2517.
